# Runx proteins mediate protective immunity against *Leishmania donovani* infection by promoting CD40 expression on dendritic cells

**DOI:** 10.1371/journal.ppat.1009136

**Published:** 2020-12-28

**Authors:** Md. Naushad Akhtar, Manish Mishra, Vinod Yadav, Manisha Yadav, Ravindra Gujar, Sunaina Lal, Raj Kumar, Neeraj Khatri, Pradip Sen

**Affiliations:** 1 Division of Cell Biology and Immunology, Council of Scientific and Industrial Research- Institute of Microbial Technology, Chandigarh, India; 2 IMTECH-Centre for Animal Resources and Experimentation (iCARE), Council of Scientific and Industrial Research-Institute of Microbial Technology, Chandigarh, India; INRS - Institut Armand Frappier, CANADA

## Abstract

The level of CD40 expression on dendritic cells (DCs) plays a decisive role in disease protection during *Leishmania donovani* (LD) infection. However, current understanding of the molecular regulation of CD40 expression remains elusive. Using molecular, cellular and functional approaches, we identified a role for Runx1 and Runx3 transcription factors in the regulation of CD40 expression in DCs. In response to lipopolysaccharide (LPS), tumor necrosis factor alpha (TNFα) or antileishmanial drug sodium antimony gluconate (SAG), both Runx1 and Runx3 translocated to the nucleus, bound to the *CD40* promoter and upregulated CD40 expression on DCs. These activities of Runx proteins were mediated by the upstream phosphatidylinositol 3-kinase (PI3K)-Akt pathway. Notably, LD infection attenuated LPS- or TNFα-induced CD40 expression in DCs by inhibiting PI3K-Akt-Runx axis via protein tyrosine phosphatase SHP-1. In contrast, CD40 expression induced by SAG was unaffected by LD infection, as SAG by blocking LD-induced SHP-1 activation potentiated PI3K-Akt signaling to drive Runx-mediated CD40 upregulation. Adoptive transfer experiments further showed that Runx1 and Runx3 play a pivotal role in eliciting antileishmanial immune response of SAG-treated DCs *in vivo* by promoting CD40-mediated type-1 T cell responses. Importantly, antimony-resistant LD suppressed SAG-induced CD40 upregulation on DCs by blocking the PI3K-Akt-Runx pathway through sustained SHP-1 activation. These findings unveil an immunoregulatory role for Runx proteins during LD infection.

## Author summary

Visceral leishmaniasis (VL), caused by *Leishmania donovani*, is the second most deadly parasitic disease in the world after malaria. The protective immunity against this disease largely depends on the level of CD40 (a cell surface protein) expression on a specialized group of immune cells called “dendritic cells” (DCs), which play a central role in initiating antileishmanial immune responses. Accordingly, to develop an improved immunological approach for the treatment/prevention of VL, it is important to understand the molecular events that favor the induction of CD40 expression on DCs. However, the molecular mechanisms controlling CD40 expression are not yet fully understood. Our work describes, for the first time, that the transcription factors Runx1 and Runx3 promote CD40 expression on DCs, which thereby potentiate the ability of DCs to induce antileishmanial immune responses *in vivo*. Our study thus provides new insights into the molecular mechanism required for an immune response to combat VL.

## Introduction

Dendritic cells (DCs) have emerged as key regulators of host immune response during visceral leishmaniasis (VL), a potentially fatal human disease caused by *Leishmania donovani* (LD). Besides functioning as initiators of *Leishmania-*specific T cell reactivity, DCs play a central role in the regulation of host-protective T helper cell type-1 (Th1) responses [1,2]. This regulatory potential of DCs is largely influenced by the level of CD40 expression on their surface [3]. CD40, a member of tumor necrosis factor receptor (TNFR) family, is an important costimulatory molecule for DCs [4]. Interaction between CD40 on DCs and CD40 ligand (CD40L) on T cells induces DCs to produce interleukin (IL)-12 [3]. Indeed, DCs are the only source of early IL-12 production following LD infection [5]. DC-derived IL-12 then skews differentiation of naïve CD4^+^ T cells to interferon gamma (IFNγ)-producing Th1 cells and thereby mounts protective immunity against LD infection [2,6]. This scenario occurs only when CD40 is expressed at higher levels on DCs [7]. The low CD40 expression on DCs, on the other hand, favors the generation of regulatory T (Treg) cells that exacerbates the disease [7]. Thus, the level of CD40 expression on DCs plays an important role in determining the disease outcome during LD infection. However, the molecular mechanisms regulating CD40 expression in DCs have not been fully elucidated. So far, only the role of NF-κB, Sp1, and STAT-1 transcription factors in lipopolysaccharide (LPS)- and leptin-mediated regulation of CD40 expression in DCs are known [8,9]. Therefore, identification of new transcriptional regulators for *CD40* and deciphering their roles in LD-mediated regulation of CD40 expression in DCs may provide new insights into the immunoregulatory events involved in VL.

The runt-related (Runx) family of transcription factors plays multiple roles in immune regulation [10]. The Runx family consists of three members Runx1, Runx2 and Runx3 [10]. The role for Runx transcription factors in DC immunobiology has been described by limited number of studies. For instance, Runx3 knockout DCs show accelerated maturation and are resistant to transforming growth factor-β (TGF-β)-induced maturation inhibition [11]. In addition, an increased chemokine receptor-7 (CCR7)-mediated DC migration in Runx3 knockout mice leads to the development of asthma-like disease [12].

A previous report has shown that Runx3 promotes Th1 differentiation by simultaneously augmenting IFNγ expression and attenuating IL-4 expression [13]. Notably, the Th1 differentiation is also influenced by the level of CD40 expression on DCs [3]. However, it is not yet known whether Runx proteins regulate CD40 expression in DCs. Furthermore, the immunoregulatory roles for Runx proteins during *Leishmania* infection remain undefined. In this study, we therefore addressed following three issues: whether Runx proteins regulate CD40 expression in DCs, and if so, then what are the upstream signaling events controlling this regulatory activity of Runx; whether and how LD modulates CD40 expression in DCs by regulating Runx activity; and finally examined whether Runx proteins, by regulating CD40 expression on DCs, influence antileishmanial immune responses.

## Results

### LD downregulates LPS- and TNFα-stimulated CD40 expression on DCs

**Fig 1:**
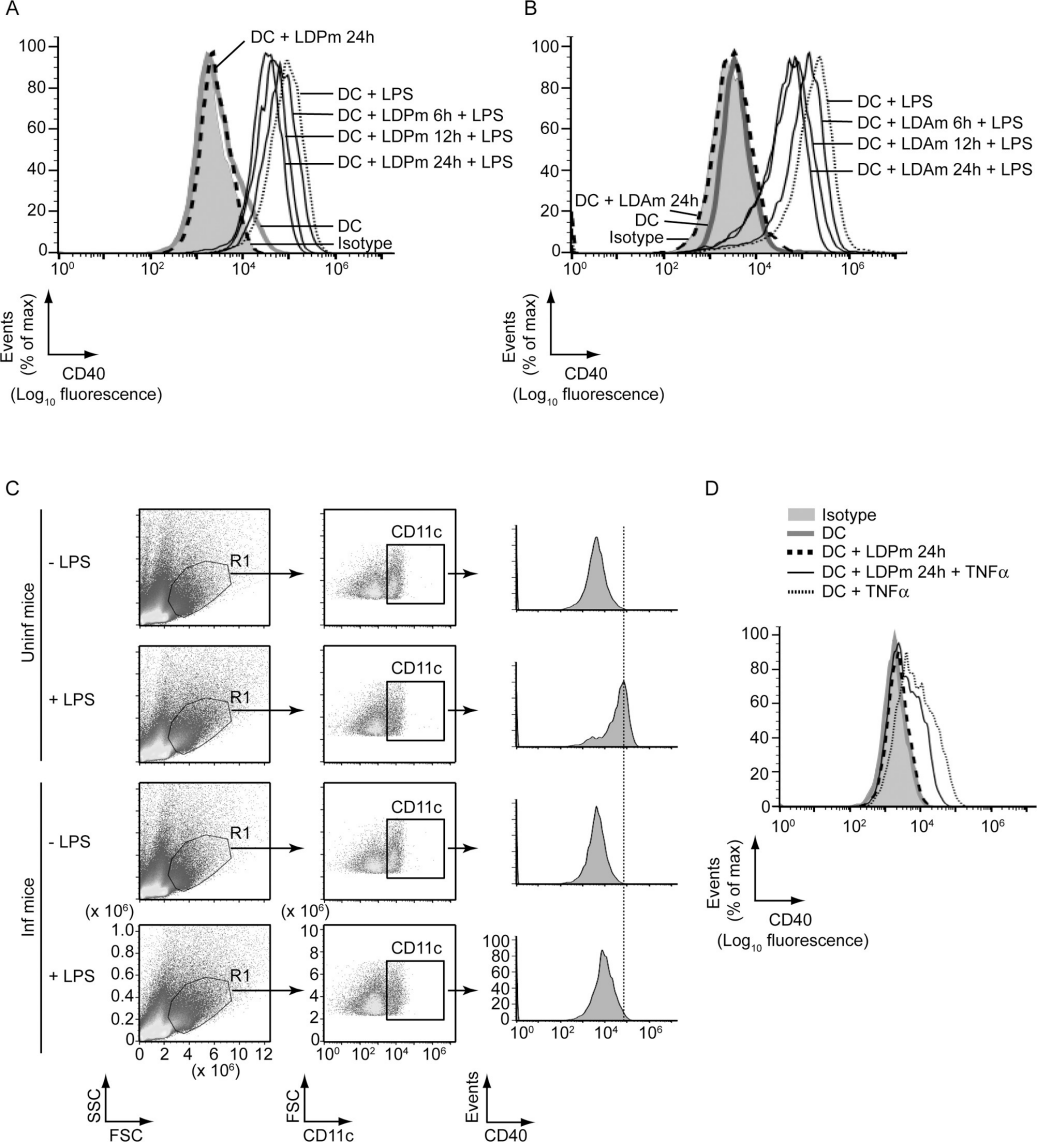
LD inhibits LPS- and TNFα-stimulated CD40 upregulation on DCs. (A and B) Expression of CD40 on BMDCs left uninfected (DC) or infected with LD promastigotes (LDPm; A) or amastigotes (LDAm; B) for indicated times and then stimulated with LPS for 24 h; evaluated by flow cytometry. Isotype represents immunostaining with an isotype-matched control antibody. (C) Splenocytes from uninfected (Uninf) and LD-infected (Inf) mice were cultured (at 45 days postinfection) in the presence or absence of LPS for 24 h. CD40 expression on DCs (CD11c^+^ gated cells outlined in the middle panels) was analyzed by flow cytometry. (D) Flow cytometry analysis of CD40 expression by BMDCs left uninfected or infected for 24 h with LDPm and then stimulated with TNFα for 24 h. In all experiments, LD strain AG83 was used. Data are representative of three independent experiments. For (A), (B) and (D), the relative mean fluorescence intensity (MFI) values of CD40 expression pooled from three independent experiments are shown in S1 Fig.

Because available reports have provided contradictory results regarding regulation of CD40 expression by LD in DCs [14–18], we first clarified whether LD upregulates or downregulates CD40 expression in DCs. For this purpose, we infected bone marrow-derived DCs (BMDCs), established from BALB/c mice, with LD promastigotes (extracellular form; LDPm) or amastigotes (intracellular form; LDAm) for varying times (6 h, 12 h or 24 h), stimulated with LPS for 24 h and assessed CD40 expression via flow cytometry. Relative to untreated BMDCs, BMDCs treated with LPS displayed more CD40 expression (Fig 1A and 1B, S1A and S1B Fig). In contrast, BMDC infection with LDPm or LDAm resulted in a temporal reduction in LPS-stimulated CD40 expression, with maximum inhibition occurring at 24 h postinfection (Fig 1A and 1B, S1A and S1B Fig). The LPS-stimulated CD40 expression was also found to be lower on splenic DCs (sDCs) derived from LD-infected mice than those derived from uninfected mice (Fig 1C). Further, to verify whether LD inhibits CD40 expression induced by other DC maturation stimuli, we analyzed the effect of LD infection on tumor necrosis factor alpha (TNFα)-stimulated CD40 expression. For these experiments, we infected BMDCs with LDPm for 24 h because our aforementioned observations (Fig 1A, S1A Fig) demonstrated maximum downregulation of LPS-induced CD40 expression at 24 h post LDPm infection. Subsequently, we stimulated these LDPm-infected BMDCs with TNFα for 24 h and analyzed CD40 expression on BMDCs via flow cytometry. We found that BMDC infection with LDPm for 24 h also inhibited the CD40 upregulation induced by TNFα (Fig 1D, S1C Fig). Thus, regardless of the forms of LD parasite (i.e., extracellular or intracellular form), LD inhibits LPS- and TNFα-stimulated CD40 expression in DCs.

### Runx proteins promote CD40 upregulation on DCs

**Fig 2:**
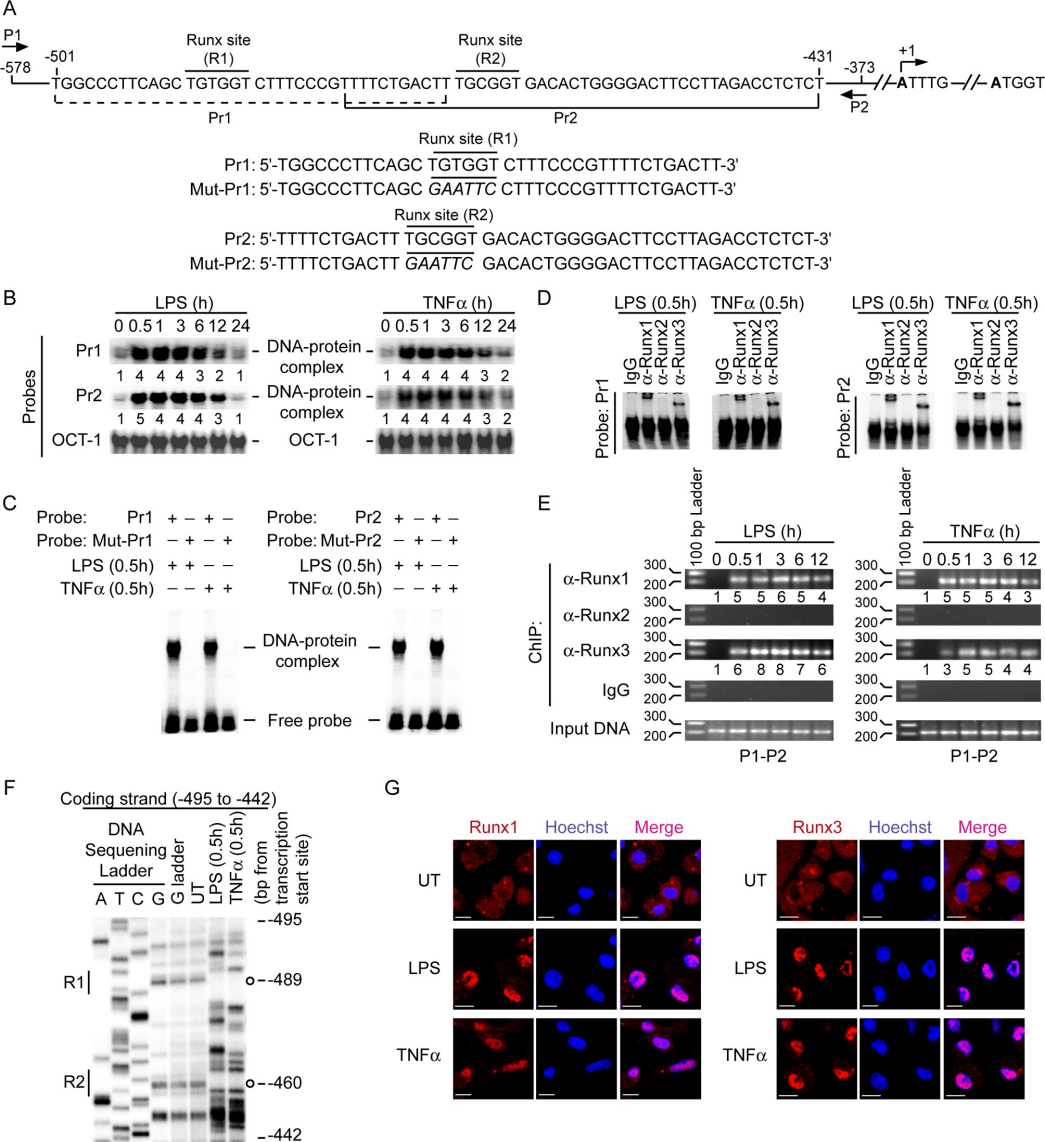
LPS and TNFα induce nuclear localization and binding of Runx proteins to the *CD40* promoter. (A) Schematic presentation of mouse *CD40* promoter indicating the location of putative Runx-binding sites (R1 and R2) and ChIP primers (P1 and P2), and details of oligonucleotide probes used for EMSA. Mouse *CD40* promoter-specific Pr1 and Pr2 probes contain putative wild-type Runx-binding sites, and Mut-Pr1 and Mut-Pr2 probes contain mutations (in italics) at Runx-binding sites. Base positions are relative to the transcription start site. (B and C) EMSA of nuclear extracts of BMDCs treated with LPS or TNFα for indicated times; assessed with indicated probes. Numbers below lanes in (B) represent densitometry [normalized to OCT-1 binding (control)] relative to that of untreated BMDCs (0 h). The densitometry results pooled from three independent experiments for (B) are shown in S2 Fig. (D) Supershift EMSA [with immunoglobulin G (IgG; control) or antibody to (α-) Runx1, Runx2 or Runx3] of nuclear extracts of BMDCs treated with LPS or TNFα for 0.5 h; assayed with Pr1 or Pr2 probes. (E) Binding of Runx proteins to -578/-373 region of the *CD40* promoter in BMDCs treated with LPS or TNFα (time, above lanes); assessed via ChIP using the primers shown in (A) and indicated antibodies (left margin). Amplification of mouse *GAPDH* promoter (S4 Fig) and chromatin immunoprecipitated by rabbit IgG were used as negative controls, and input DNA (2%) as an internal control. Numbers below lanes represent densitometry, normalized to input DNA and presented relative to that of untreated BMDCs (0 h). (F) *In vivo* footprint analysis of mouse *CD40* promoter showing DMS-protected bands (indicated by open circles) at R1 and R2 sites in LPS- or TNFα-treated but not in untreated (UT) BMDCs. (G) Translocation of Runx1 or Runx3 (red) into the nuclei (blue; Hoechst staining) in BMDCs left untreated (UT) or treated with LPS or TNFα for 0.5 h; assessed by confocal microscopy. Pink color (merge) shows nuclear translocation of Runx1 or Runx3. Scale bar, 10 μm. Confocal microscopy images of DCs immunostained with isotype control antibodies are given in S5 Fig. Data are representative of three (B-E) or two (F and G) independent experiments.

Before determining how LD suppressed LPS- or TNFα-stimulated CD40 expression in DCs, we sought to identify a previously unrecognized mechanism associated with transcriptional regulation of *CD40* by LPS and TNFα. While examining the mouse *CD40* promoter sequence with the TFBIND program, we found two potential Runx-binding sites (R1: ^-489^TGTGGT^-484^ and R2: ^-464^TGCGGT^-459^; base positions are relative to the transcription initiation site) (Fig 2A). Accordingly, we tested whether LPS and TNFα induce the binding of Runx proteins to the *CD40* promoter in DCs. Electrophoretic mobility shift assay (EMSA) using mouse *CD40* promoter-specific Pr1 and Pr2 probes, which contained R1 and R2 sites, respectively (Fig 2A), demonstrated an increased binding of nuclear proteins to each of these probes upon stimulation of BMDCs and sDCs with LPS or TNFα (Fig 2B, S2 and S3 Figs). This nuclear protein binding, however, was blocked by mutation of the Runx-binding sequences (Fig 2C). Antibody-mediated supershift analysis subsequently affirmed the binding of Runx1 and Runx3 to Pr1 and Pr2 probes in LPS- or TNFα-treated DCs (Fig 2D). Correspondingly, chromatin immunoprecipitation (ChIP) assays demonstrated an enhanced recruitment of Runx1 and Runx3 to the *CD40* promoter following LPS or TNFα stimulation (Fig 2E, S4 Fig). In addition, *in vivo* footprint analysis showed protected (i.e., weaker) bands within R1 and R2 sites of the mouse *CD40* promoter in LPS- and TNFα-treated BMDCs but not in untreated BMDCs (Fig 2F), which is indicative of the occupancy of both R1 and R2 sites by Runx proteins following LPS or TNFα stimulation. Confocal microscopic studies further demonstrated that BMDC stimulation with LPS or TNFα augmented nuclear translocation of Runx1 and Runx3 (Fig 2G, S5 Fig). Thus, LPS and TNFα trigger the nuclear translocation and subsequent binding of Runx proteins to the *CD40* promoter in DCs. Together these data establish the *CD40* promoter as a direct target for Runx in DCs.

Next, we examined the role of each of the two Runx-binding sites (R1 and R2) in regulating mouse *CD40* promoter activity. We transfected a mouse DC cell line JAWSII, which endogenously expresses Runx1 and Runx3 (S6A Fig), with the wild-type *CD40* promoter-luciferase plasmid or with similar plasmid harboring mutations in either R1or R2 site or both R1 and R2 sites (S6B Fig). We then stimulated JAWSII cells with LPS and measured the *CD40* promoter activity via luciferase reporter assay. In response to LPS, the activity of wild-type *CD40* promoter was strongly enhanced (S6C Fig, left panel). However, mutation of R1 or R2 site alone drastically reduced LPS-stimulated *CD40* promoter activity, with a more pronounced inhibition observed when both sites were mutated (S6C Fig, left panel). We obtained similar results with TNFα stimulation (S6C Fig, right panel). Therefore, both R1 and R2 sites serve as critical determinants of *CD40* promoter activity in DCs.

**Fig 3:**
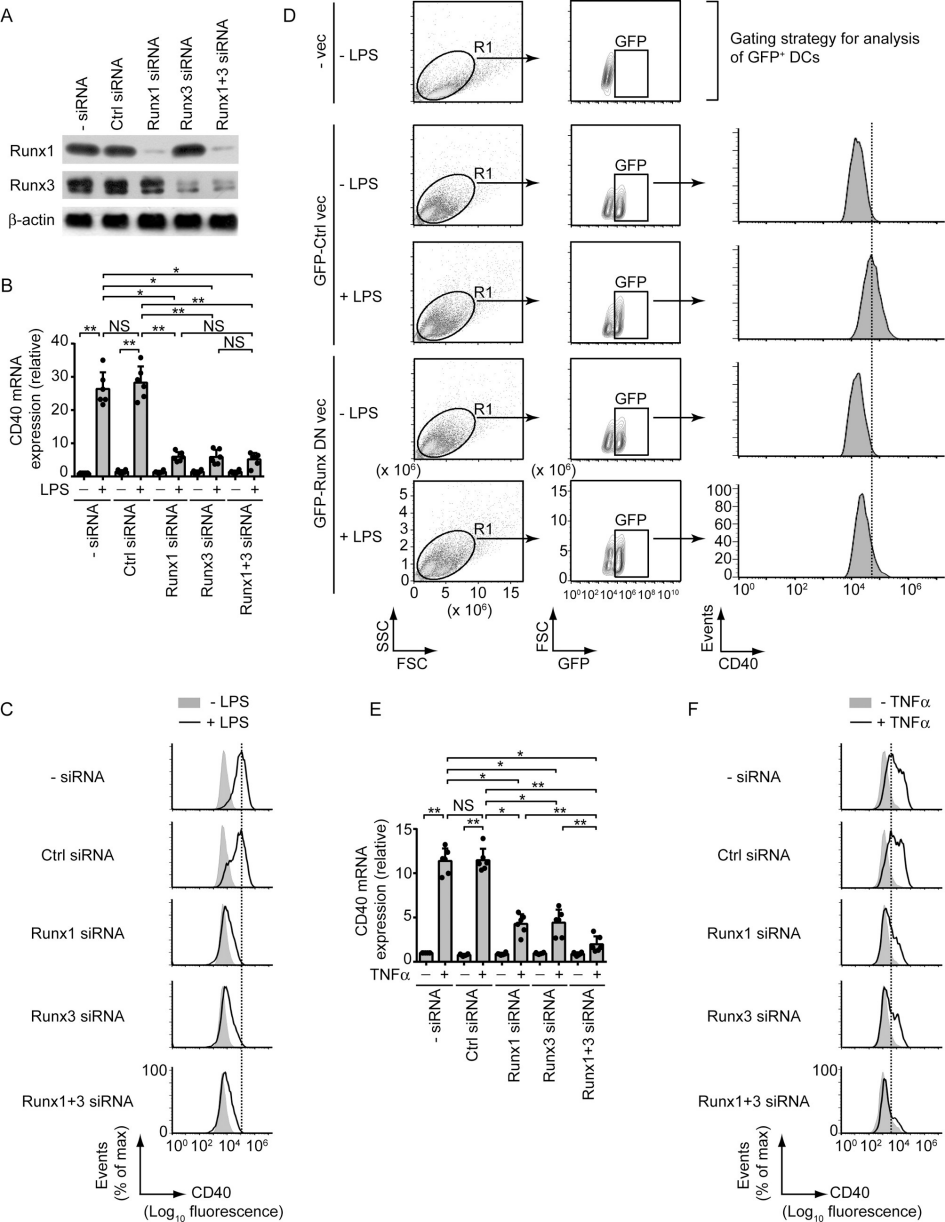
Both LPS and TNFα require Runx proteins to upregulate CD40 expression in DCs. (A) Immunoblot analysis of Runx1 and Runx3 expression in lysates of BMDCs left untransfected (- siRNA) or transfected with control (Ctrl) siRNA, Runx1 siRNA, Runx3 siRNA or Runx1 and Runx3 siRNAs (Runx1+3 siRNA). β-actin serves as a loading control. Data are representative of three independent experiments. (B) Real-time PCR quantification of CD40 mRNA in BMDCs transfected with siRNAs as in (A) and then cultured with (+) or without (-) LPS for 12 h. Results were normalized to the expression of ACTB mRNA (encoding β-actin) and are presented as fold change relative to untransfected BMDCs cultured without LPS. Data are a compilation of three independent experiments (*n* = 2 in each experiment). Each symbol represents data of individual replicate. Error bars represent SD. (C) Flow cytometry analysis of CD40 expression on BMDCs transfected with indicated siRNAs (left margin) and cultured with (+) or without (-) LPS for 24 h. Data are representative of three independent experiments. (D) BMDCs were transfected with GFP-tagged Runx dominant negative vector (Runx DN vec) or control vector (Ctrl vec) and then cultured with (+) or without (-) LPS for 24 h. CD40 expression on gated GFP^+^ DCs (i.e., transfected DC population) was analyzed via flow cytometry. Top panel [untransfected BMDCs (- vec) cultured without LPS] shows the gating strategy used for flow cytometry analysis. BMDCs were first gated based on forward and side scatter, and then further gated based on GFP expression. Data are representative of two separate experiments. (E) Real-time PCR analysis of CD40 mRNA expression in BMDCs transfected with siRNAs as in (A), then stimulated for 12 h with (+) or without (-) TNFα. Results were normalized to the expression of ACTB mRNA (encoding β-actin) and are presented as fold change relative to untransfected BMDCs cultured without TNFα. Data are a compilation of three independent experiments (*n* = 2 in each experiment). Each symbol represents data of individual replicate. Error bars represent SD. (F) Flow cytometry analysis of CD40 expression on BMDCs transfected with indicated siRNAs (left margin) and cultured with (+) or without (-) TNFα for 24 h. Data are representative of three independent experiments. For (C) and (F), the relative MFI values of CD40 expression pooled from three independent experiments are presented in S7 Fig. *p < 0.001, **p < 0.01; NS, not significant.

To determine whether Runx proteins were required for LPS- or TNFα-stimulated upregulation of CD40 expression in DCs, we silenced Runx1 and/or Runx3 expression by small interfering RNAs (siRNAs; Fig 3A). Downregulation of Runx1 or Runx3 expression highly attenuated LPS-stimulated CD40 expression at both mRNA and protein levels (Fig 3B and 3C, S7A Fig). Combined Runx1 and Runx3 silencing, however, had no added effect (Fig 3B and 3C, S7A Fig). Similar to our siRNA data, overexpression of Runx dominant negative mutant (Runx DN), which is known to block the transcriptional activity of both Runx1 and Runx3 [19], largely impaired LPS-stimulated CD40 upregulation on BMDCs (Fig 3D). The latter finding ruled out the possibility that the downregulation of LPS-stimulated CD40 expression occurred due to off-target effects of Runx1- and Runx3-specific siRNAs. Accordingly, we used these Runx1- and Runx3-specific siRNAs for subsequent experiments. We further noted that similar to LPS stimulation, TNFα-induced CD40 mRNA and protein expression was also reduced following Runx1 or Runx3 silencing and was completely blocked when Runx1 and Runx3 were silenced together (Fig 3E and 3F, S7B Fig). Thus, both Runx1 and Runx3 are required for LPS- and TNFα-driven CD40 upregulation in DCs.

### Phosphatidylinositol 3-kinase (PI3K)-Akt signaling controls Runx-mediated CD40 expression in DCs

**Fig 4:**
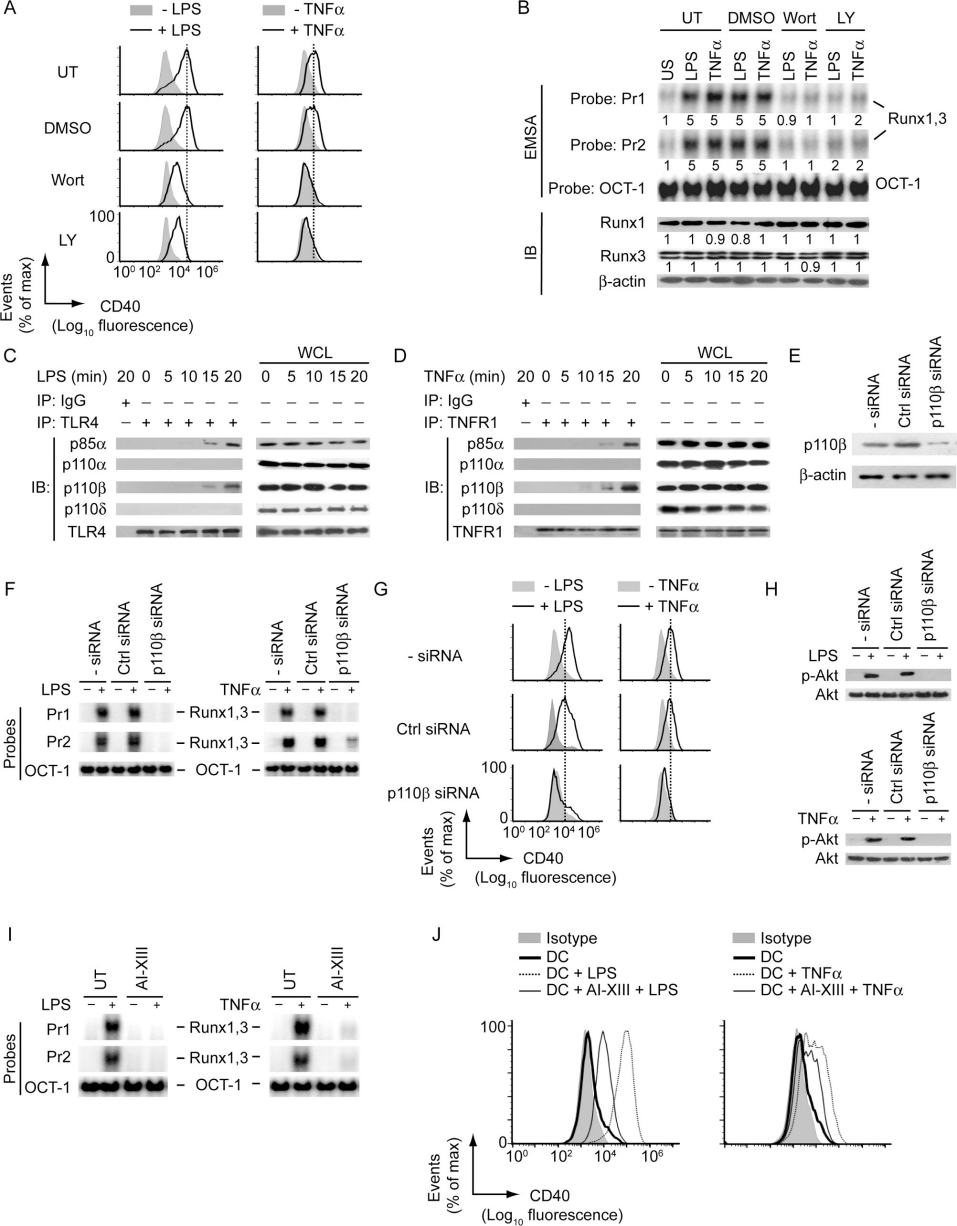
LPS and TNFα promote Runx-mediated CD40 upregulation on DCs via the PI3K-Akt pathway. (A) Flow cytometry analysis of CD40 expression on BMDCs left untreated (UT) or treated with dimethylsulfoxide (DMSO, 0.1%; control treatment), Wort or LY for 1 h and then stimulated for 24 h with (+) or without (-) LPS (left panels) or TNFα (right panels). The relative MFI data for CD40 expression pooled from three independent experiments is presented in S8A Fig. (B) BMDCs were either left untreated or treated with DMSO, Wort or LY (for 1 h) and then stimulated with LPS or TNFα for 0.5 h or left unstimulated (US). The binding of nuclear Runx1 and Runx3 to the *CD40* promoter was assessed via EMSA (probes as in Fig 2), and the expression of Runx1 and Runx3 in DC lysates was determined by immunoblot analysis. The OCT-1 binding in EMSA and β-actin expression in immunoblot analysis served as internal controls. Numbers below lanes indicate densitometry quantification of Runx1 and Runx3 binding to the *CD40* promoter (EMSA panels; normalized to OCT-1 binding), and the levels of Runx1 and Runx3 proteins [immunoblot (IB) analysis panels; normalized to β-actin]; presented relative to that of untreated BMDCs that had been left unstimulated. S8B Fig shows densitometry results pooled from three independent experiments. (C and D) Immunoprecipitation (IP; antibodies, above lanes) of lysates of BMDCs treated with LPS (C) or TNFα (D) for indicated times, followed by immunoblot analysis with antibodies to various PI3K isoforms (C and D), TLR4 (C) or TNFR1 (D). WCL, whole-cell lysate (no immunoprecipitation). (E) Immunoblot analysis of p110β/PI3K and β-actin in lysates of BMDCs left untransfected or transfected with control siRNA or PI3K p110β-specific siRNA (p110β siRNA). (F and G) EMSA (probes as in Fig 2) assessing the binding of nuclear Runx1 and Runx3 to the *CD40* promoter (F), and flow cytometry analysis measuring CD40 expression (G) in BMDCs transfected with siRNAs as in (E) and then cultured with or without LPS or TNFα for 0.5 h (F) or 24 h (G). (H) Immunoblot analysis of total and phosphorylated (p-) Akt in BMDCs transfected with indicated siRNAs, then cultured with or without LPS (upper panel) or TNFα (lower panel) for 0.3 h. (I) EMSA of nuclear extracts of BMDCs left untreated or treated with Akt inhibitor XIII (AI-XIII) for 1 h and then cultured with or without LPS or TNFα for 0.5 h, analyzed with probes as in Fig 2. (J) Analyzing (by flow cytometry) the effect of AI-XIII treatment (for 1 h) on CD40 expression by BMDCs treated with LPS (left panel) or TNFα (right panel) for 24 h. All data are representative of three independent experiments.

We then attempted to identify the upstream signaling events controlling Runx-mediated CD40 expression in DCs. In this regard, one potential regulator could be PI3K, because it associates with toll-like receptor 4 (TLR4; LPS receptor) and is required for LPS-induced CD40 expression in DCs [9,20]. The role for PI3K in TNFα-stimulated CD40 expression, however, remains unclear, although it is known to interact with the TNF receptor-1 (TNFR1) in other cell types [21]. Therefore, we initially determined the role for PI3K in LPS- and TNFα-induced upregulation of CD40 expression in DCs. While LPS and TNFα upregulated CD40 expression in untreated or control (dimethylsulfoxide; DMSO)-treated BMDCs, these effects of LPS and TNFα were substantially reduced in BMDCs treated (for 1 h) with the PI3K inhibitors wortmannin (Wort) or Ly294002 (LY) (Fig 4A, S8A Fig). Furthermore, pretreatment of BMDCs with Wort or LY inhibited LPS- or TNFα-stimulated nuclear translocation and binding of Runx proteins to the *CD40* promoter without affecting Runx protein expression (Figs 4B, S8B and S9). Thus, PI3K is necessary for promoting nuclear translocation and binding of Runx proteins to the *CD40* promoter, and subsequent upregulation of CD40 expression in DCs following LPS or TNFα treatment.

We then determined the type of PI3K complexes that were induced by LPS and TNFα, and mediated Runx binding to the *CD40* promoter. Coimmunoprecipitation analyses showed that upon LPS stimulation, TLR4 interacted with the PI3K regulatory subunit p85α, and catalytic subunit p110β but not p110α or p110δ (Fig 4C). The same PI3K complex consisting of p85α and p110β subunits also interacted with TNFR1 following TNFα stimulation (Fig 4D). Next, to assess the potential role of PI3K complex p85α-p110β in Runx-mediated regulation of CD40 expression in DCs, we silenced p110β expression by siRNA (Fig 4E). Whereas LPS or TNFα stimulated Runx binding to the *CD40* promoter and upregulated CD40 expression in untransfected and control siRNA-transfected BMDCs, these effects of LPS or TNFα were blocked in p110β-silenced BMDCs (Fig 4F and 4G). Therefore, the PI3K complex p85α-p110β is necessary for LPS- and TNFα-stimulated CD40 upregulation on DCs via Runx. Notably, the PI3K complex p85α-p110β mediated the above-mentioned effects through its downstream effector Akt. For example, silencing of PI3K p110β blocked the ability of LPS and TNFα to induce Akt phosphorylation (Figs 4H and S10). Furthermore, pretreatment of BMDCs with Akt inhibitor XIII (AI-XIII) largely inhibited LPS- and TNFα-stimulated Runx binding to the *CD40* promoter and subsequent CD40 upregulation (Fig 4I and 4J). Collectively, our results showed a critical role for the PI3K-Akt pathway in Runx-mediated CD40 upregulation on DCs.

### LD inhibits LPS- and TNFα-induced Runx binding to the *CD40* promoter by suppressing PI3K-Akt pathway

**Fig 5:**
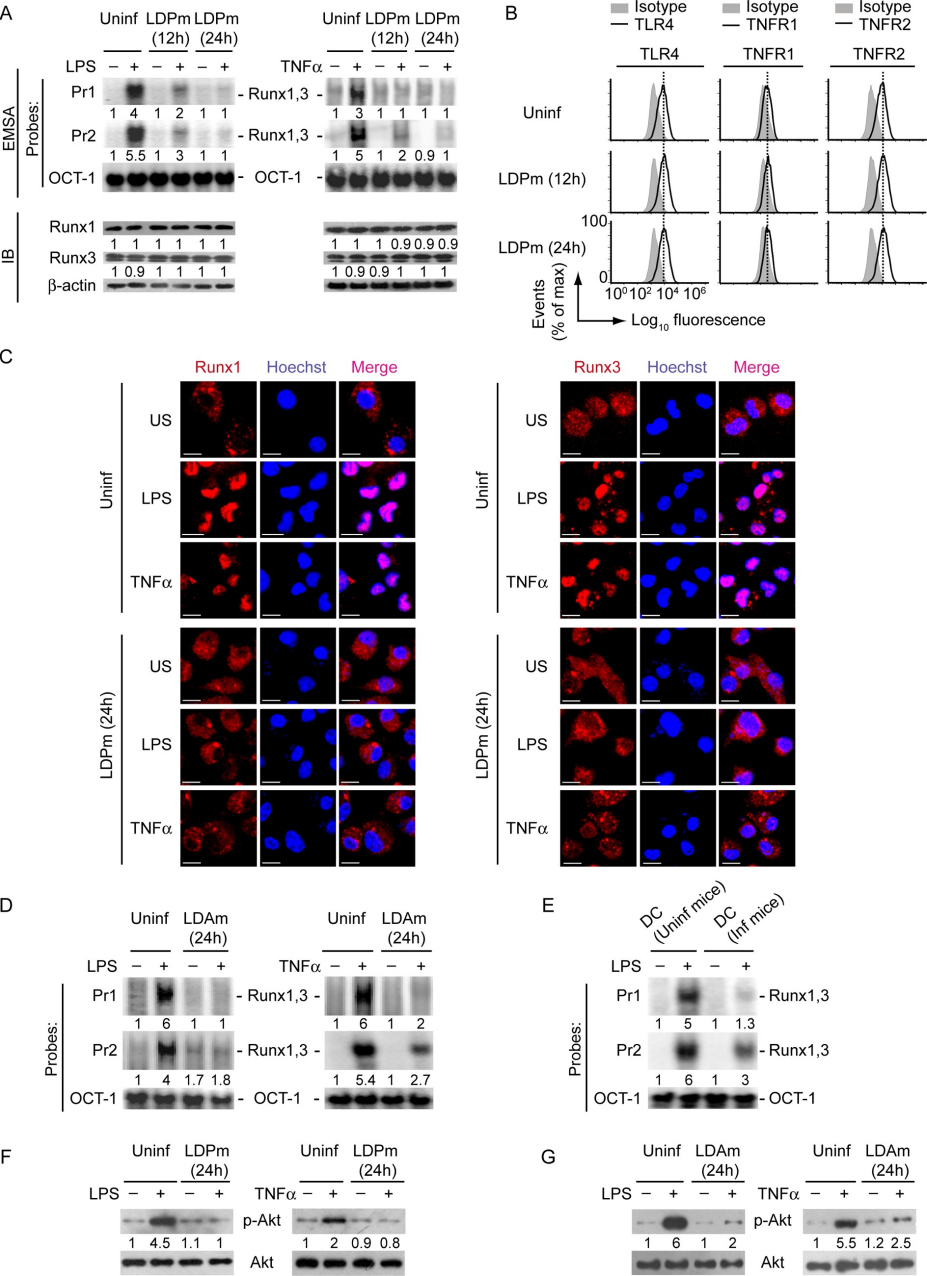
LD attenuates LPS- and TNFα-induced Runx binding to the *CD40* promoter by inhibiting PI3K-Akt pathway. (A) EMSA (probes as in Fig 2) evaluating the binding of Runx1 and Runx3 to the *CD40* promoter (top), and immunoblot analysis showing Runx1 and Runx3 expression (bottom) in BMDCs left uninfected (Uninf) or infected with LDPm for indicated times and then stimulated with (+) LPS (left panels) or TNFα (right panels) for 0.5 h or left unstimulated (-). Numbers below lanes represent densitometry (as in Fig 4B); presented relative to uninfected BMDCs that had been left unstimulated. A compilation of densitometry results from three independent experiments is shown in S11 Fig. (B) Flow cytometry analysis of TLR4, TNFR1 and TNFR2 expression on BMDCs left uninfected or infected with LDPm for indicated times. (C) Confocal microscopy of translocation of Runx1 or Runx3 (red) into the nuclei (blue; Hoechst staining) in BMDCs left uninfected or infected with LDPm for 24 h and then stimulated with LPS or TNFα for 0.5 h or left unstimulated (US). Pink color (merge) shows nuclear translocation of Runx1 or Runx3. Scale bar, 10 μm. (D and E) Nuclear lysates were prepared from BMDCs left uninfected or infected with LDAm for 24 h and then kept untreated (-) or treated (+) for 0.5 h with LPS or TNFα (D). In some experiments (E), nuclear lysates were prepared from sDCs that were derived from uninfected (Uninf mice) or LD-infected mice (Inf mice; isolated on day 45 postinfection) and then treated with LPS for 0.5 h. Lysates were subjected to EMSA (probes as in Fig 2) to detect the binding of Runx1 and Runx3 to the *CD40* promoter. Numbers below lanes indicate relative densitometry quantification of Runx1 and Runx3 binding to the *CD40* promoter (normalized to OCT-1 binding). (F and G) Immunoblot analysis of total and phosphorylated Akt in lysates of BMDCs infected with LDPm (F) or LDAm (G) for 24 h or left uninfected and then treated with LPS (left panels) or TNFα (right panels) for 0.3 h. Below lanes, densitometry, normalized to total Akt and presented relative to uninfected BMDCs given control treatment (neither LPS nor TNFα). In all experiments, LD strain AG83 was used. Data are representative of three (A, B, and D-G) or two (C) independent experiments.

Having shown that the PI3K-Akt-Runx pathway is required for LPS- and TNFα-induced CD40 upregulation, we investigated the relevance of this signaling pathway in LD-mediated suppression of CD40 expression in DCs. Initially, we determined whether LD infection influences Runx binding to the *CD40* promoter. For these experiments, we infected BMDCs with LDPm for 12 or 24 h prior to LPS or TNFα treatment, because at these time point of LD infection we observed pronounced inhibition of CD40 expression on DCs (Figs 1A and 1B, S1A and S1B). Although LPS or TNFα efficiently triggered Runx binding to the *CD40* promoter in uninfected BMDCs, these effects of LPS or TNFα were suppressed in BMDCs infected with LDPm (Figs 5A and S11, upper panels). Of note, the intracellular expression of Runx1 and Runx3, and the surface expression of TLR4, TNFR1 and TNFR2 were not affected by LD infection (Figs 5A, 5B and S11, lower panels). These results ruled out the possibility that LD inhibited Runx binding to the *CD40* promoter by downregulating the expression of Runx proteins, or TLR4 and TNF receptors. On further investigation it became clear that LDPm actually suppressed the nuclear translocation of Runx1 and Runx3 despite LPS and TNFα stimulation, which eventually resulted in less Runx binding to the *CD40* promoter in DCs (Figs 5A, 5C and S11, upper panels). Similar to LDPm, LDAm also inhibited LPS- or TNFα-stimulated binding of Runx proteins to the *CD40* promoter (Fig 5D). In addition, sDCs derived from LD-infected mice showed reduced Runx binding to the *CD40* promoter despite LPS stimulation as compared to those derived from uninfected mice (Fig 5E). These observations indicate that LD exhibits an inhibitory effect on nuclear translocation and subsequent binding of Runx proteins to the *CD40* promoter in DCs.

Because the PI3K-Akt pathway was found critical for Runx binding to the *CD40* promoter, it seemed likely that LD attenuated Runx binding to the *CD40* promoter by suppressing PI3K-Akt signaling. To confirm this possibility, we analyzed the effect of LD infection on LPS- and TNFα-induced Akt phosphorylation. We found that BMDC infection with LDPm or LDAm attenuated LPS- and TNFα-induced Akt phosphorylation (Fig 5F and 5G). Collectively, these data indicate that LD suppresses LPS- and TNFα-induced activation of the PI3K-Akt pathway and subsequent nuclear translocation and binding of Runx proteins to the *CD40* promoter, leading to the downregulation of CD40 expression on DCs.

### LD inhibits PI3K-Akt-Runx-CD40 axis via Src homology phosphotyrosine phosphatase-1 (SHP-1)

**Fig 6:**
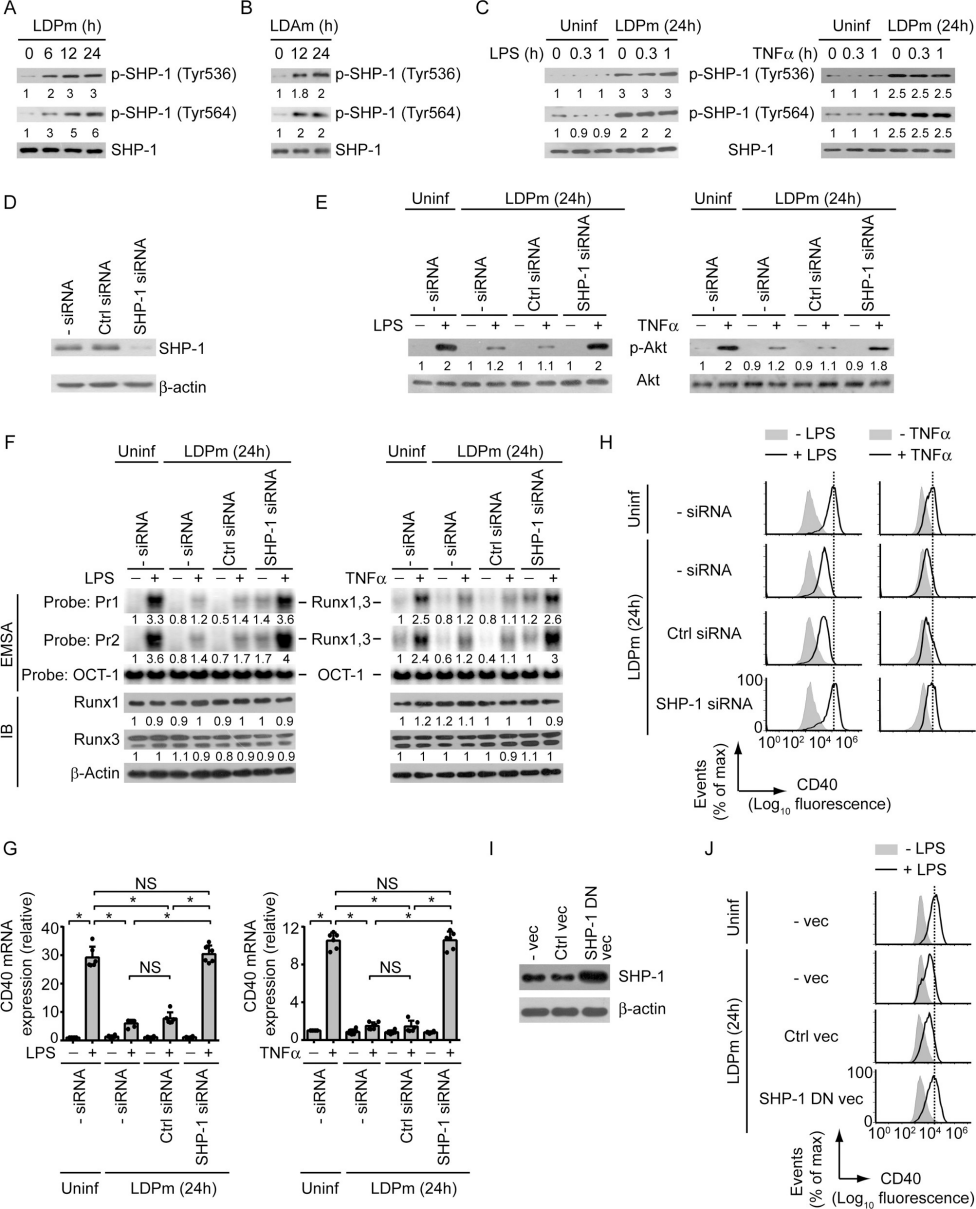
SHP-1 mediates LD-induced inhibition of CD40 expression in DCs. (A and B) Immunoblot analysis of total SHP-1 or SHP-1 phosphorylated at Tyr536 or Tyr564 in lysates of BMDCs infected for 0–24 h with LDPm (A) or LDAm (B). Numbers below lanes represent densitometry, normalized to total SHP-1 and presented relative to that of uninfected BMDCs (0 h). Data are representative of three independent experiments. (C) SHP-1 phosphorylation at Tyr536 or Tyr564, determined by immunoblot, in BMDCs left uninfected or infected with LDPm for 24 h and then treated with LPS (left panels) or TNFα (right panels) for indicated times. The expression of total SHP-1 serves as a loading control. Below lanes, densitometry results as in (A), presented relative to uninfected BMDCs treated with LPS or TNFα for 0 h. Data are representative of three independent experiments. (D) BMDCs were left untransfected or transfected with control siRNA or SHP-1-specific siRNA. Cell lysates were immunoblotted for SHP-1 or β-actin. Data are representative of three independent experiments. (E and F) Analysis of Akt phosphorylation (E; determined by immunoblot analysis), the binding of nuclear Runx1 and Runx3 to the *CD40* promoter (F; assessed by EMSA using probes as in Fig 2) and the expression of Runx1 and Runx3 (F; analyzed by immunoblot analysis) in BMDCs transfected with indicated siRNAs and then infected with LDPm for 24 h or left uninfected, and treated with or without LPS (left panels) or TNFα (right panels) for 0.3 h (E) or 0.5 h (F). Below lanes, densitometry (as in Figs 4B and 5F), presented relative to control BMDCs (BMDCs left untransfected and uninfected, and given no LPS or TNFα treatment). Data are representative of three independent experiments. (G) Real-time PCR analysis of CD40 mRNA expression in BMDCs transfected with indicated siRNAs and then infected with LDPm for 24 h and cultured with or without LPS (left panel) or TNFα (right panel) for 12 h. Results were normalized to the expression of ACTB mRNA (encoding β-actin) and are presented as fold change relative to control BMDCs (BMDCs left untransfected and uninfected, and cultured without LPS or TNFα). Data are a compilation of three independent experiments (*n* = 2 in each experiment). Each symbol represents data of individual replicate. Error bars represent SD. (H) Flow cytometry analysis of CD40 expression by BMDCs transfected with indicated siRNAs, then infected with LDPm for 24 h and stimulated with LPS (left panels) or TNFα (right panels) for 24 h. Data are representative of three independent experiments. (I) Immunoblot analysis of SHP-1 and β-actin (loading control) in BMDCs left untransfected (- vec) or transfected with control vector (Ctrl vec) or SHP-1 DN vector (SHP-1 DN vec). Data are representative of two independent experiments. (J) Flow cytometry analysis of CD40 expression by BMDCs transfected as in (I), then infected with LDPm for 24 h and cultured with (+) or without (-) LPS for 24 h. Data are representative of two independent experiments. In all experiments, LD strain AG83 was used. The pooled densitometry results for (A) and (F), and the relative MFI data of CD40 expression for (H) from three independent experiments are shown in S12 Fig. *p < 0.001; NS, not significant.

Next, we examined the mechanism by which LD suppressed PI3K-Akt-Runx-CD40 axis in DCs. Previous reports showed that the PI3K-Akt signaling is negatively regulated by SHP-1 and that the activity of SHP-1 is induced in macrophages following LD infection [22,23]. Therefore, we wondered whether SHP-1 was involved in LD-mediated suppression of CD40 expression in DCs. Notably, the role of SHP-1 in the regulation of Runx activity and CD40 expression remains unknown. Initially, we determined whether LD induces SHP-1 activation in DCs. Kinetic analyses showed that SHP-1 activation, as measured by phosphorylation of SHP-1 at Tyr536 and Tyr564 [24,25], was augmented at 6 h after LDPm infection and continued up to 24 h postinfection (Figs 6A and S12A). Like LDPm, LDAm also triggered SHP-1 activation in DCs (Fig 6B). This SHP-1 induction by LD, however, was not affected by LPS or TNFα treatment (Fig 6C). We further found that silencing of SHP-1 by siRNA blocked the inhibitory effect of LDPm on LPS- and TNFα-induced Akt phosphorylation, binding of Runx proteins to the *CD40* promoter, and upregulation of CD40 mRNA and protein expression by DCs (Figs 6D–6H, S12B and S12C). However, SHP-1 silencing did not affect the intracellular expression of Runx1 and Runx3, and the surface expression of TLR4, TNFR1 and TNFR2 (Figs 6F, S12B and S13). Therefore, silencing of SHP-1 restored LPS- or TNFα-mediated CD40 upregulation on LD-infected DCs by reinstating Runx binding to the *CD40* promoter and not by enhancing the expression of Runx proteins, or TLR4 and TNF receptors. Similar to SHP-1 silencing by siRNA, overexpression of catalytically inactive SHP-1 dominant negative mutant (SHP-1 DN; SHP-1 carrying mutation at cysteine 453 (C453S); [26]) also prevented LD-induced inhibition of LPS-stimulated CD40 expression in DCs (Fig 6I and 6J). This data negates the possibility that the blockade of LD-induced inhibition of CD40 expression upon SHP-1 silencing occurred due to off-target effect of SHP-1-specific siRNA. Our findings thus revealed a previously unidentified inhibitory role for SHP-1 in the regulation of CD40 expression. Together these results indicate that LD inhibits LPS- and TNFα-stimulated CD40 upregulation on DCs by suppressing the PI3K-Akt-Runx pathway through SHP-1.

### Sodium antimony gluconate (SAG)-induced DC antileishmanial response relies on Runx-mediated CD40 upregulation

**Fig 7:**
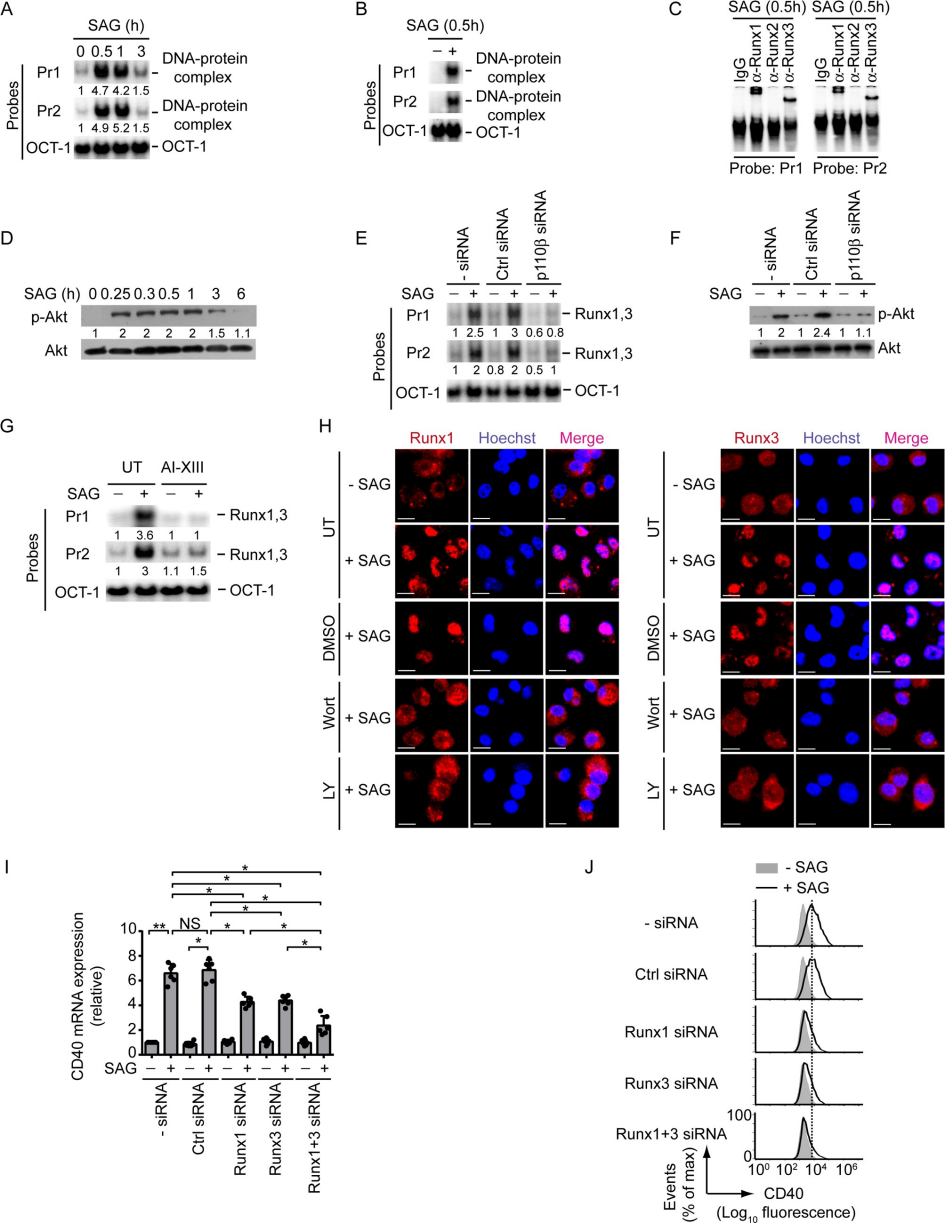
SAG induces CD40 upregulation on DCs via the PI3K-Akt-Runx pathway. (A and B) EMSA of nuclear extracts of BMDCs (A) or sDCs (B) treated with SAG for indicated times, assessed with probes as in Fig 2. Numbers below lanes in (A) represent densitometry (normalized to OCT-1 binding), presented relative to untreated BMDCs (0 h). Data are representative of three independent experiments. (C) Supershift EMSA (antibodies and probes are indicated above and below lanes, respectively) analyzing the binding of nuclear Runx1, Runx2 and Runx3 to the *CD40* promoter in BMDCs treated with SAG for 0.5 h. Data are representative of three independent experiments. (D) Immunoblot analysis of total and phosphorylated Akt in lysates of BMDCs treated with SAG for indicated times. Numbers below lanes represent densitometry, normalized to total Akt and presented relative to untreated BMDCs (0 h). Data are representative of two independent experiments. (E) EMSA of nuclear extracts of BMDCs left untransfected or transfected with control siRNA or PI3K p110β-specific siRNA (p110β siRNA) and then treated with or without SAG for 0.5 h, assessed with probes as in Fig 2. Below lanes, densitometry as in (A); presented relative to untransfected BMDCs not treated with SAG. Data are representative of three independent experiments. (F) Immunoblot analysis of total and phosphorylated Akt in lysates of BMDCs transfected with siRNAs as in (E) and then treated with SAG for 0.3 h. Below lanes, densitometry (as in Fig 5F), presented relative to untransfected BMDCs given no SAG treatment. Data are representative of three independent experiments. (G) EMSA (probes as in Fig 2) of nuclear extracts of BMDCs left untreated or treated with AI-XIII for 1 h and then cultured with or without SAG for 0.5 h. Below lanes, densitometry as in (A), presented relative to untreated BMDCs cultured without SAG. Data are representative of three independent experiments. (H) Confocal microscopy of the translocation of Runx1 or Runx3 (red) into the nuclei (blue; Hoechst staining) in BMDCs left untreated (UT) or treated with DMSO, Wort or LY for 1 h, then cultured with or without SAG for 0.5 h. Pink color (merge) shows nuclear translocation of Runx1 or Runx3. Scale bar, 10 μm. Data are representative of two independent experiments. (I) Real-time PCR analysis of CD40 mRNA expression in BMDCs left untransfected (- siRNA) or transfected with control siRNA or Runx1- and/or Runx3-specific siRNA, then cultured for 12 h with or without SAG. Results were normalized to the expression of ACTB mRNA (encoding β-actin) and are presented as fold change relative to untransfected BMDCs cultured without SAG. Data are compilation of three independent experiments (*n* = 2 in each experiment). Each symbol represents data of individual replicate. Error bars represent SD. (J) Analyzing (by flow cytometry) the effect of Runx1 and/or Runx3 silencing on CD40 expression by BMDCs treated for 24 h with or without SAG. Data are representative of three independent experiments. The densitometry results for (A), and the relative MFI of CD40 expression for (J) pooled from three independent experiments are shown in S14 Fig. *p < 0.001, **p < 0.01; NS, not significant.

**Fig 8:**
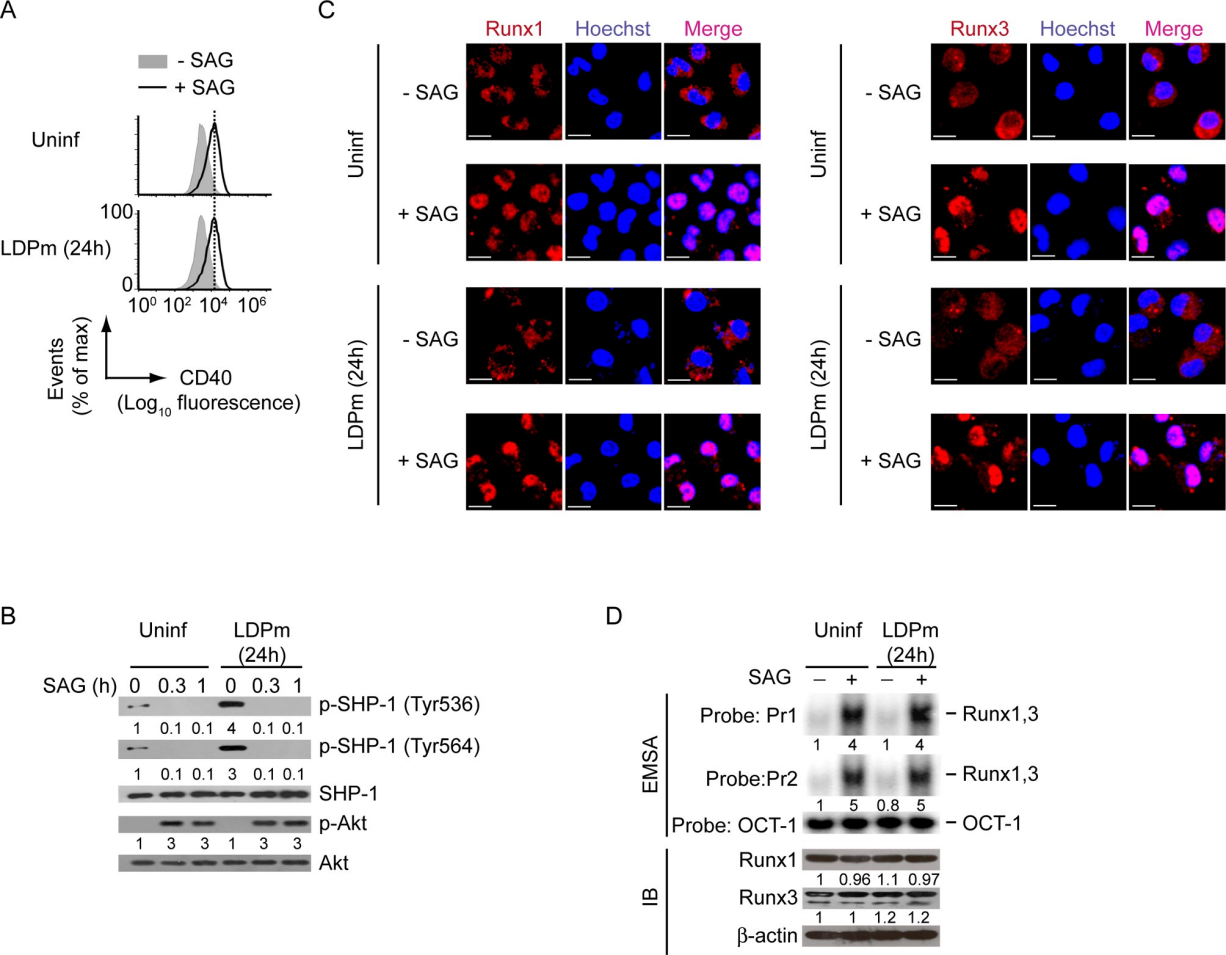
SAG-mediated SHP-1 inhibition allows PI3K-Akt-Runx signaling to upregulate CD40 expression on LD-infected DCs. (A) CD40 expression profile on BMDCs left uninfected or infected with LDPm for 24 h, then treated with or without SAG for 24 h, assessed by flow cytometry. Data are representative of three independent experiments. (B) Immunoblot analysis of total and phosphorylated SHP-1 and Akt in lysates of BMDCs infected with LDPm for 24 h or left uninfected and then treated with SAG for indicated times. Number below lanes indicate densitometry of phosphorylated SHP-1 (normalized to total SHP-1) or phosphorylated Akt (normalized to total Akt), presented relative to uninfected BMDCs treated with SAG for 0 h. Data are representative of three independent experiments. (C) Confocal microscopy of the translocation of Runx1 or Runx3 (red) into the nuclei (blue; Hoechst staining) in BMDCs left uninfected or infected with LDPm for 24 h, then cultured with or without SAG for 0.5 h. Pink color (merge) shows nuclear translocation of Runx1 or Runx3. Scale bar, 10 μm. Data are representative of two independent experiments. (D) EMSA (with probes as in Fig 2) assessing the binding of nuclear Runx1 and Runx3 to the *CD40* promoter (upper panel) and immunoblot analysis depicting Runx1 and Runx3 expression (lower panel) in BMDCs left uninfected or infected with LDPm for 24 h, then cultured for 0.5 h with or without SAG. Numbers below lanes indicate densitometry quantification of Runx1 and Runx3 binding to the *CD40* promoter (EMSA panels; normalized to OCT-1 binding), and the levels of Runx1 and Runx3 proteins [immunoblot (IB) analysis panels; normalized to β-actin]; presented relative to uninfected BMDCs cultured without SAG. Data are representative of three independent experiments. For all experiments LD strain AG83 was used. The relative MFI of CD40 expression for (A) and the densitometry results for (D) pooled from three independent experiments are shown in S15 Fig.

Given that Runx proteins are necessary for CD40 upregulation on DCs (Fig 3) and that CD40 expressed by DCs contributes to host protection [7], we speculated a role for Runx in regulating antileishmanial immune responses mediated by DCs. To test this hypothesis, we focused on SAG (the first-line drug against VL [27]) because it upregulates CD40 expression on DCs [28]. Initially, we evaluated the role of Runx in SAG-induced CD40 expression in DCs. EMSA and supershift analyses showed that the treatment of BMDCs and sDCs with SAG led to increased Runx1 and Runx3 binding to the *CD40* promoter (Figs 7A–7C and S14A). Previously, we have shown that SAG activates PI3K-Akt signaling in DCs [28]. In fact, phosphorylation of Akt was triggered within 0.25 h after SAG treatment, remained sustained up to1 h, and then started decreasing at 3 h (Fig 7D). Our data here further suggest that SAG requires the PI3K complex p85α-p110β and its downstream effector Akt to induce Runx binding to the *CD40* promoter (Fig 7E–7G). Indeed, silencing of PI3K p110β by siRNA or pretreatment with Akt inhibitor AI-XIII substantially reduced the binding of Runx proteins to the *CD40* promoter despite SAG treatment (Fig 7E and 7G). The PI3K-Akt pathway promoted SAG-induced binding of Runx proteins to the *CD40* promoter by enhancing their (i.e., Runx1 and Runx3) nuclear translocation. For example, BMDC treatment with SAG considerably increased the nuclear translocation of Runx1 and Runx3 proteins (Fig 7H). This SAG-induced effect, however, was inhibited when we pretreated BMDCs with PI3K inhibitors Wort or LY (Fig 7H). Further analyses depicted that silencing of Runx1 or Runx3 decreased SAG-stimulated CD40 mRNA and protein expression in BMDCs. In addition, combined silencing of Runx1 and Runx3 robustly inhibited the upregulation of CD40 mRNA and protein expression induced by SAG (Figs 7I, 7J and S14B). Interestingly, SAG, unlike LPS or TNFα (Fig 1), upregulated CD40 expression on DCs even after LD infection (Figs 8A and S15A). This finding correlates with the observations that SAG effectively blocked LD-induced SHP-1 activation (Fig 8B), whereas LPS and TNFα did not (Fig 6C). Consequently, SAG continued to drive Akt phosphorylation (Fig 8B), which in turn augmented the nuclear translocation and the binding of Runx proteins to the *CD40* promoter (Figs 8C and Fig 8D; S15B, left two panels) and thereby increased CD40 expression on BMDCs despite LD infection (Figs 8A and S15A). However, neither SAG treatment nor LD infection had any effect on Runx1 and Runx3 expression in BMDCs (Figs 8D and S15B, right two panels). In addition, we did not detect any significant change in both percentage of LD-infected DCs and intracellular parasite number following SAG treatment for 0.3 h or 1 h (S16 Fig). The latter finding indicated that the inhibition of LD-induced SHP1 activation, which occurred as early as 0.3 h or 1 h after SAG treatment (Fig 8B), was not an outcome of SAG-mediated clearance of intracellular LD parasites. Instead, SAG, being a potent inhibitor of SHP-1 [29–36], directly inhibited LD-induced SHP-1 activation at the above-mentioned time points. This conclusion is supported by a report [29] demonstrating that SAG forms stable complexes with SHP-1 and that SAG directly targets the catalytic domain of SHP-1 to inhibit its activity. Although our results (Figs 8B and S16) showed a direct inhibitory effect of SAG on LD-induced SHP-1 activation at early time points, a prolonged SAG exposure might be needed to continuously suppress LD-induced SHP-1 activation. In that case, the inhibition of LD-induced SHP-1 activation may partly be contributed by SAG-mediated reduction of intracellular parasite load. Nevertheless, our findings demonstrated that SAG promotes CD40 upregulation on LD-infected DCs via Runx by directly or indirectly inhibiting LD-induced SHP-1 activation.

**Fig 9:**
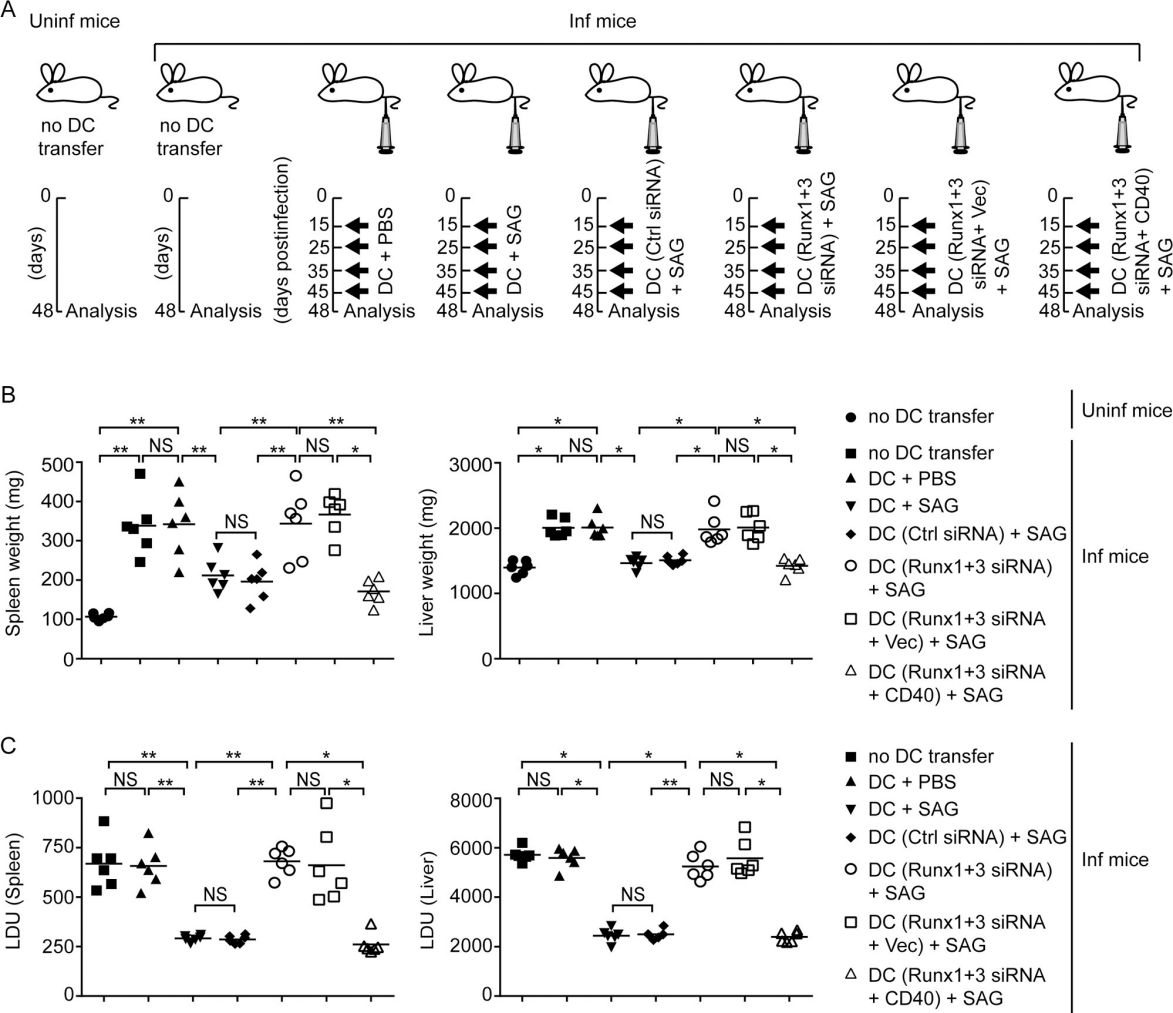
Runx-mediated CD40 expression is required for clearance of LD infection in mice by SAG-treated DCs. (A) Experimental scheme for adoptive transfer experiments. BALB/c BMDCs were treated with PBS or SAG for 24 h. Alternatively, BALB/c BMDCs were transfected with control siRNA, or with Runx1 and Runx3 siRNAs alone or together with an empty vector (Vec) or CD40-expressing vector (CD40) prior to SAG treatment (see S17 Fig showing the level of CD40 expression on these DCs). After such treatment, DCs were transferred i.v. into LD-infected (Inf) BALB/c mice on days 15, 25, 35 and 45 postinfection. In some sets of the experiment, age-matched uninfected (Uninf) mice and LD-infected BALB/c mice were left without any DC transfer (no DC transfer). On day 48 postinfection, spleens and livers were isolated for analysis. (B and C) Spleen and liver weights (B), and splenic and liver parasite burden [measured by stamp-smear method and presented as Leishman-Donovan Unit (LDU); (C)] in mice treated as in (A); assessed on day 48 postinfection. Each symbol represents an individual mouse. Data are compilation of two separate experiments (*n* = 3 mice per group in each experiment). The horizontal bars represent the mean. The representative pictures of spleens and livers from these mice are shown in S18 Fig. In all experiments, LD strain AG83 was used.*p < 0.001, **p < 0.01; NS, not significant.

**Fig 10:**
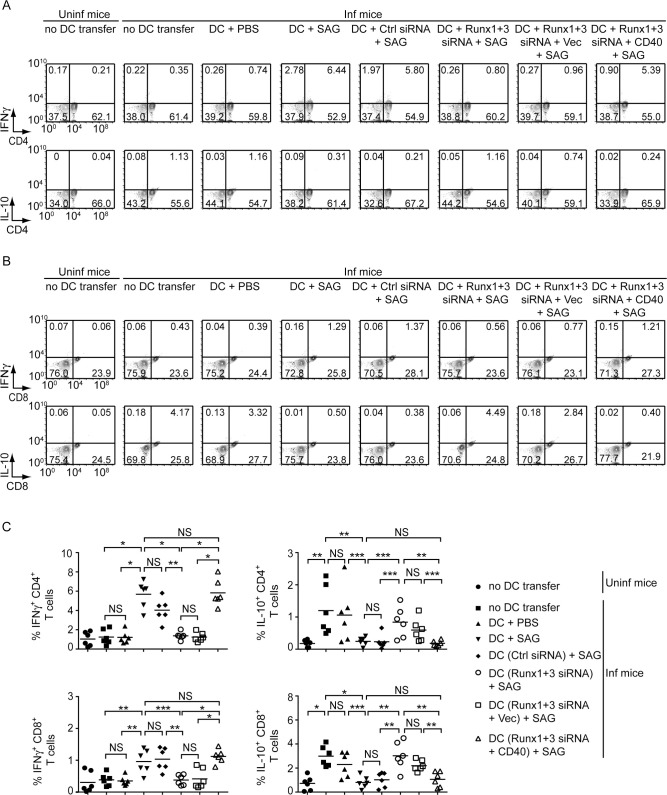
SAG-induced Runx-dependent CD40 upregulation on DCs promotes type-1 T cell response in LD-infected mice. Runx1- and Runx3-silenced BMDCs (with or without CD40 overexpression), and control-silenced BMDCs were treated with SAG and then adoptively transferred into LD-infected mice. Alternatively, age-matched uninfected and LD-infected mice were left without any DC transfer (details given in Fig 9 legend). (A and B) Frequency of IFNγ- and IL-10-producing CD4^+^ (A) or CD8^+^ (B) T cells in splenocytes isolated from these mice, assessed by flow cytometry (representative data of *n* = 6). Numbers indicate the percentage of cells in the respective quadrants. Gating strategy used for flow cytometry analysis is described in S19 Fig. (C) Graphs displaying cumulative data of the percentage of IFNγ- and IL-10-producing CD4^+^ (upper panels) or CD8^+^ (lower panels) T cells from two independent experiments (*n* = 3 mice per group in each experiment). Each symbol represents an individual mouse; horizontal bars represent the mean. In all experiments, LD strain AG83 was used. *p < 0.001, **p < 0.01, ***p < 0.05; NS, not significant.

Next, we tested whether Runx proteins, by promoting CD40 expression, contributed to SAG-induced antileishmanial response of DCs *in vivo*. To assess this, we silenced (or not) Runx1 and Runx3 expression in DCs and then treated with SAG. In some cases, we overexpressed CD40 in Runx1- and Runx3-silenced DCs prior to SAG treatment (S17 Fig). Afterward, we adoptively transferred these DCs into LD-infected syngeneic mice on the days 15, 25, 35 and 45 postinfection. On day 48 postinfection, we analyzed liver and spleen weights, splenic and liver parasite load, and the frequencies of splenic CD4^+^ and CD8^+^ T cells producing IFNγ or IL-10 (Fig 9A–9C and Fig 10). Upon transferring SAG-treated DCs, but not PBS-treated DCs, the liver and spleen weights and parasite load were drastically reduced in LD-infected mice. These effects of SAG-treated DCs were largely compromised after Runx1 and Runx3 silencing. However, forced CD40 expression in SAG-treated Runx1- and Runx3-silenced DCs improved the efficacy of these cells to reduce liver and spleen weights and parasite burden in LD-infected mice (Figs 9B, 9C and S18). We further observed that upon transfer of SAG-treated DCs, but not PBS-treated DCs, the frequencies of IFNγ-producing CD4^+^ and CD8^+^ T cells were increased and IL-10-producing CD4^+^ and CD8^+^ T were reduced in spleens of LD-infected mice. Silencing of Runx1 and Runx3 in SAG-treated DCs, however, reversed these effects. Strikingly, forced CD40 expression restored the ability of SAG-stimulated Runx1- and Runx3-silenced DCs to increase IFNγ-producing and reduce IL-10-producing CD4^+^ or CD8^+^ T cell populations to an extent similar to that observed in case of SAG-treated DCs (Figs 10 and S19). Collectively, these data suggest that Runx proteins play critical roles in mediating SAG-induced antileishmanial response of DCs by promoting CD40 expression and thereby augmenting type-1 T cell immunity.

### Persistent SHP-1 activation by antimony-resistant LD impairs SAG-induced CD40 upregulation on DCs

**Fig 11:**
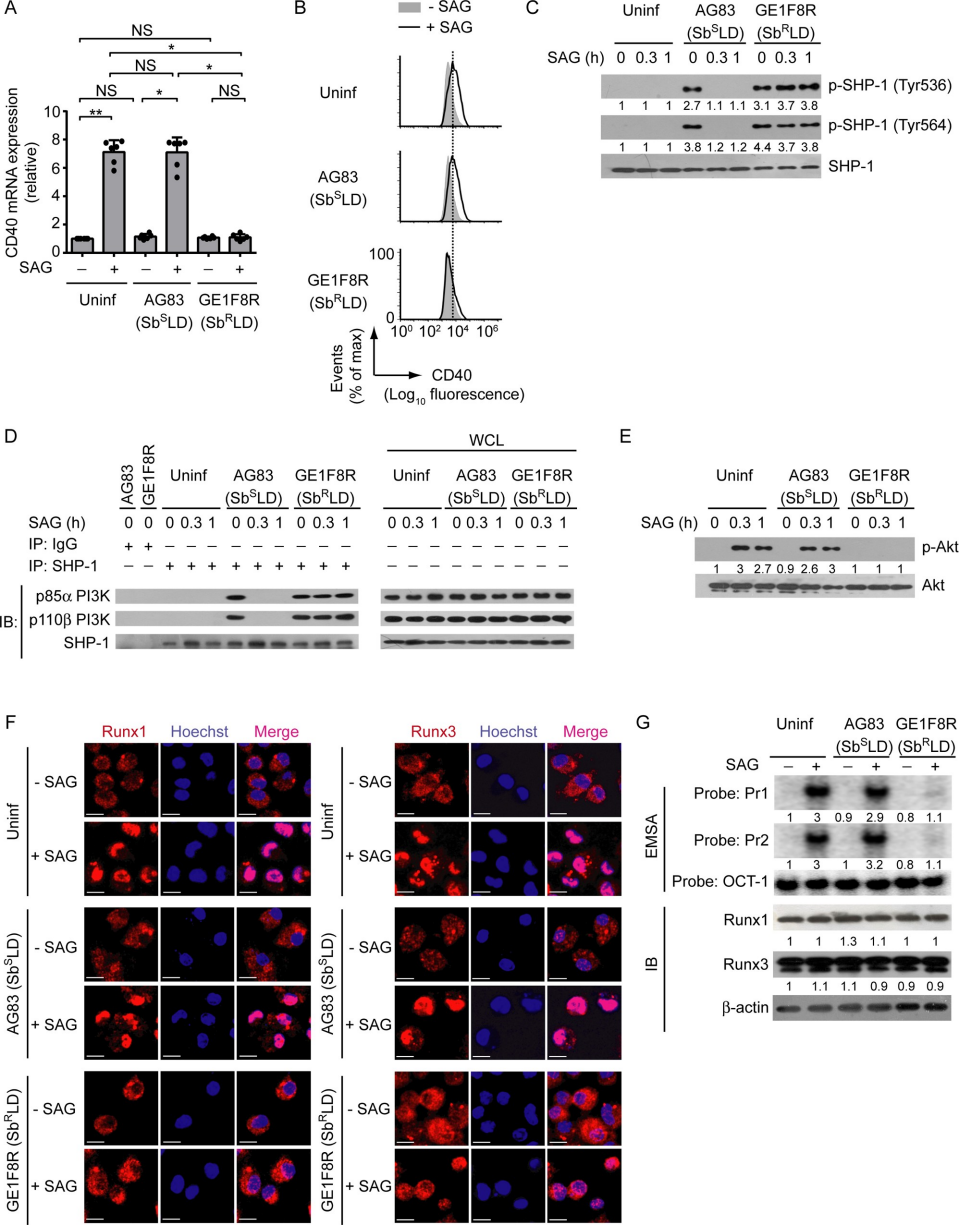
Sb^R^LD prevents SAG-induced Runx-dependent CD40 upregulation on DCs by persistently activating SHP-1. (A) Real-time PCR analysis of CD40 mRNA expression in BMDCs left uninfected or infected for 24 h with promastigotes of Sb^R^LD strain GE1F8R or Sb^S^LD strain AG83, then cultured with or without SAG for 12 h. Results were normalized to the expression of ACTB mRNA (encoding β-actin) and presented as fold change relative to uninfected BMDCs cultured without SAG. Data are a compilation of three independent experiments (*n* = 2 in each experiment). Each symbol represents data of individual replicate. Error bars represent SD. (B) Flow cytometry analysis of CD40 expression on BMDCs infected as in (A) and then cultured with or without SAG for 24 h. Data are representative of three independent experiments. (C) Immunoblot analysis of total and phosphorylated SHP-1 in lysates of BMDCs left uninfected or infected with promastigotes of Sb^R^LD strain GE1F8R or Sb^S^LD strain AG83 for 24 h and then treated with SAG for indicated times. Number below lanes indicate densitometry of phosphorylated SHP-1 (normalized to total SHP-1), presented relative to uninfected BMDCs treated with SAG for 0 h. Data are representative of three independent experiments. (D) Immunoprecipitation (IP) and immunoblot (IB) analysis of the interaction of SHP-1 with the PI3K complex p85α-p110β in BMDCs infected with promastigotes of Sb^R^LD strain GE1F8R or Sb^S^LD strain AG83 for 24 h and then treated with SAG for indicated times. IgG represents immunoglobulin G (IP control), and WCL is whole-cell lysate (no IP). Data are representative of two independent experiments. (E) Immunoblot analysis of total and phosphorylated Akt in lysates of BMDCs left uninfected or infected with promastigotes of Sb^R^LD strain GE1F8R or Sb^S^LD strain AG83 for 24 h and then treated with SAG for indicated times. Number below lanes indicate densitometry of phosphorylated Akt (normalized to total Akt), presented relative to uninfected BMDCs treated with SAG for 0 h. Data are representative of three independent experiments. (F) Confocal microscopy of the translocation of Runx1 or Runx3 (red) into the nuclei (blue; Hoechst staining) in BMDCs left uninfected or infected with promastigotes of Sb^R^LD strain GE1F8R or Sb^S^LD strain AG83 for 24 h and then treated with (+) or without (-) SAG for 0.5 h. Pink color (merge) shows nuclear translocation of Runx1 or Runx3. Scale bar, 10 μm. Data are representative of two independent experiments. (G) EMSA (with probes as in Fig 2) of the binding of nuclear Runx1 and Runx3 to the *CD40* promoter (upper panel), and immunoblot analysis of Runx1 and Runx3 expression (lower panel) in BMDCs left uninfected or infected with promastigotes of Sb^R^LD strain GE1F8R or Sb^S^LD strain AG83 for 24 h and then cultured for 0.5 h with or without SAG. Below lane, densitometry (as in Fig 8D); presented relative to uninfected BMDCs cultured without SAG. Data are representative of three independent experiments. The relative MFI data of CD40 expression for (B), and densitometry result for (G) pooled from three independent experiments are shown in S20 Fig. *p < 0.001, **p < 0.01; NS, not significant.

Up to now, we demonstrated the ability of LD to regulate CD40 expression on DCs using normal LD [i.e., antimony-sensitive LD (Sb^S^LD)] strain AG83, and lastly showed that this Sb^S^LD strain failed to inhibit SAG-induced CD40 expression on DCs (Fig 8A). In contrast, our previous study [28], coupled with the data presented here (Figs 11A, 11B and S20A), showed that the antimony-resistant LD (Sb^R^LD) strain GE1F8R effectively inhibited SAG-stimulated CD40 expression by DCs at both mRNA and protein levels. Since LD required SHP-1 to regulate PI3K-Akt-Runx pathway-mediated CD40 expression in DCs (Fig 6), we determined the involvement of these signaling elements in the modulation of CD40 expression by Sb^R^LD. Initially, we compared the ability of Sb^R^LD and Sb^S^LD to induce SHP-1 activation (measured by SHP-1 phosphorylation at Tyr536 and Tyr564 as described above) in DCs following SAG treatment. While Sb^S^LD strain AG83 could not induce SHP-1 phosphorylation in the presence of SAG, Sb^R^LD strain GE1F8R continued to drive SHP-1 phosphorylation in BMDCs despite SAG treatment (Fig 11C). A previous study [22] has reported that SHP-1, upon phosphorylation, prevents the activation of PI3K-Akt pathway by physically interacting with the p85α subunit of PI3K complex. Consistent with this report, our results showed that in response to both AG83 (Sb^S^LD) and GE1F8R (Sb^R^LD) infection, SHP-1 physically interacted with the PI3K complex p85α-p110β (Fig 11D). The interaction between SHP-1 and the PI3K complex p85α-p110β, however, was prevented in AG83 (Sb^S^LD)-infected DCs upon SAG treatment (Fig 11D). On the contrary, SHP-1 constantly interacted with the PI3K complex p85α-p110β in GE1F8R (Sb^R^LD)-infected DCs despite SAG treatment (Fig 11D). Consequently, SAG-induced Akt phosphorylation (Fig 11E), the nuclear translocation and the binding of Runx proteins to the *CD40* promoter (Figs 11F, 11G and S20B, left two panels), and the upregulation of CD40 mRNA and protein expression (Figs 11A, 11B and S20A) were largely impaired in GE1F8R (Sb^R^LD)-infected BMDCs. In contrast, these SAG-induced events were readily detected in BMDCs infected with AG83 (Sb^S^LD) (Figs 11A, 11B, 11E–11G, S20A and S20B). Notably, the intracellular expression of Runx1 and Runx3 remained unchanged despite Sb^R^LD or Sb^S^LD infection or SAG treatment (Figs 11G and S20B, right two panels). Thus, Sb^R^LD, but not Sb^S^LD, suppresses SAG-induced PI3K-Akt-Runx signaling and subsequent CD40 upregulation on DCs by persistently activating SHP-1.

**Fig 12:**
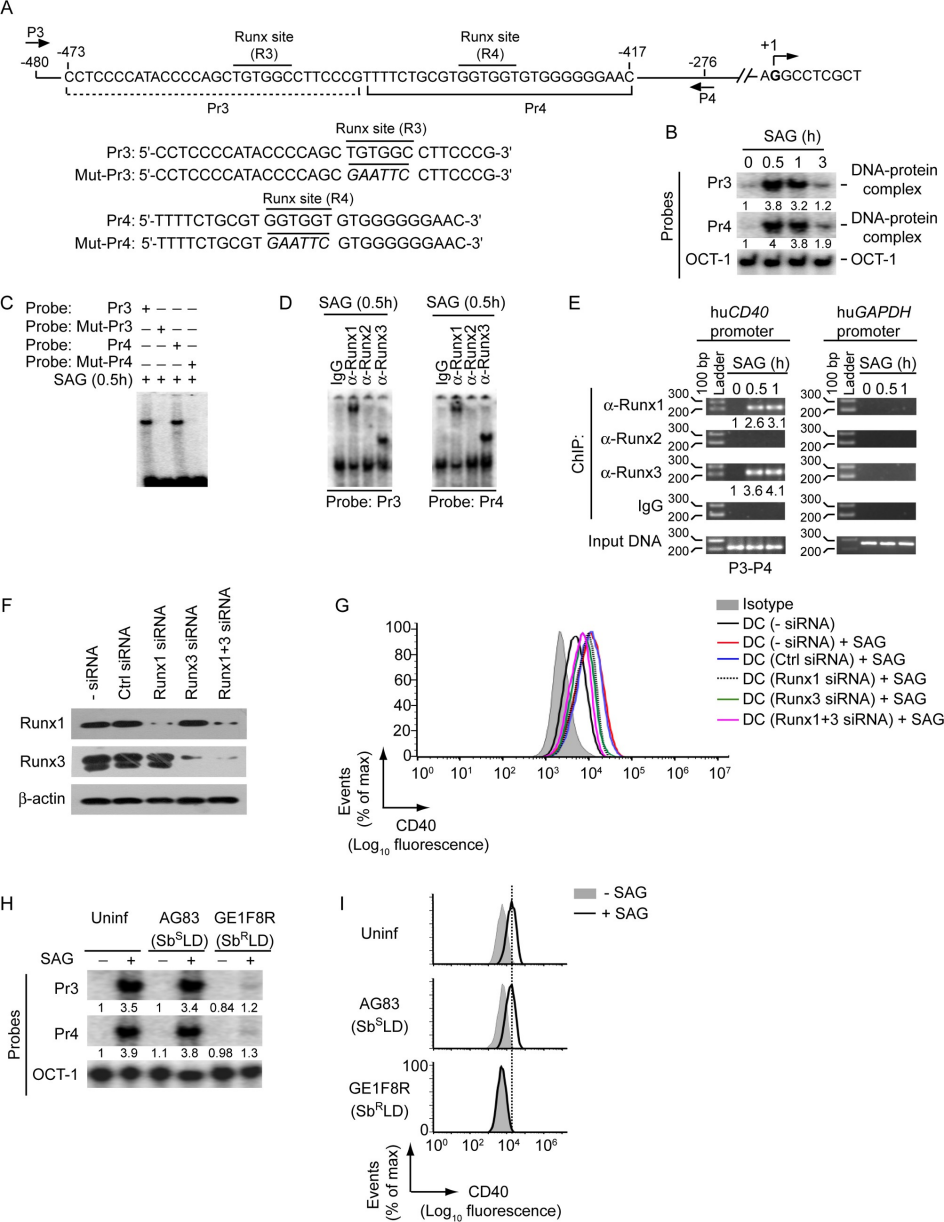
Sb^R^LD inhibits SAG-induced CD40 upregulation on HuMoDCs by impairing Runx binding to the *CD40* promoter. (A) A schematic of human *CD40* promoter showing the location of putative Runx-binding sites (R3 and R4) and ChIP primers (P3 and P4), and details of oligonucleotide probes used for EMSA. Human *CD40* promoter-specific Pr3 and Pr4 probes contain putative wild-type Runx-binding sites, and Mut-Pr3 and Mut-Pr4 probes contain mutations (in italics) at Runx-binding sites. Base positions are relative to the transcription start site. (B and C) EMSA of nuclear extracts of HuMoDCs treated with SAG for indicated times; assayed with indicated probes. OCT-1 binding (in B) serves as an internal control. Numbers below lanes in (B), densitometry readings (as in Fig 2B); presented relative to untreated HuMoDCs (0 h). (D) Supershift EMSA (antibodies and probes are indicated above and below lanes, respectively) to assess the binding of nuclear Runx1, Runx2 and Runx3 to the *CD40* promoter in HuMoDCs treated with SAG for 0.5 h. (E) ChIP assay [with the primers shown in (A) and antibodies indicated at left margin] to determine the binding of Runx proteins to -480/-276 region of the *CD40* promoter in HuMoDCs treated with SAG (time, above lanes). Amplification of human *GAPDH* promoter and chromatin immunoprecipitated by rabbit IgG were used as negative controls, and input DNA (2%) as an internal control. Numbers below lanes represent densitometry, normalized to input DNA and presented relative to that of untreated HuMoDCs (0 h). hu*CD40*, human *CD40*; hu*GAPDH*, human *GAPDH*. (F) Immunoblot analysis of Runx1 and Runx3 expression in HuMoDCs left untransfected, or transfected with control siRNA, Runx1 siRNA, Runx3 siRNA or Runx1 and Runx3 siRNAs (Runx1+3 siRNA). β-actin serves as a loading control. (G) Flow cytometry analysis of CD40 expression on HuMoDCs transfected with indicated siRNAs and then cultured with or without SAG for 24 h. (H) EMSA of nuclear extracts of HuMoDCs left uninfected or infected with promastigotes of Sb^R^LD strain GE1F8R or Sb^S^LD strain AG83 for 24 h and then cultured for 0.5 h with (+) or without (-) SAG; assayed with indicated probes. OCT-1 binding serves as an internal control. Numbers below lanes represent densitometry readings (as in Fig 2B); presented relative to control HuMoDCs (HuMoDCs left uninfected and cultured without SAG). (I) HuMoDCs were either left uninfected or infected with promastigotes of Sb^S^LD strain AG83 or Sb^R^LD strain GE1F8R for 24 h and then treated (for 24 h) with or without SAG. CD40 expression on HuMoDCs was assessed by flow cytometry. All data are representative of two independent experiments.

To extrapolate our findings in human DCs, we further verified the role of Runx in the regulation of SAG-induced CD40 expression by Sb^S^LD and Sb^R^LD in human monocyte-derived DCs (HuMoDCs). Initially, we tested whether Runx proteins regulate SAG-induced CD40 expression in HuMoDCs. Computational analysis using the TFBIND program identified two putative Runx-binding sites (R3: ^-456^TGTGGC^-451^ and R4: ^-433^GGTGGT^-428^; base positions are relative to the transcription initiation site) in the human *CD40* promoter (Fig 12A). Subsequent analyses involving EMSA, supershift EMSA and ChIP assays confirmed the binding of Runx1 and Runx3 to both R3 and R4 sites in *CD40* promoter following stimulation of HuMoDCs with SAG (Fig 12B–12E). Furthermore, silencing of Runx1 and/or Runx3 expression by siRNA greatly inhibited SAG-induced CD40 upregulation on HuMoDCs (Fig 12F and 12G). These results suggest that Runx1 and Runx3 are required for SAG-induced CD40 expression by HuMoDCs. Importantly, GE1F8R (Sb^R^LD), unlike AG83 (Sb^S^LD), impaired SAG-induced Runx binding to the *CD40* promoter and upregulation of CD40 expression on HuMoDCs (Fig 12H and 12I). Collectively, our data demonstrate that Sb^R^LD, but not Sb^S^LD, suppresses SAG-induced CD40 expression on DCs by preventing Runx binding to the *CD40* promoter. Sb^R^LD imparts this inhibitory effect on Runx and the upstream PI3K-Akt signaling pathway by prolonging SHP-1 activation even in the presence of SAG. Overall, our findings indicate the active involvement of Runx proteins in differential regulation of SAG-stimulated CD40 expression by Sb^R^LD and Sb^S^LD in DCs.

## Discussion

In VL, host protection versus exacerbation of the disease is largely determined by the level of CD40 expression on DCs [7]. However, it remains unclear how CD40 expression is regulated in DCs during LD infection. Additionally, the molecular mechanisms regulating CD40 expression are not fully understood. Here we have identified a key regulatory pathway for CD40 expression and evaluated its role in LD-mediated regulation of CD40 expression in DCs. Four key observations were made in this study.

First, we have identified two Runx-binding sites in mouse *CD40* promoter. Although each Runx-binding site individually contributed to the *CD40* promoter activity, the presence of both Runx-binding sequences was required for full promoter activation. Furthermore, both sites bound Runx1 and Runx3, but not Runx2, in response to LPS or TNFα stimulation. This is in accordance with a report demonstrating that Runx2 is expressed only in plasmacytoid DCs but not in other DC lineages [37]. Notably, both LPS and TNFα augmented the binding of Runx1 and Runx3 to the *CD40* promoter by facilitating their (i.e., Runx1 and Runx3) nuclear translocation. Our findings further suggest that Runx1 and Runx3 are essential for promoting LPS- and TNFα-mediated upregulation of CD40 expression in DCs. This conclusion is supported by results demonstrating that silencing of Runx1 and Runx3, or overexpression of Runx DN markedly attenuated LPS- or TNFα-induced CD40 expression in DCs. In this regard, the usage of conditional or DC-specific Runx1- and/or Runx3-deficient mice to assess the functional role for Runx1 and Runx3 in the regulation of CD40 expression in DCs was not feasible. This is due to the fact that the development of DCs is known to be severely compromised in Runx1 conditional knockout mice and DC-specific Runx3-deficient mice [38,39]. In addition, combined ablation of Runx1 and Runx3 (a primary requirement for this study) results in mouse lethality [40]. Nevertheless, our observations with Runx1- and Runx3-specific siRNAs, and Runx DN clearly indicate Runx1 and Runx3 as critical regulators of CD40 expression in DCs. Although Runx3 is reported to regulate the expression of other costimulatory molecules in DCs [11,41], the role of Runx transcription factors in the regulation of CD40 expression in DCs has remained unaddressed. Our findings therefore unveil a hitherto unknown role for Runx proteins in the regulation of CD40 expression in DCs.

Second, our study provides evidence supporting a role for PI3K-Akt pathway in Runx-mediated regulation of CD40 expression in DCs. For instance, pretreatment of BMDCs with PI3K inhibitors Wort or LY, or Akt inhibitor AI-XIII attenuated the capacity of LPS and TNFα to induce Runx binding to the *CD40* promoter and upregulate CD40 expression. In fact, blockade of PI3K with Wort or LY prevented LPS- and TNFα-induced nuclear translocation of Runx1 and Runx3. Previously, PI3K has been shown to regulate Runx1 and Runx3 expression in T cells [42]. Based on this report, one might argue that the PI3K inhibitors impaired Runx binding to the *CD40* promoter by downregulating Runx expression. This possibility, however, was ruled out by our observation that BMDC treatment with PI3K inhibitors for 1 h did not affect the expression of Runx proteins. Furthermore, the duration of PI3K inhibitor treatment (24 h) and the cell type (T cells) used in the above-mentioned report [42] are quite different from those used in the present study (1 h and primary DCs, respectively). Thus, our results suggest that PI3K regulates CD40 expression in DCs by controlling nuclear translocation and the DNA binding activity of Runx1 and Runx3 to the *CD40* promoter. While exploring the type of PI3K isoform(s) involved in Runx-mediated regulation of CD40 expression in DCs, we detected a p85α-p110β PI3K complex associated with TLR4 and TNFR1 following LPS and TNFα stimulation, respectively. Blocking the expression of p110β PI3K isoform prevented Runx binding to the *CD40* promoter as well as CD40 upregulation induced by LPS and TNFα. Currently, reports about the type of PI3K isoforms involved in TLR4 signaling in DCs seem contradictory. Vanhaesebroeck and colleagues have reported that PI3K p110δ but not p110β isoform acts as a main driver of LPS-induced PI3K-Akt signaling via TLR4 [43]. In contrast, the Utsugi group demonstrated p110β as a key mediator of the same signaling pathway in DCs [44]. In this regard, our results are consistent with the latter report. An essential role for p110β in LPS-induced TLR4 signaling has been demonstrated in mouse and human macrophages also [44,45]. Together, our findings document TLR4/TNFR-PI3K (p85α-p110β)-Akt-Runx as a newly defined signaling pathway that drives CD40 upregulation on DCs.

Third, our finding that LD downregulates LPS- and TNFα-stimulated CD40 expression in DCs by inhibiting nuclear translocation and the DNA binding activity of Runx proteins to the *CD40* promoter describes a new mechanism for LD-mediated regulation of CD40 expression. LD exhibited these inhibitory effects on Runx by suppressing the PI3K-Akt pathway. This outcome of LD infection exactly mimicked the effects of PI3K inhibitors, which also diminished LPS- and TNFα-induced CD40 upregulation on DCs by preventing nuclear translocation and subsequent binding of Runx proteins to the *CD40* promoter. Our findings contrast a previous study showing that *L*. *infantum* (another visceral strain) inhibits CD40 expression in DCs by inducing PI3K activation [46]. It is possible that the distinct effects of PI3K on regulation of CD40 expression in DCs may be parasite species specific. We also showed that LD inhibits LPS- and TNFα-induced CD40 upregulation on DCs via SHP-1. When SHP-1 was suppressed, LD could not inhibit LPS- and TNFα-stimulated CD40 upregulation. A previous study in the context of *L*. *major* infection has demonstrated that SHP-1 interferes with the CD40-induced signaling pathway and thereby attenuates CD40-induced antileishmanial functions [47]. However, whether SHP-1 suppresses CD40 expression and that too by inhibiting Runx activity is currently unknown. Our results now suggest an “additional” role for SHP-1 by demonstrating that SHP-1, in response to LD infection, also inhibits CD40 expression. SHP-1 mediated this inhibitory effect by blocking the PI3K-Akt pathway and downstream Runx binding to the *CD40* promoter. For example, suppression of SHP-1 restored the ability of LPS or TNFα to induce PI3K-Akt signaling and drive Runx-mediated CD40 upregulation despite LD infection. Thus, our findings have identified a SHP-1-dependent mechanism by which LD downregulates LPS- and TNFα-induced CD40 expression in DCs.

The fourth key observation made in this study is that the antileishmanial drug SAG upregulates CD40 expression on DCs and promotes antileishmanial response of DCs *in vivo* in a Runx-dependent manner. In fact, SAG, like LPS and TNFα, upregulated CD40 expression on uninfected DCs by promoting nuclear translocation and the binding of Runx proteins to the *CD40* promoter via the PI3K-Akt pathway. Interestingly, the same PI3K-Akt-Runx pathway was utilized by SAG to upregulate CD40 on LD-infected DCs. Conversely, LPS- or TNFα-induced PI3K-Akt-Runx pathway could not upregulate CD40 expression on LD-infected DCs. These findings demonstrated the unique ability of SAG to upregulate CD40 expression on LD-infected DCs. SAG could mediate this effect because, being a potent inhibitor of SHP-1 [29–36], SAG effectively blocked LD-induced SHP-1 activation which LPS and TNFα could not. A published report has shown that SHP-1, upon activation, binds to the p85α subunit of PI3K and therefore directly inhibits PI3K-Akt signaling [22]. Accordingly, blockade of LD-induced SHP-1 activation and thereby preventing SHP-1-PI3K interaction by SAG prompted the PI3K-Akt-Runx pathway to upregulate CD40 expression on LD-infected DCs. Notably, these SAG-induced molecular events leading to CD40 upregulation on DCs were apparent only when DCs were infected with normal LD (i.e., Sb^S^LD). Sb^R^LD, on the other hand, largely impaired the ability of SAG to trigger PI3K-Akt signaling and subsequent Runx-mediated CD40 upregulation on DCs by persistently activating SHP-1 and therefore prolonging SHP-1-PI3K interaction. Given such findings, we propose SHP-1 as a “direct” molecular link through which Sb^S^LD and Sb^R^LD regulate SAG-mediated activation of the PI3K-Akt-Runx pathway and CD40 expression in DCs. Our data further demonstrated that similar to murine DCs, Sb^R^LD inhibited SAG-induced CD40 expression in HuMoDCs as well. Sb^R^LD mediated this inhibitory effect in HuMoDCs by blocking Runx binding to the *CD40* promoter. These results suggest a key role for Runx in the differential regulation of SAG-induced CD40 expression by Sb^R^LD and Sb^S^LD independent of the origin of DCs (e.g. mouse and human origin). Importantly, the enhanced CD40 expression in response to SAG treatment is not solely dependent on the transcriptional activity of Runx1 and Runx3. Other transcription factors may also contribute to the CD40 upregulation on DCs mediated by SAG. Alternatively, SAG-stimulated CD40 expression may be regulated at post-transcriptional and/or post-translational levels. Nevertheless, our results document Runx1 and Runx3 as critical mediators of SAG-induced CD40 upregulation on DCs. A previous study has reported that the antileishmanial efficacy of SAG relies on CD40-CD40L interaction, which elicits host-protective immune response by inducing secretion of a Th1-promoting cytokine IL-12 [48,49]. In fact, any drug or drug combination that triggers CD40-mediated IL-12 production is believed to ameliorate *Leishmania* infection by restoring Th1 responses [50]. As mentioned above, IL-12 is secreted early by DCs following LD infection and its production is largely influenced by the level of CD40 expression on DCs [5,7]. It is therefore likely that the antileishmanial effect of SAG depends on CD40 expression by DCs. In line with this hypothesis, we showed here that suppressing Runx1 and Runx3 expression and thereby preventing CD40-mediated type-1 T cell response attenuated SAG-induced antileishmanial effects of DCs *in vivo*. Our findings thus underscore the importance of Runx proteins in the context of antileishmanial therapy.

**Fig 13:**
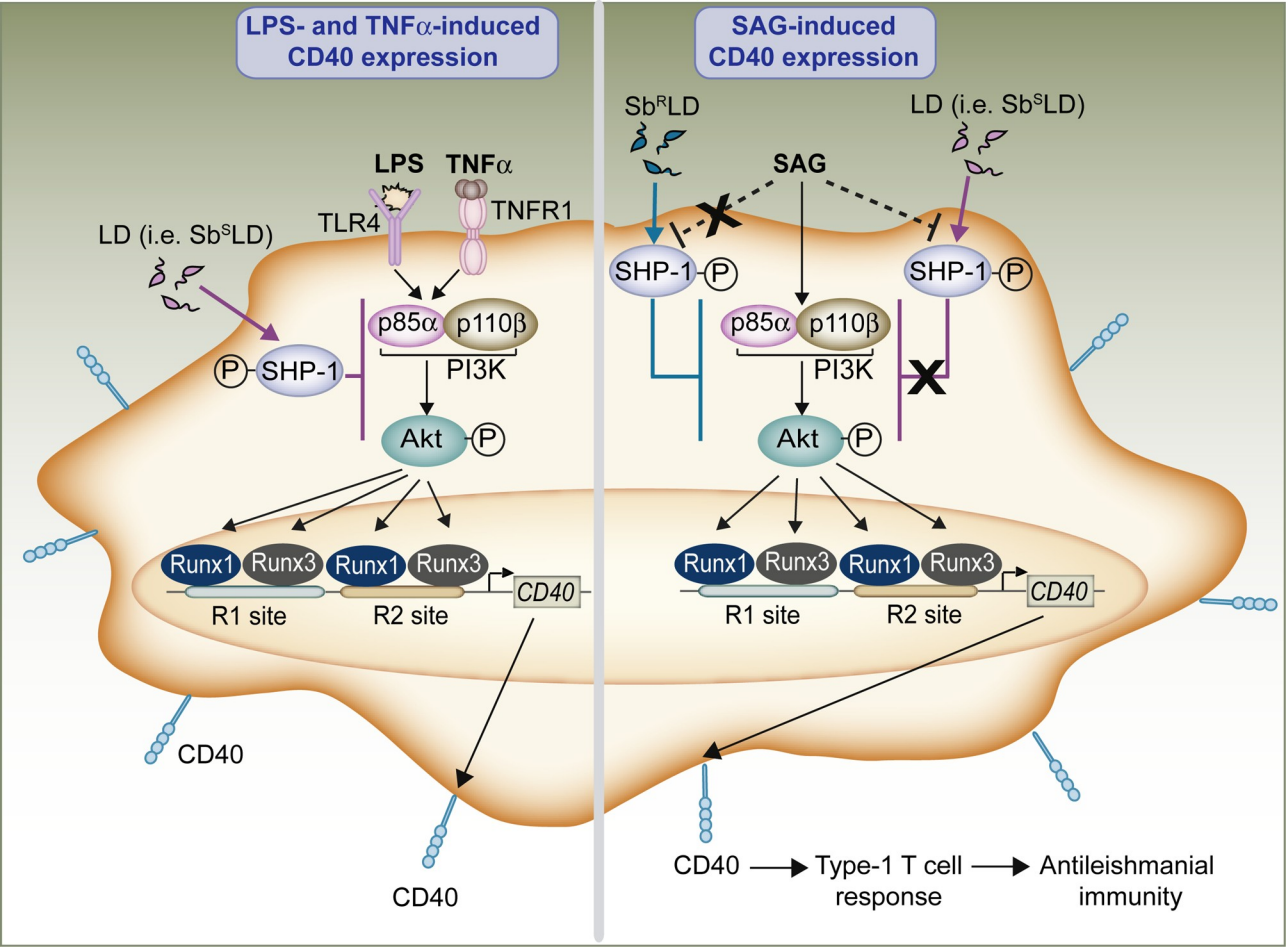
Model depicting the Runx-CD40 axis as a molecular key for antileishmanial immunity. Our results show that the proinflammatory mediators LPS and TNFα, and antileishmanial drug SAG, through PI3K (p85α-p110β)-Akt signaling pathway, induce the binding of Runx1 and Runx3 transcription factors to the *CD40* promoter and consequently upregulate CD40 expression on DCs. The CD40 upregulation induced by LPS or TNFα, however, is inhibited by normal LD (i.e., Sb^S^LD; left panel). LD mediates this inhibitory effect by suppressing the PI3K (p85α-p110β)-Akt-Runx pathway through SHP-1. In contrast, SAG-induced CD40 upregulation on DCs remains unaffected by normal LD infection (right panel). This is because SAG, by blocking (directly/indirectly; indicated by dotted lines) LD-induced SHP-1 activation, enables the PI3K (p85α-p110β)-Akt -Runx pathway to upregulate CD40 expression on LD-infected DCs. The upregulated CD40 expression in turn augments type-1 T cell responses that confer protection against LD infection. Unlike normal LD (Sb^S^LD); Sb^R^LD, however, suppresses the PI3K-Akt-Runx pathway-mediated CD40 upregulation on DCs by persistently activating SHP-1 despite SAG treatment. Thus, acting as a critical mediator of CD40 expression in DCs, Runx proteins play a pivotal role in mounting antileishmanial immunity.

In summary, our findings elucidate the role of Runx proteins in the regulation of CD40 expression in DCs and have identified the PI3K-Akt pathway as controlling this process. We have also demonstrated that LD (i.e., Sb^S^LD) generally downregulates CD40 expression in DCs by suppressing the PI3K-Akt-Runx signaling axis through SHP-1. The antileishmanial drug SAG, on the other hand, upregulates CD40 on DCs via the same PI3K-Akt-Runx pathway despite LD infection by directly/indirectly blocking SHP-1 activity. The elevated CD40 expression by DCs in turn promotes type-1 T cell responses, leading to the development of antileishmanial immunity. Importantly, Sb^R^LD suppresses the PI3K-Akt-Runx pathway-mediated SAG-induced CD40 upregulation on DCs by persistently activating SHP-1 (Fig 13). These findings provide evidence for an immunoregulatory role of Runx transcription factors in VL. Overall, our study reveals a unique mechanism regulating CD40 expression in DCs and suggests its pivotal role in DC-mediated antileishmanial immune responses. Given that CD40 on DCs plays a host-protective role in *Leishmania* infection [7], potentiating Runx-driven CD40 expression in DCs might be therapeutically beneficial in VL. Furthermore, our study will contribute to better understanding of the role of Runx proteins in the regulation of host immune responses during other pathogenic infections.

## Materials and methods

### Ethics statement

The use of LD for this study was approved by the Biosafety Committee of Institute of Microbial Technology (#IMTECH/IBSC/2015/15 and CSIR-IMTECH/IBSC/2018/22). All animal studies were approved by the Institutional Animal Ethics Committee of the Institute of Microbial Technology (#IAEC/16/10) and performed according to the National Regulatory Guidelines issued by CPSEA (Committee for the Purpose of Supervision of Experiments on Animals), Govt. of India. For HuMoDC preparation, the buffy coats of healthy donors were obtained from the Postgraduate Institute of Medical Education and Research, Chandigarh, India, with approval of the Institutional Ethics Committees of Institute of Microbial Technology [IEC (May 2020) #1] and Postgraduate Institute of Medical Education and Research (PGI/IEC/2020/EIC000344). Informed consents were obtained from all blood donors.

### Reagents

The following antibodies were used for immunoblot analysis: anti-Runx1 (sc-28679), anti-Runx3 (sc-376543), anti-PI3K p110β (sc-376412), anti-PI3K p110δ (sc-7176), anti-TLR4 (sc-293072), anti-β-actin (sc-47778), HRP-conjugated anti-rabbit IgG (sc-2004) and HRP-conjugated anti-mouse IgG (sc-2005; all from Santa Cruz Biotechnology); anti-phospho-SHP-1 (Tyr536, ab51171; Abcam); and anti-PI3K p85α (4292), anti-PI3K p110α (4255), anti-phospho-Akt (Ser473; 9271), anti-Akt (9272), anti-phospho-SHP-1 (Tyr564; 8849), anti-SHP-1 (3759) and anti-TNFR1 (13377; all from Cell Signaling Technologies). For EMSA, ChIP and immunoprecipitation assays, the antibodies and reagents used were as follows: anti-Runx1 (sc-28679 X), anti-Runx2 (sc-10758 X), rabbit IgG (sc-2027), mouse IgG (sc-2025) and protein A/G PLUS-Agarose beads (sc-2003; all from Santa Cruz Biotechnology); anti-Runx3 (39301; Active Motif); Armenian hamster IgG (400901, Biolegend); and anti-TLR4 (ab22048) and anti-TNFR1 (ab106099; both from Abcam). For confocal microscopy following antibodies were used: anti-Runx1 (sc-28679), anti-Runx3 (sc-376543), and respective isotype control antibodies [rabbit IgG (sc-2027) for anti-Runx1, and mouse IgG (sc-2025) for anti-Runx3] were obtained from Santa Cruz Biotechnology. Alexa Fluor 568 (AF)-conjugated goat anti-rabbit IgG (A-11011) and goat anti-mouse IgG (A-11004) secondary antibodies were procured from Thermo Fisher Scientific. For flow cytometry analysis, following antibodies were obtained from Biolegend: APC-conjugated anti-mouse CD11c (117310), APC-conjugated anti-mouse CD3 (100235), FITC-conjugated anti-mouse CD8 (100705), PE-conjugated anti-mouse IL-10 (505007), PE-conjugated anti-mouse CD40 (124610), PE-conjugated anti-mouse TNFR1 (113003), PE-conjugated anti-human CD40 (313006), PE-conjugated anti-mouse TNFR2 (113405), PE-conjugate Armenian hamster IgG (400907), PE-conjugated rat IgG2a,κ (400507), PE-conjugated mouse IgG1,κ (400111), APC-conjugated Armenian hamster IgG (400911) and APC-conjugated rat IgG2b,κ (400611). Other antibodies such as FITC-conjugated anti-mouse CD4 (11-0041-82), PE-conjugated anti-mouse IFNγ (12-7311-83) and PE-conjugated anti-mouse TLR4 (12-9041-80) were procured from eBioscience (Thermo Fisher Scientific); and FITC-conjugated rat IgG (sc-2340) and PE-conjugated rat IgG (sc-516648) were from Santa Cruz Biotechnology. The Akt inhibitor XIII (AI-XIII; 124030) was from Merck Life Science. Recombinant mouse TNFα, granulocyte-macrophage colony-stimulating factor (GM-CSF) and IL-4 were from Peprotech. Recombinant human GM-CSF and IL-4, and anti-mouse CD11c magnetic beads were from Miltenyi Biotec. The ON-TARGETplus non-targeting control pool siRNAs and SMARTpool siRNAs targeting mRNA encoding mouse Runx1, Runx3, PI3K p110β and SHP-1; and human Runx1 and Runx3 were from Dharmacon. Notably, the same siRNAs were previously used by other groups [51–54]. LPS (*E*. *coli* O111:B4), PI3K inhibitors Wortmannin (Wort; W3144) and Ly294002 (LY; L9908), and all other reagents were procured from Sigma-Aldrich unless stated otherwise.

### Animals

BALB/c mice and golden hamsters (*Mesocricetus auratus*) were maintained and bred under pathogen-free conditions at the Institute of Microbial Technology animal facility.

### Parasite culture

The LD strain AG83 [MHOM/IN/83/AG83; American Type Culture Collection (ATCC PRA-413)], provided by Dr. Nahid Ali (CSIR-Indian Institute of Chemical Biology, India), was maintained in male or female golden hamsters between 4–6 wks of age as described [55]. Amastigotes were isolated from spleens of infected hamsters as described [56]. Subsequently, amastigotes were transformed into promastigotes and maintained as described [57]. This LD strain AG83 was also used as Sb^S^LD [28] in some experiments. For some comparative analyses with Sb^S^LD strain AG83, the Sb^R^LD strain GE1F8R (MHOM/IN/89/GE1) [28] was used.

### DC preparation and treatment

BMDCs and sDCs were prepared from male or female BALB/c mice between 8–12 wks of age as described [58]. In some experiments, HuMoDCs, either prepared from human buffy coats as described [58] upon approval by the Ethical Committees mentioned above or purchased from Lonza (CC-2701), were used. DCs (5 x 10^6^/well) were then treated with LPS (1 μg/ml) or TNFα (100 ng/ml) for specified times in complete RPMI 1640 medium (10% FBS, penicillin/streptomycin, L-glutamine and 2-mercaptoethanol). Alternatively, DCs were treated with clinical grade of SAG (40 μg/ml; a gift from Albert David Ltd., Kolkata, India) for specified times as described [28]. The dose of SAG mentioned here represents the concentration of Sb^V^. For blocking PI3K or Akt activation, DCs were treated with PI3K inhibitors Wort (200 nM) or LY (50 μM) or Akt inhibitor AI-XIII (5 μM) for 1 h prior to LPS, TNFα or SAG stimulation. In some other experiments, DCs (2.5 x 10^5^) were transfected with 500 ng green fluorescent protein (GFP)-tagged Runx DN or empty vector (a gift from Dr. Vanja Lazarevic, National Cancer Institute, NIH, USA; [19]) using the TransIT-2020 transfection reagent (Mirus) before LPS stimulation.

### DC infection with LD

DCs (5 x 10^6^/well) were infected *in vitro* with amastigotes or promastigotes (stationary phase) of LD (i.e., Sb^S^LD) at parasite to DC ratio (multiplicity of infection; MOI) of 10:1 for indicated times in RPMI 1640 complete medium. Subsequently, DCs were washed, resuspended in RPMI 1640 complete medium and stimulated with LPS, TNFα or SAG for specified times. In some cases, DCs (5 x 10^5^ cells in 500 μl RPMI 1640 complete medium) were adhered on 18 mm x 18 mm cover slips, infected with promastigotes of LD (i.e., Sb^S^LD) for 24 h (parasite to DC ratio of 10:1) and then treated with SAG for indicated times. The number of intracellular parasites in DCs was determined via Giemsa staining. For some experiments, DCs were transfected with empty vector (pCMV) or SHP-1 DN-expressing vector (pCMV-SHP-1 DN (C453S); a gift from Dr. Yuping Lai, East China Normal University, Shanghai, China [59]) using the TransIT-2020 transfection reagent (Mirus) prior to LD infection. In other experiments, DCs were infected with promastigotes of Sb^R^LD at parasite to DC ratio of 10:1 for 24 h in RPMI 1640 complete medium and then stimulated with SAG.

### Quantitative RT-PCR

The cDNA synthesis and quantitative RT-PCR were carried out using the SuperScript III Platinum SYBR Green one-step qRT-PCR kit (Invitrogen) and the following primers: mouse *CD40*, forward 5'-GCTATGGGGCTGCTTGTTGA-3' and reverse 5' -ATGGGTGGCATTGGGTCTTC-3' [60]; and mouse *ACTB* (encoding β-actin), forward 5′-GCTCTGGCTCCTAGCACCAT-3′ and reverse 5′-GCCACCGATCCACACCGCGT-3′ [61]. Gene expression was measured by the change in threshold method (ΔΔC_T_) and normalized to the ACTB mRNA expression.

### *In vivo* footprinting

Dimethylsulfate (DMS) treatment of DCs and naked DNA, ligation-mediated PCR and *in vivo* footprint analyses were done as described [62]. Details of primers used for footprint analyses are mentioned in S1 Table.

### EMSA

Nuclear extracts were prepared as described [63]. EMSA was performed as described [64] using various P^32^-labeled DNA probes (S1 Table) specific for mouse or human *CD40* promoter. An OCT-1 probe, 5'-TGTCGAATGCAAATCACTAGAA-3', was used as control. Supershift analysis was performed as described [65] using 4 μg of rabbit IgG, anti-Runx1, anti-Runx2 or anti-Runx3. Bands were visualized using a phosphoimager (Fujifilm FLA-9000).

### ChIP

ChIP assay was performed using the ChIP-IT kit (Active Motif) and antibodies anti-Runx1, anti-Runx2, anti-Runx3 or rabbit IgG. After immunoprecipitation, followed by DNA extraction, PCR was performed to amplify -578/-373 region of mouse *CD40* promoter using primers: P1, 5'-GCTGTCCCCTACTCGTAGGAATTTCCTTCT-3' and P2, 5'-GTTACCCCACCCCACCCACAATACCCC-3'. For a negative control, mouse glyceraldehyde-3-phosphate dehydrogenase (*GAPDH*) promoter was amplified by using primers: 5'-CACCCTGGCATTTTCTTCCA-3' and 5'-GACCCAGAGACCTGAATGCTG-3' [28]. Alternatively, PCR was carried out to amplify -480/-276 region of human *CD40* promoter using primers: P3, 5'-TGAAACGCCTCCCCATAC-3' and P4, 5'- CTGCGACCGGAGAGAG-3'. In that case, amplification of human *GAPDH* promoter using primers 5'-GCCTGAGCAGTCCGGTGT-3' and 5'-GATCGGTGCTGGTTCCCA-3' [66] served as a negative control.

### Immunoprecipitation and immunoblot analysis

Crosslinking of proteins in DCs was performed using dimethyl 3,3-dithiopropionimidate dihydrochloride (Sigma-Aldrich) as described [58]. DCs were subsequently lysed with the cell lysis buffer (Cell Signaling Technology). Immunoprecipitation and immunoblot analysis were performed as described [58].

### Densitometry analysis

Densitometry analysis was performed using Scion Image software (Scion Corporation).

### Reporter assay

The pGL3-luciferase reporter plasmid containing wild-type mouse *CD40* promoter fragment (-505 to +22 region, represented here as p505-luc and previously described as D9 [8]) was provided by Prof. Herman Waldmann (University of Oxford, UK). The mutant *CD40* promoter-luciferase constructs p505-MutR1-luc, p505-MutR2-luc and p505-Mut(R1+R2)-luc, in which the Runx-binding sites R1 (^-489^TGTGGT^-484^), R2 (^-464^TGCGGT^-459^) or both R1 and R2 were substituted with GAATTC sequence, were created by Mutagenex Inc. JAWSII cells (American Type Culture Collection; 5 x 10^5^/well) were transfected with either of the above-mentioned reporter constructs (900 ng) together with a control renilla luciferase reporter pRL-CMV (100 ng) using the TransIT-2020 transfection reagent (Mirus). After 24 h, cells were either left untreated or treated for 24 h with LPS or TNFα. Luciferase activity was assayed with the dual-luciferase reporter assay kit (Promega).

### RNA-mediated interference

DCs were transfected with 60 nM siRNA using Lipofectamine RNAiMAX reagent (Invitrogen).

### Confocal microscopy

Immunostaining and confocal microscopy were performed as described [67]. Briefly, BMDCs (2.5 x 10^5^) were adhered to coverslips (12 mm) for 6 h and stimulated with LPS, TNFα or SAG for 0.5 h. In some experiments, DCs were treated with PI3K inhibitors Wort or LY for 1 h, or infected with promastigotes of normal LD (AG83; Sb^S^LD strain) or antimony-resistant LD (GE1F8R; Sb^R^LD strain) for 24 h prior to LPS, TNFα or SAG stimulation as specified. Cells were washed with phosphate buffered saline (PBS), fixed for 5 min in 4% paraformaldehyde, permeabilized with 0.1% Triton X-100 for 5 min and then incubated with 3% BSA for 1 h at room temperature. Subsequently, cells were incubated with anti-Runx1, anti-Runx3 or corresponding isotype control antibody (all 1: 500 dilutions) for 1 h at room temperature. After washing, cells were further incubated for 1 h at room temperature with Alexa Fluor 568-conjugated goat anti-rabbit IgG or goat anti-mouse IgG (1: 1000 dilution). For staining of nuclei, Hoechst (Thermo Fisher Scientific) was used. After mounting, images were acquired with a NIKON A1R Laser scanning confocal microscope. Images were processed using NIS-Elements version 4.13.01 and Adobe Photoshop software.

### Assessment of CD40 expression on sDCs derived from LD-infected mice

BALB/c mice between 4–6 wks of age were injected i.v. with stationary phase LDPm (2 x 10^7^/mouse) or left uninfected. After 45 days, splenocytes were prepared from these LD-infected mice or age-matched uninfected mice and stimulated with LPS for 24 h. The level of CD40 expression on sDCs was measured via flow cytometry after gating on CD11c^+^ population.

### DC transfer experiments

BALB/c BMDCs were transfected or not with Runx1- and Runx3-specific siRNAs or with control siRNA and then stimulated with SAG for 24 h. In some experiments, prior to SAG stimulation, Runx1 and Runx3 siRNA-treated DCs were transfected with 2 μg empty vector or CD40-expressing vector (pcDNA3-CD40) using the TransIT-2020 transfection reagent (Mirus). DCs were then injected i.v. into LD-infected BALB/c mice (on days 15, 25, 35 and 45 postinfection). On day 48 postinfection, spleens and livers were isolated to measure their weights and parasite burden, and the frequencies of splenic CD4^+^ and CD8^+^ T cells producing IFNγ or IL-10. The spleen and liver parasite burden was measured by stamp-smear method and is presented as Leishman-Donovan Unit (LDU) [68]. For detection of IFNγ and IL-10 within CD4^+^ and CD8^+^ T cells, the RBC-depleted splenocytes were first surface-stained with anti-CD3-APC together with anti-CD4-FITC, anti-CD8-FITC or rat IgG-FITC (isotype control). Splenocytes were then subjected to intracellular staining using a Fixation/Permeabilization Buffer kit (eBioscience; 88-8823-88), and PE-labeled anti-mouse IFNγ, anti-mouse IL-10 or rat IgG (isotype control). The percentage of IFNγ- or IL-10-producing CD4^+^ and CD8^+^ cells in the CD3-gated cell population was determined via flow cytometry. The mouse CD40 cDNA used in this experiment was obtained from Prof. David Wagner (University of Colorado, USA) and cloned into a pcDNA3 plasmid (Invitrogen) using BamHI and XhoI sites.

### Flow cytometry

Flow cytometry was performed with a C6 Accuri flow cytometer (BD Biosciences). Data were analyzed with FlowJo software (Tree Star).

### Statistical analysis

One-way ANOVA (SigmaPlot 11.0 program) was used for statistical analyses. A “*p*” value < 0.05 was considered significant.

## Supporting information

S1 Data Excel spreadsheet. Excel spreadsheet containing, in separate sheets, the numerical values and statistical analysis for Figure panels 3B, 3E, 6G, 7I, 9B, 9C, 10C, 11A, S1A, S1B, S1C, S2, S6C, S7A, S7B, S8A, S8B, S11, S12A, S12B, S12C, S14A, S14B, S15A, S15B, S16, S20A and S20B.
(XLSX)

S1 Fig Graphical presentation of MFI of LPS- or TNFα-induced CD40 expression in DCs following LD infection. Relates to Fig 1A, 1B and 1D. BMDCs were either left uninfected or infected with LDPm (A; relates to Fig 1A) or LDAm (B; relates to Fig 1B) for indicated times and then stimulated with LPS for 24 h or left unstimulated. In some experiments (C; relates to Fig 1D), BMDCs were infected for 24 h with LDPm and stimulated with TNFα for 24 h. The expression of CD40 was analyzed by flow cytometry (shown in Fig 1A, 1B and 1D). The mean fluorescence intensity (MFI) of corresponding CD40 expression was calculated after subtracting isotype background and is presented here as fold change relative to control DCs (DCs; i.e., DCs left uninfected and unstimulated). Data are a compilation of three separate experiments. Error bars represent SD. Each symbol represents data of individual experiment. *p < 0.001, **p < 0.01, ***p < 0.05; NS, not significant.
(TIF)

S2 Fig Densitometry of LPS- or TNFα-induced nuclear protein binding to putative Runx-binding sites in *CD40* promoter. Relates to Fig 2B. BMDCs were treated with LPS or TNFα for indicated times. The binding of nuclear proteins to mouse *CD40* promoter-specific probes (Pr1 and Pr2) that contained putative Runx-binding sites was determined via EMSA (shown in Fig 2B). Bar graphs here show pooled densitometry results (*n* = 3 independent experiments) for intensity of nuclear protein binding to indicated probes. Densitometry analysis was performed as in Fig 2B and data are presented relative to untreated BMDCs (0 h). Each symbol represents data of individual experiment. *p < 0.001, **p < 0.01, ***p < 0.05; NS, not significant.
(TIF)

S3 Fig LPS and TNFα induce nuclear-protein binding to putative Runx sites on *CD40* promoter in sDCs. BALB/c sDCs were treated with LPS or TNFα for 0.5 h or left untreated. Nuclear extracts were subjected to EMSA using indicated probes (as in Fig 2B). Numbers below lanes represent densitometry [normalized to OCT-1 binding (control)] relative to that of untreated (UT) sDCs. Data are representative of three independent experiments.
(TIF)

S4 Fig LPS and TNFα do not trigger Runx protein recruitment to the mouse *GAPDH* promoter. Relates to Fig 2E. BMDCs were treated with LPS or TNFα for indicated times. The recruitment of Runx proteins to the mouse *GAPDH* promoter was examined by ChIP using indicated antibodies (left margin) and the primers described in “Materials and methods”. Amplification of chromatin immunoprecipitated by rabbit IgG served as a negative control, and input DNA (2%) as an internal control. Data are representative of three independent experiments.
(TIF)

S5 Fig Confocal microscopy images of DCs immunostained with isotype control antibodies. Relates to Fig 2G. BMDCs were stained with Hoechst (blue; stains all nuclei) and immunolabeled with rabbit IgG (isotype control for anti-Runx1) or mouse IgG (isotype control for anti-Runx3), followed by Alexa Fluor 568-conjugated goat anti-rabbit IgG or goat anti-mouse IgG (red) secondary antibody, respectively. Cells were analyzed via confocal microscopy. The lack of red fluorescence within cytoplasm and/or nuclei (“rabbit IgG + anti-rabbit IgG-AF” and “mouse IgG + anti-mouse IgG-AF” panels), and the absence of pink color within nuclei in merged images (“merge” panels) confirm the specificity of anti-Runx1 and anti-Runx3 antibodies used in Fig 2G and all other experiments. Scale bar, 10 μm. AF, Alexa Fluor 568. Data are representative of two independent experiments.
(TIF)

S6 Fig Runx-binding sites control *CD40* promoter activity. (A) Immunoblot analysis of Runx1 and Runx3 in JAWSII cell lysates. (B) Schematic of the mouse *CD40* promoter-firefly luciferase (luc) reporter constructs used in reporter assay. Expression of firefly luciferase is controlled by wild-type mouse *CD40* promoter fragment (-505 to +22 region; p505-luc) or similar *CD40* promoter fragment carrying mutations in either of the Runx-binding sites (p505-MutR1-luc or p505-MutR2-luc) or both Runx-binding sites [p505-Mut(R1+R2)-luc]. (C) Dual-luciferase assay of JAWSII cells that had been transfected with indicated *CD40* promoter-luciferase constructs (as in B) together with the renilla luciferase plasmid pRL-CMV (internal control) and then treated for 24 h with LPS (left panel) or TNFα (right panel) or left untreated (UT). Results were normalized to the activity of renilla luciferase and are presented relative to those in p505-luc-transfected JAWSII cells that had been left untreated. Horizontal bars represent the mean. Data are a compilation of two separate experiments (*n* = 5 in one experiment, and *n* = 3 in another experiment). Each symbol represents data of individual replicate. *p < 0.001.
(TIF)

S7 Fig MFI of LPS- or TNFα-stimulated CD40 expression in DCs after Runx1 and/or Runx3 silencing. Relates to Fig 3C and 3F. BMDCs were left untransfected (- siRNA) or transfected with control (Ctrl) siRNA, Runx1 siRNA, Runx3 siRNA or Runx1 and Runx3 siRNAs (Runx1+3 siRNA) and then cultured with (+) or without (-) LPS for 24 h (A; relates to Fig 3C). In some experiments (B; relates to Fig 3F), BMDCs were transfected with siRNAs as described above, and then cultured with (+) or without (-) TNFα for 24 h. The expression of CD40 on BMDCs assessed by flow cytometry has been shown in Fig 3C and 3F. Corresponding MFI data of CD40 expression (assessed as in S1 Fig) pooled from three independent experiments are shown in bar graphs, and presented as fold change relative to BMDCs left untransfected and cultured without LPS (A) or TNFα (B). Error bars represent SD. Each symbol represents data of individual experiment. *p < 0.001, **p < 0.01, ***p < 0.05; NS, not significant.
(TIF)

S8 Fig MFI data of CD40 expression and densitometry results related to Fig 4A and 4B. (A) Relates to Fig 4A. BMDCs were left untreated (UT) or treated with DMSO (0.1%; control treatment), Wort or LY for 1 h and then cultured for 24 h with (+) or without (-) LPS (left panel) or TNFα (right panel). The expression of CD40 on BMDCs assessed by flow cytometry has been shown in Fig 4A. The MFI data of CD40 expression (assessed as in S1 Fig) for Fig 4A pooled from three independent experiments are depicted in bar graphs, and presented as fold change relative to untreated BMDCs cultured without LPS (left panel) or TNFα (right panel). Error bars represent SD. Each symbol represents data of individual experiment. (B) Relates to Fig 4B. BMDCs were left untreated or treated for 1 h with DMSO, Wort or LY and then left unstimulated (US) or stimulated with LPS or TNFα for 0.5 h. The EMSA and immunoblot data have been shown in Fig 4B. Here, bar graphs depict pooled densitometry results (*n* = 3 independent experiments) for Fig 4B measuring the intensity of Runx1 and Runx3 binding to the mouse *CD40* promoter-specific probes (Pr1 and Pr2; left two panels), and the expression of Runx1 and Runx3 in BMDC lysates (right two panels). Data are presented relative to untreated BMDCs that had been left unstimulated. Error bars represent SD. Each symbol represents data of individual experiment. *p < 0.001, **p < 0.01, ***p < 0.05; NS, not significant.
(TIF)

S9 Fig Blockade of PI3K-Akt pathway prevents LPS- or TNFα-stimulated nuclear translocation of Runx1 and Runx3. BMDCs were either left untreated (UT) or treated for 1 h with DMSO, Wort or LY and then stimulated with LPS or TNFα for 0.5 h or left unstimulated (US). The translocation of Runx1 and Runx3 (red) to the nuclei (blue) was analyzed via confocal microscopy. Pink color (merge) shows nuclear translocation of Runx1 or Runx3. Scale bar, 10 μm. Data are representative of two independent experiments.
(TIF)

S10 Fig LPS and TNFα induce Akt phosphorylation in DCs. Immunoblot analysis of total and phosphorylated Akt in lysates of BMDCs treated with LPS (A) and TNFα (B) for indicated times. Numbers below lanes represent densitometry, normalized to total Akt and presented relative to untreated BMDCs (0 h). Data are representative of two independent experiments.
(TIF)

S11 Fig Densitometry results related to Fig 5A. BMDCs were left uninfected (Uninf) or infected with LDPm for indicated times and then stimulated with (+) LPS or TNFα for 0.5 h or left unstimulated (-). The binding of nuclear Runx1 and Runx3 to the *CD40* promoter (analyzed via EMSA using Pr1 and Pr2 probes) and the expression of Runx1 and Runx3 in BMDC lysates (determined by immunoblot analysis) have been shown in Fig 5A. Here, the bar graphs show corresponding densitometry results pooled from three independent experiments. Data are presented relative to uninfected BMDCs that had been left unstimulated. Error bars represent SD. Each symbol represents data of individual experiment. *p < 0.001, **p < 0.01, ***p < 0.05; NS, not significant.
(TIF)

S12 Fig MFI data of CD40 expression and densitometry results related to Fig 6. (A) Relates to Fig 6A. The bar graphs represent pooled densitometry results (*n* = 3 independent experiments) for immunoblot analysis (shown in Fig 6A) of SHP-1 phosphorylated at Tyr536 or Tyr564 in BMDCs infected with LDPm for indicated times. For additional details related to densitometry analysis, see Fig 6A. Data are presented relative to uninfected BMDC (0 h). (B) Relates to Fig 6F. BMDCs were transfected with indicated siRNAs, infected with LDPm for 24 h and then treated with or without LPS or TNFα for 0.5 h. The EMSA and immunoblot analysis data have been shown in Fig 6F. Here, bar graphs show corresponding densitometry results (pooled from three independent experiments) for Runx1 and Runx3 binding to the mouse *CD40* promoter-specific probes (Pr1 and Pr2; upper panels), and the expression of Runx1 and Runx3 in BMDC lysates (lower panels). Densitometry analysis was carried out as described in Fig 6F. Data are presented relative to control BMDCs (BMDCs left untransfected and uninfected, and given no LPS or TNFα treatment). (C) Relates to Fig 6H. BMDCs were transfected with indicated siRNAs, then infected with LDPm for 24 h and cultured with (+) or without (-) LPS (left panel) or TNFα (right panel) for 24 h. The expression of CD40 on BMDCs (assessed by flow cytometry) has been shown in Fig 6H. Corresponding MFI of CD40 expression was calculated as in S1 Fig. The pooled data of MFI from three separate experiments are plotted as bar graphs. Data are presented as fold change relative to control BMDCs (BMDCs that were left untransfected, uninfected and cultured without LPS or TNFα treatment). Error bars represent SD. Each symbol represents data of individual experiment. *p < 0.001, **p < 0.01, ***p < 0.05; NS, not significant.
(TIF)

S13 Fig Silencing of SHP-1 does not alter TLR4, TNFR1 and TNFR2 expression on DCs. BMDCs were either left untransfected (- siRNA) or transfected with control (Ctrl) siRNA or SHP-1-specific siRNA. The expression of TLR4, TNFR1 and TNFR2 expression on BMDCs was analyzed by flow cytometry. Data are representative of two independent experiments.
(TIF)

S14 Fig MFI data of CD40 expression and densitometry results related to Fig 7. (A) Relates to Fig 7A. Bar graphs show pooled densitometry data (*n* = 3 independent experiments) for EMSA (shown in Fig 7A) of Runx1 and Runx3 binding to the mouse *CD40* promoter (assessed using Pr1 and Pr2 probes) in BMDCs treated with SAG for indicated times. Densitometry analysis was performed as in Fig 7A, and presented relative to untreated BMDCs (0 h). (B) Relates to Fig 7J. The compiled data for MFI of CD40 expression from three independent experiments depicting the effect of Runx1 and/or Runx3 silencing on SAG-induced CD40 expression on BMDCs is shown in the bar diagram. The MFI values of CD40 expression were calculated as described in S1 Fig and presented as fold change relative to untransfected (- siRNA) BMDCs cultured without SAG. Error bars represent SD. Each symbol represents data of individual experiment. *p < 0.001, **p < 0.01, ***p < 0.05; NS, not significant.
(TIF)

S15 Fig MFI data of CD40 expression and densitometry results related to Fig 8. (A) Relates to Fig 8A. The bar diagram represents the data compiled from three separate experiments for MFI of CD40 expression by BMDCs left uninfected or infected with LDPm for 24 h and then cultured with or without SAG for 24 h. The MFI values were calculated as described in S1 Fig and presented as fold change relative to uninfected BMDCs cultured without SAG. (B) Relates to Fig 8D. Graphs show densitometry results pooled from three independent experiments for EMSA of Runx binding to the mouse *CD40* promoter using Pr1 and Pr2 probes (left two panels) and immunoblot analysis of Runx1 and Runx3 expression (right two panels) in BMDCs that had been left uninfected or infected with LDPm for 24 h, then cultured with or without SAG for 0.5 h. Densitometry analysis was done as in Fig 8D, and presented relative to uninfected BMDCs cultured without SAG. Error bars represent SD. Each symbol represents data of individual experiment. *p < 0.001, **p < 0.01; NS, not significant.
(TIF)

S16 Fig SAG treatment for 0.3 or 1 h does not reduce parasite load in LD-infected DCs. BMDCs were infected with LDPm for 24 h and then treated with SAG for 0.3 or 1 h, or left untreated (0 h). The percentage of infected BMDCs (A) and the number of intracellular amastigotes per 1000 BMDCs (B) were determined by Giemsa staining. Data are a compilation of two separate experiments (*n* = 3 in each experiment). The horizontal bars represent the mean. Each symbol represents data of individual replicate. In these experiments, LD strain AG83 was used. NS, not significant.
(TIF)

S17 Fig Analysis of CD40 expression on SAG-treated DCs after Runx1 and Runx3 silencing and CD40 overexpression. Relates to Figs 9 and 10. BALB/c BMDCs were treated with PBS or SAG for 24 h. In some experiments, prior to SAG treatment, BMDCs were transfected with control siRNA, or with Runx1 and Runx3 siRNAs alone or together with an empty vector (Vec) or CD40-expressing vector (CD40). The expression of CD40 on BMDCs was measured by flow cytometry. Data are representative of two independent experiments.
(TIF)

S18 Fig SAG-induced Runx-mediated CD40 upregulation on DCs is necessary for reduction of hepatosplenomegaly in LD-infected mice. Relates to Fig 9B. Representative pictures of spleens and livers from LD-infected (Inf) mice and age-matched uninfected (Uninf) mice after adoptive transfer of DCs treated as in Fig 9.
(TIF)

S19 Fig Gating strategy for analysis of IFNγ- and IL-10-producing splenic CD4^+^ and CD8^+^ T cells. Relates to Fig 10. Total lymphocytes were first gated based on forward and side scatter, followed by CD3 expression. The gated CD3^+^ cells were then analyzed for CD4^+^, CD8^+^, IFNγ^+^ and/or IL-10^+^ population (Fig 10) based on background staining with isotype control antibodies [rat IgG-FITC for anti-CD4-FITC (A) or anti-CD8-FITC (B), and rat IgG-PE for anti-IFNγ-PE or anti-IL-10-PE].
(TIF)

S20 Fig MFI data of CD40 expression and densitometry results related to Fig 11. (A) Relates to Fig 11B. The bar diagram represents the data compiled from three separate experiments for MFI of CD40 expression by BMDCs left uninfected or infected with promastigotes of Sb^R^LD strain GE1F8R or Sb^S^LD strain AG83 for 24 h and then cultured with or without SAG for 24 h. The MFI values were calculated as described in S1 Fig and presented as fold change relative to uninfected BMDCs cultured without SAG. (B) Relates to Fig 11G. A compilation of densitometry results from three separate experiments for EMSA of Runx1 and Runx3 binding to the mouse *CD40* promoter-specific Pr1 and Pr2 probes (left two panels), and immunoblot analysis of Runx1 and Runx3 expression (right two panels) in BMDCs left uninfected or infected with Sb^S^LD strain AG83 or Sb^R^LD strain GE1F8R for 24 h and then cultured (for 0.5 h) with or without SAG. Densitometry analysis was performed as in Fig 11G and presented relative to uninfected BMDCs cultured without SAG. Error bars represent SD. Each symbol represents data of individual experiment. *p < 0.001, **p < 0.01, ***p < 0.05; NS, not significant.
(TIF)

S1 Table Details of primers and oligonucleotides used for *in vivo* footprint analysis and EMSA. (DOCX)
