# Supplementary figures and images for: Runx proteins mediate protective immunity against Leishmania donovani infection by promoting CD40 expression on dendritic cells
Source: PLoS Pathog. 2020 Dec 28;16(12):e1009136. doi: 10.1371/journal.ppat.1009136 (PMC7793297; doi:10.1371/journal.ppat.1009136)

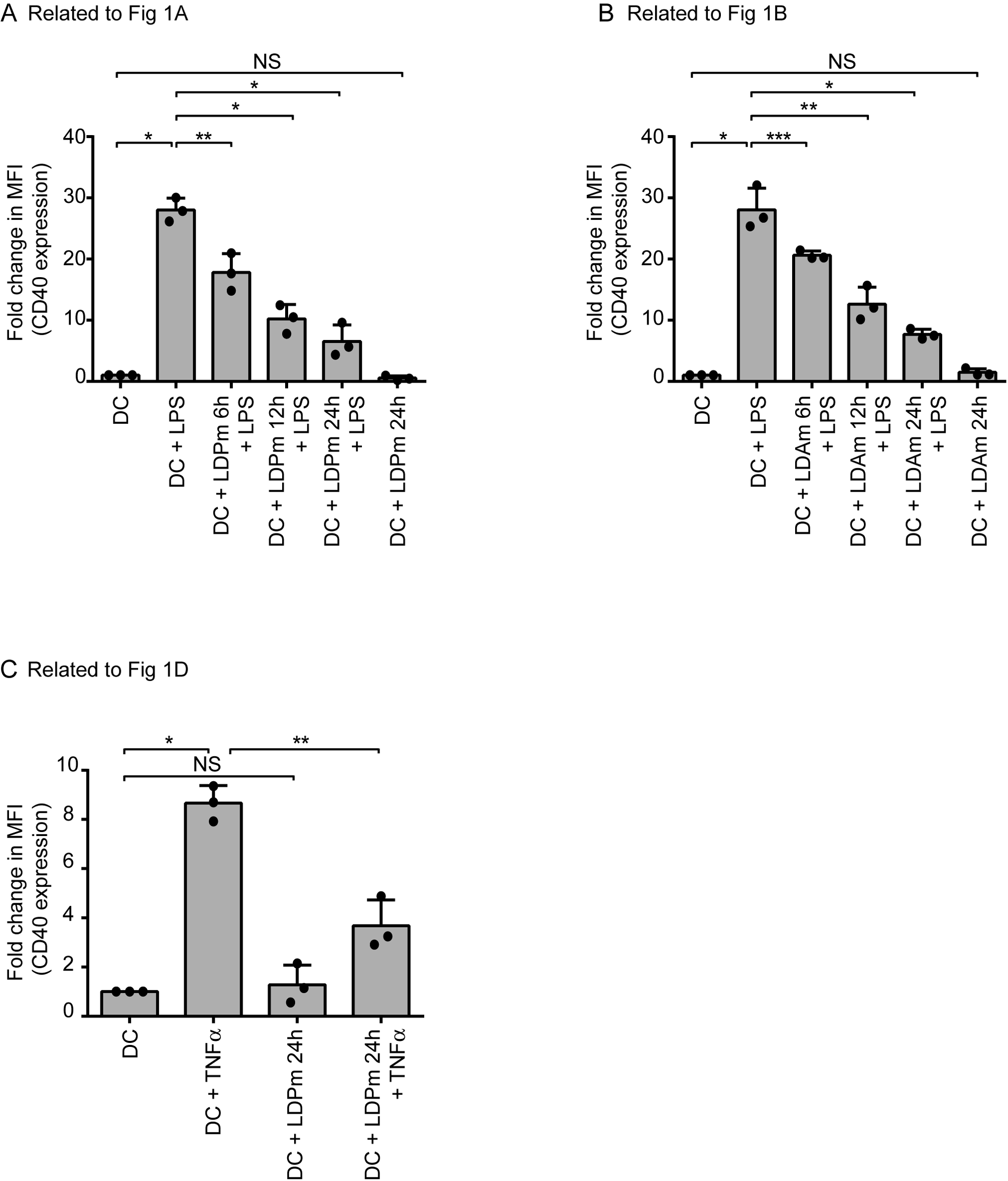

Supplement: S1 Fig — Relates to Fig 1A, 1B and 1D. BMDCs were either left uninfected or infected with LDPm (A; relates to Fig 1A) or LDAm (B; relates to Fig 1B) for indicated times and then stimulated with LPS for 24 h or left unstimulated. In some experiments (C; relates to Fig 1D), BMDCs were infected for 24 h with LDPm and stimulated with TNFα for 24 h. The expression of CD40 was analyzed by flow cytometry (shown in Fig 1A, 1B and 1D). The mean fluorescence intensity (MFI) of corresponding CD40 expression was calculated after subtracting isotype background and is presented here as fold change relative to control DCs (DCs; i.e., DCs left uninfected and unstimulated). Data are a compilation of three separate experiments. Error bars represent SD. Each symbol represents data of individual experiment. *p < 0.001, **p < 0.01, ***p < 0.05; NS, not significant. (TIF) [file ppat.1009136.s002.tif]

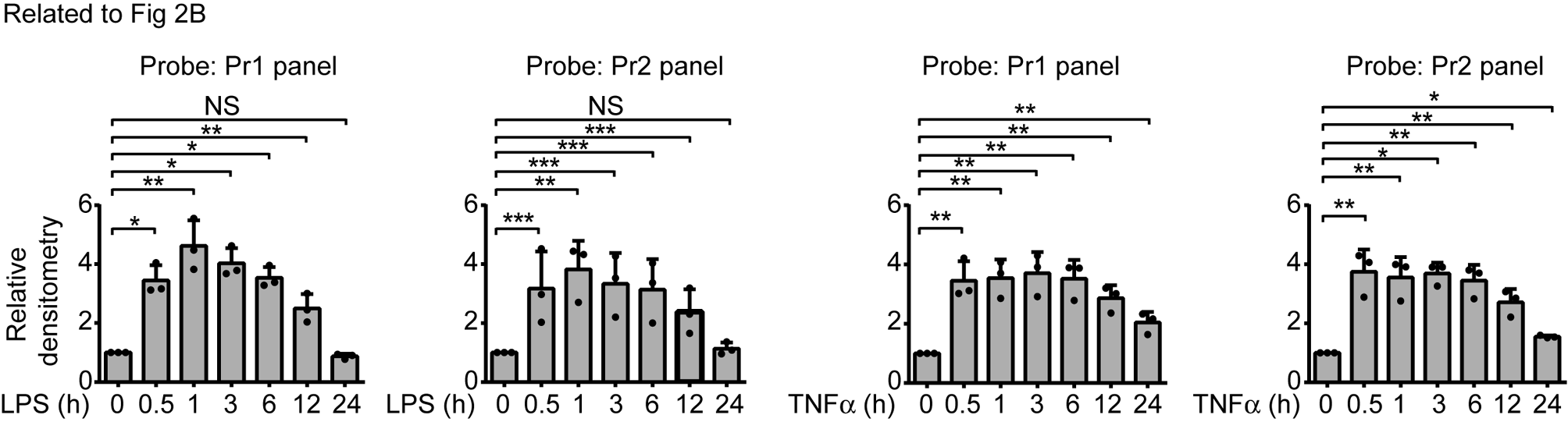

Supplement: S2 Fig — Relates to Fig 2B. BMDCs were treated with LPS or TNFα for indicated times. The binding of nuclear proteins to mouse CD40 promoter-specific probes (Pr1 and Pr2) that contained putative Runx-binding sites was determined via EMSA (shown in Fig 2B). Bar graphs here show pooled densitometry results (n = 3 independent experiments) for intensity of nuclear protein binding to indicated probes. Densitometry analysis was performed as in Fig 2B and data are presented relative to untreated BMDCs (0 h). Each symbol represents data of individual experiment. *p < 0.001, **p < 0.01, ***p < 0.05; NS, not significant. (TIF) [file ppat.1009136.s003.tif]

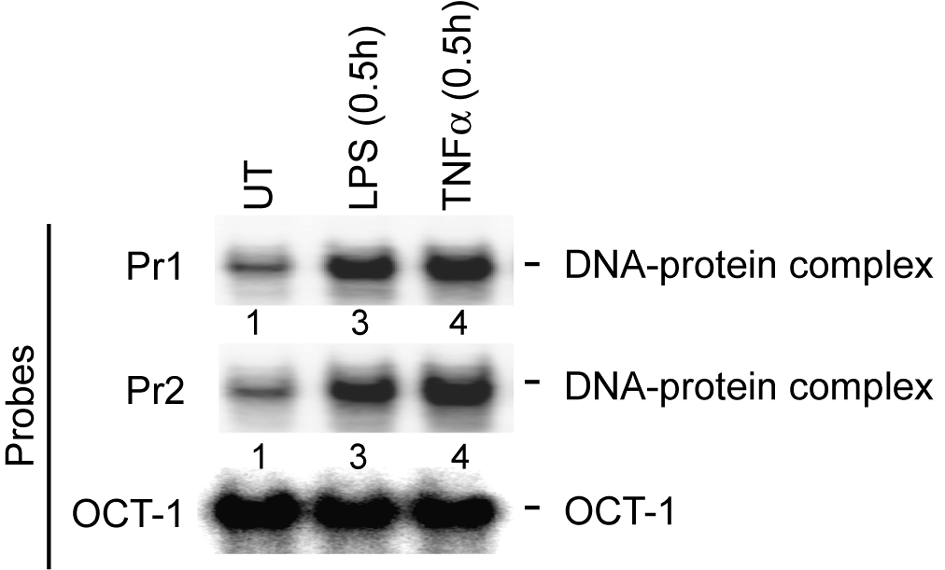

Supplement: S3 Fig — BALB/c sDCs were treated with LPS or TNFα for 0.5 h or left untreated. Nuclear extracts were subjected to EMSA using indicated probes (as in Fig 2B). Numbers below lanes represent densitometry [normalized to OCT-1 binding (control)] relative to that of untreated (UT) sDCs. Data are representative of three independent experiments. (TIF) [file ppat.1009136.s004.tif]

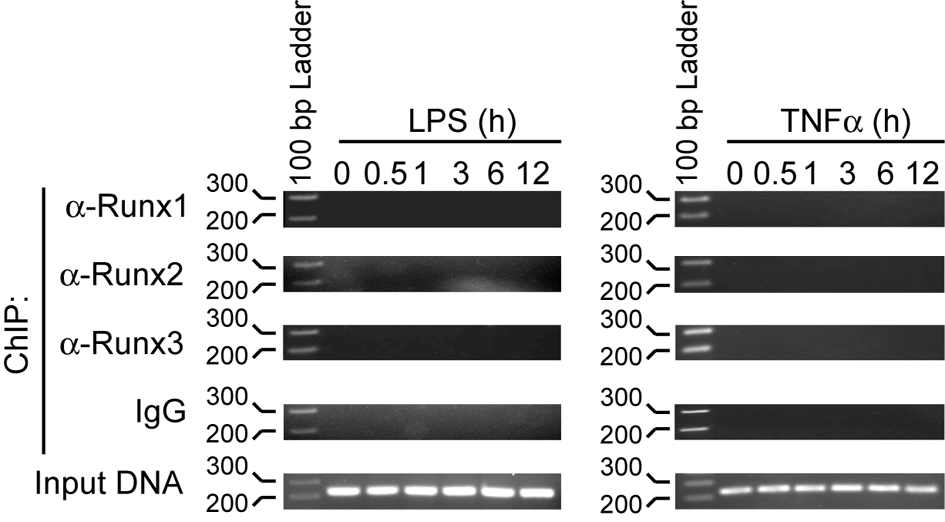

Supplement: S4 Fig — Relates to Fig 2E. BMDCs were treated with LPS or TNFα for indicated times. The recruitment of Runx proteins to the mouse GAPDH promoter was examined by ChIP using indicated antibodies (left margin) and the primers described in “Materials and methods”. Amplification of chromatin immunoprecipitated by rabbit IgG served as a negative control, and input DNA (2%) as an internal control. Data are representative of three independent experiments. (TIF) [file ppat.1009136.s005.tif]

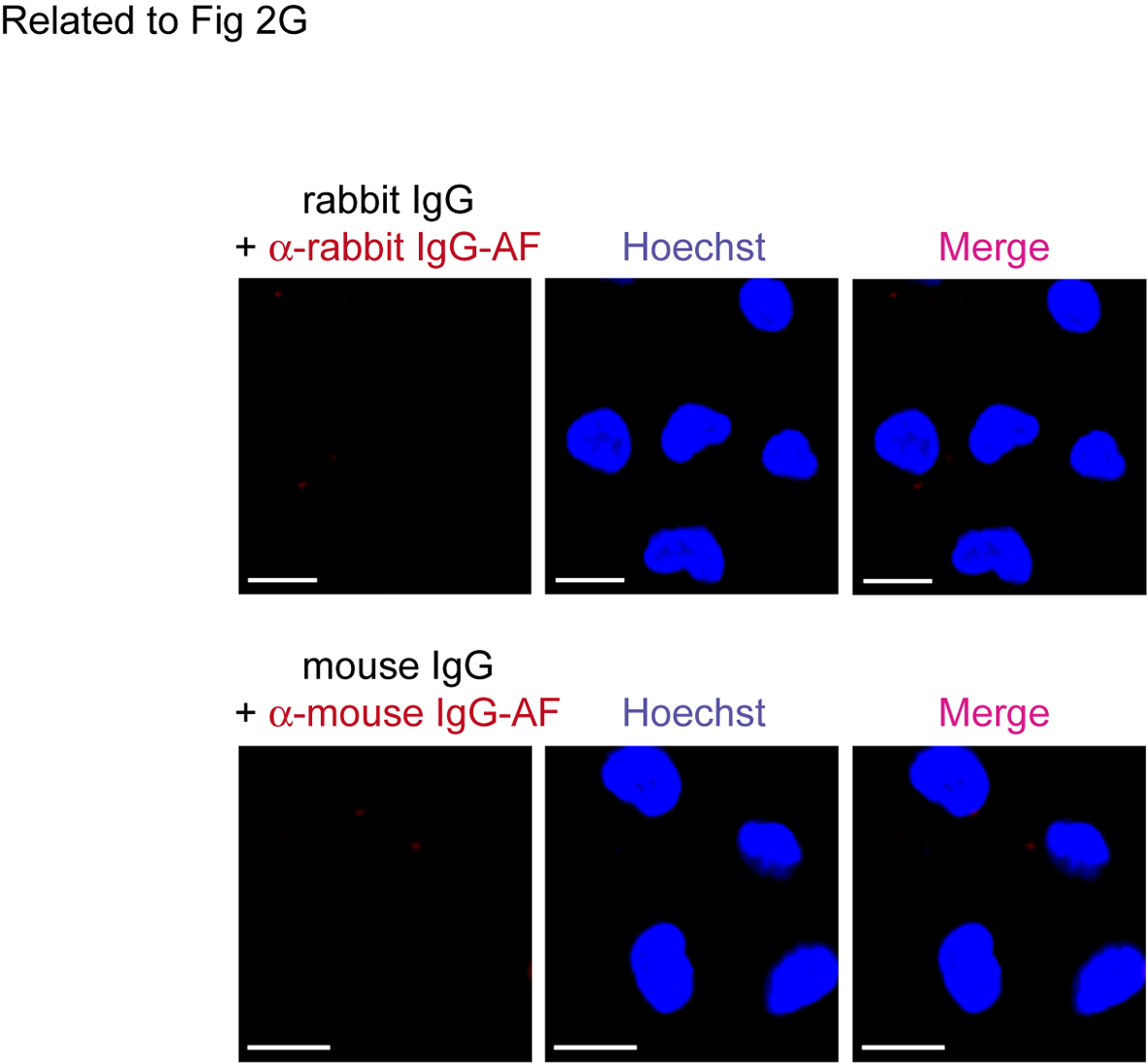

Supplement: S5 Fig — Relates to Fig 2G. BMDCs were stained with Hoechst (blue; stains all nuclei) and immunolabeled with rabbit IgG (isotype control for anti-Runx1) or mouse IgG (isotype control for anti-Runx3), followed by Alexa Fluor 568-conjugated goat anti-rabbit IgG or goat anti-mouse IgG (red) secondary antibody, respectively. Cells were analyzed via confocal microscopy. The lack of red fluorescence within cytoplasm and/or nuclei (“rabbit IgG + anti-rabbit IgG-AF” and “mouse IgG + anti-mouse IgG-AF” panels), and the absence of pink color within nuclei in merged images (“merge” panels) confirm the specificity of anti-Runx1 and anti-Runx3 antibodies used in Fig 2G and all other experiments. Scale bar, 10 μm. AF, Alexa Fluor 568. Data are representative of two independent experiments. (TIF) [file ppat.1009136.s006.tif]

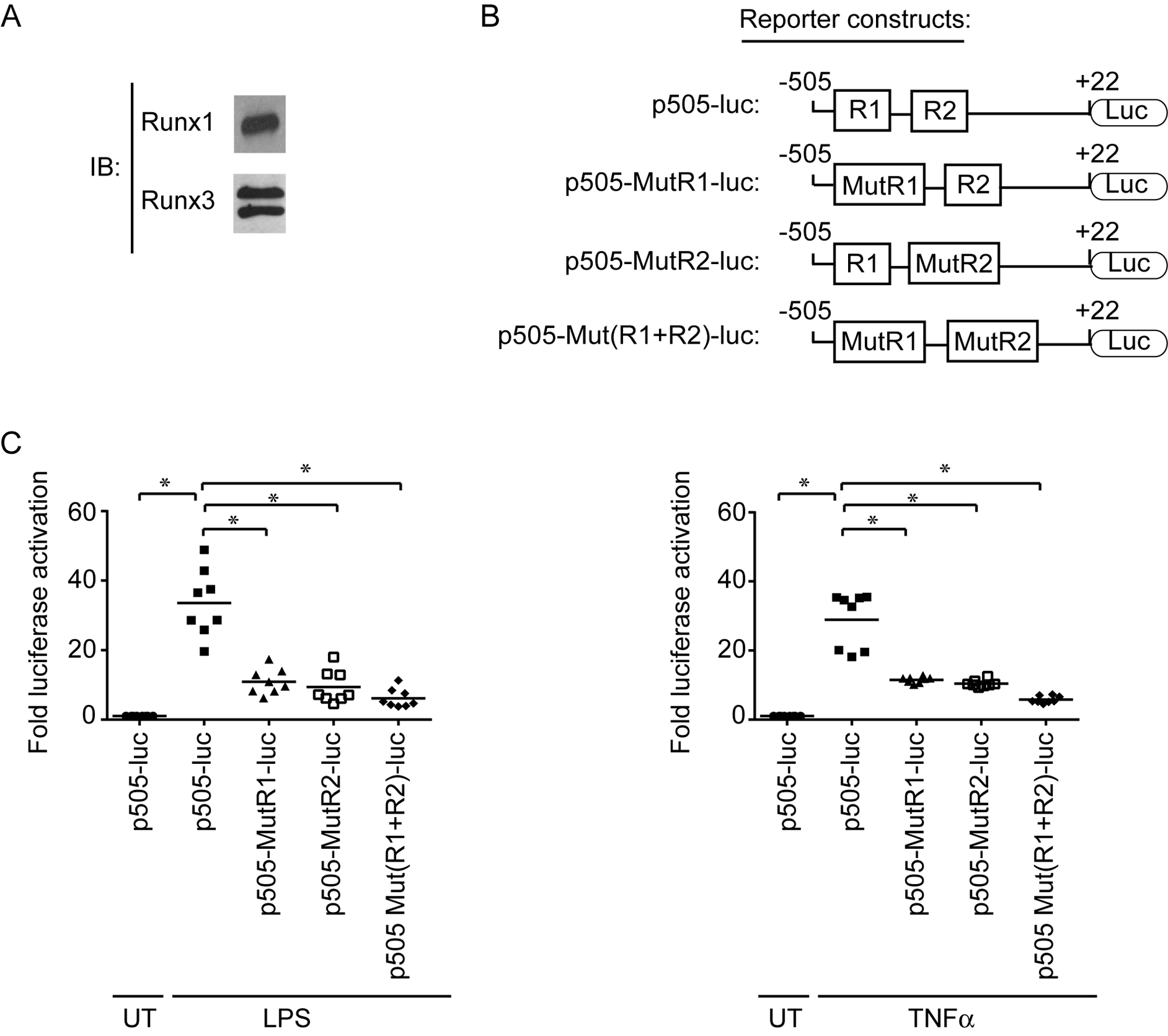

Supplement: S6 Fig — (A) Immunoblot analysis of Runx1 and Runx3 in JAWSII cell lysates. (B) Schematic of the mouse CD40 promoter-firefly luciferase (luc) reporter constructs used in reporter assay. Expression of firefly luciferase is controlled by wild-type mouse CD40 promoter fragment (-505 to +22 region; p505-luc) or similar CD40 promoter fragment carrying mutations in either of the Runx-binding sites (p505-MutR1-luc or p505-MutR2-luc) or both Runx-binding sites [p505-Mut(R1+R2)-luc]. (C) Dual-luciferase assay of JAWSII cells that had been transfected with indicated CD40 promoter-luciferase constructs (as in B) together with the renilla luciferase plasmid pRL-CMV (internal control) and then treated for 24 h with LPS (left panel) or TNFα (right panel) or left untreated (UT). Results were normalized to the activity of renilla luciferase and are presented relative to those in p505-luc-transfected JAWSII cells that had been left untreated. Horizontal bars represent the mean. Data are a compilation of two separate experiments (n = 5 in one experiment, and n = 3 in another experiment). Each symbol represents data of individual replicate. *p < 0.001. (TIF) [file ppat.1009136.s007.tif]

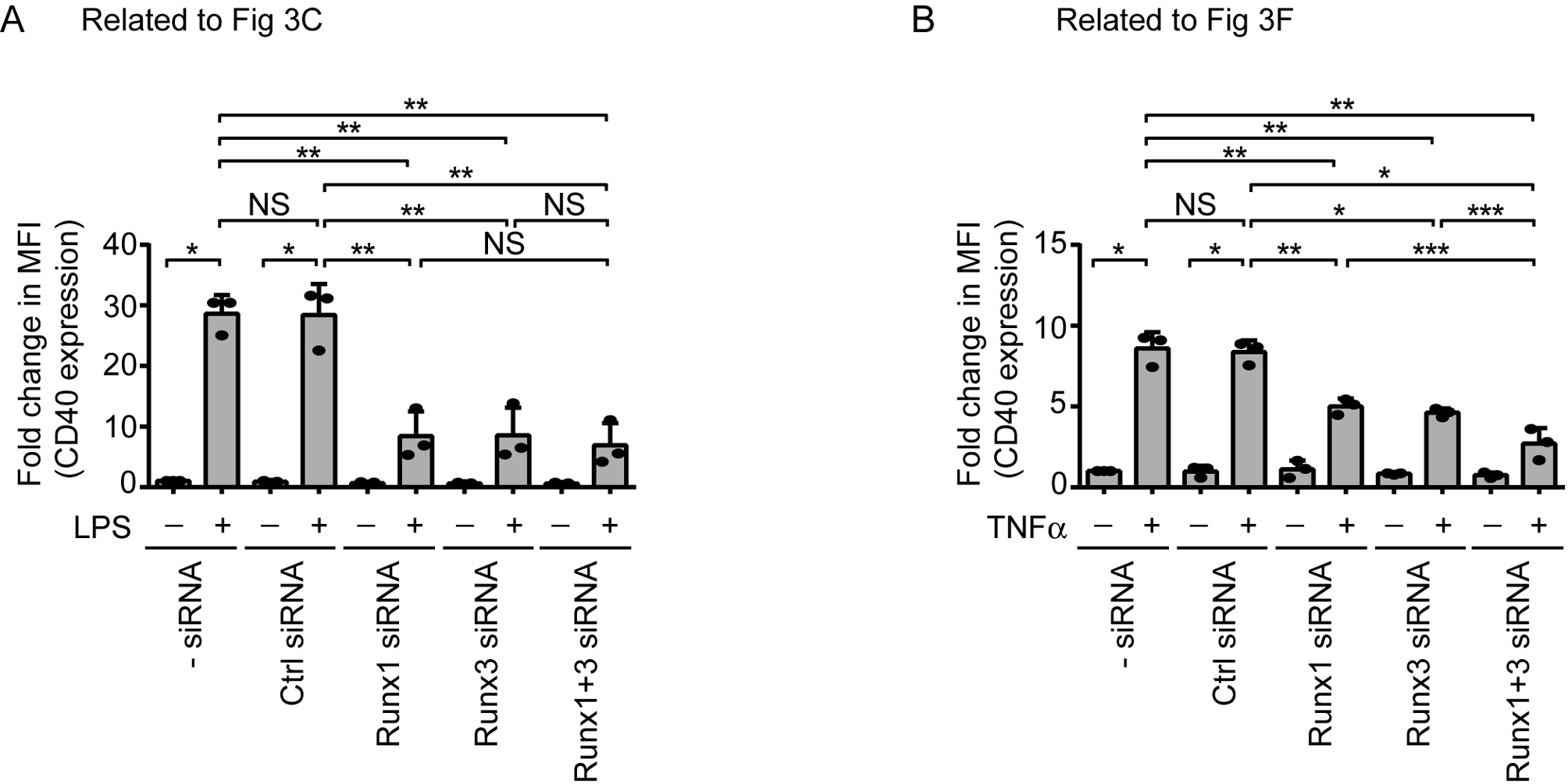

Supplement: S7 Fig — Relates to Fig 3C and 3F. BMDCs were left untransfected (- siRNA) or transfected with control (Ctrl) siRNA, Runx1 siRNA, Runx3 siRNA or Runx1 and Runx3 siRNAs (Runx1+3 siRNA) and then cultured with (+) or without (-) LPS for 24 h (A; relates to Fig 3C). In some experiments (B; relates to Fig 3F), BMDCs were transfected with siRNAs as described above, and then cultured with (+) or without (-) TNFα for 24 h. The expression of CD40 on BMDCs assessed by flow cytometry has been shown in Fig 3C and 3F. Corresponding MFI data of CD40 expression (assessed as in S1 Fig) pooled from three independent experiments are shown in bar graphs, and presented as fold change relative to BMDCs left untransfected and cultured without LPS (A) or TNFα (B). Error bars represent SD. Each symbol represents data of individual experiment. *p < 0.001, **p < 0.01, ***p < 0.05; NS, not significant. (TIF) [file ppat.1009136.s008.tif]

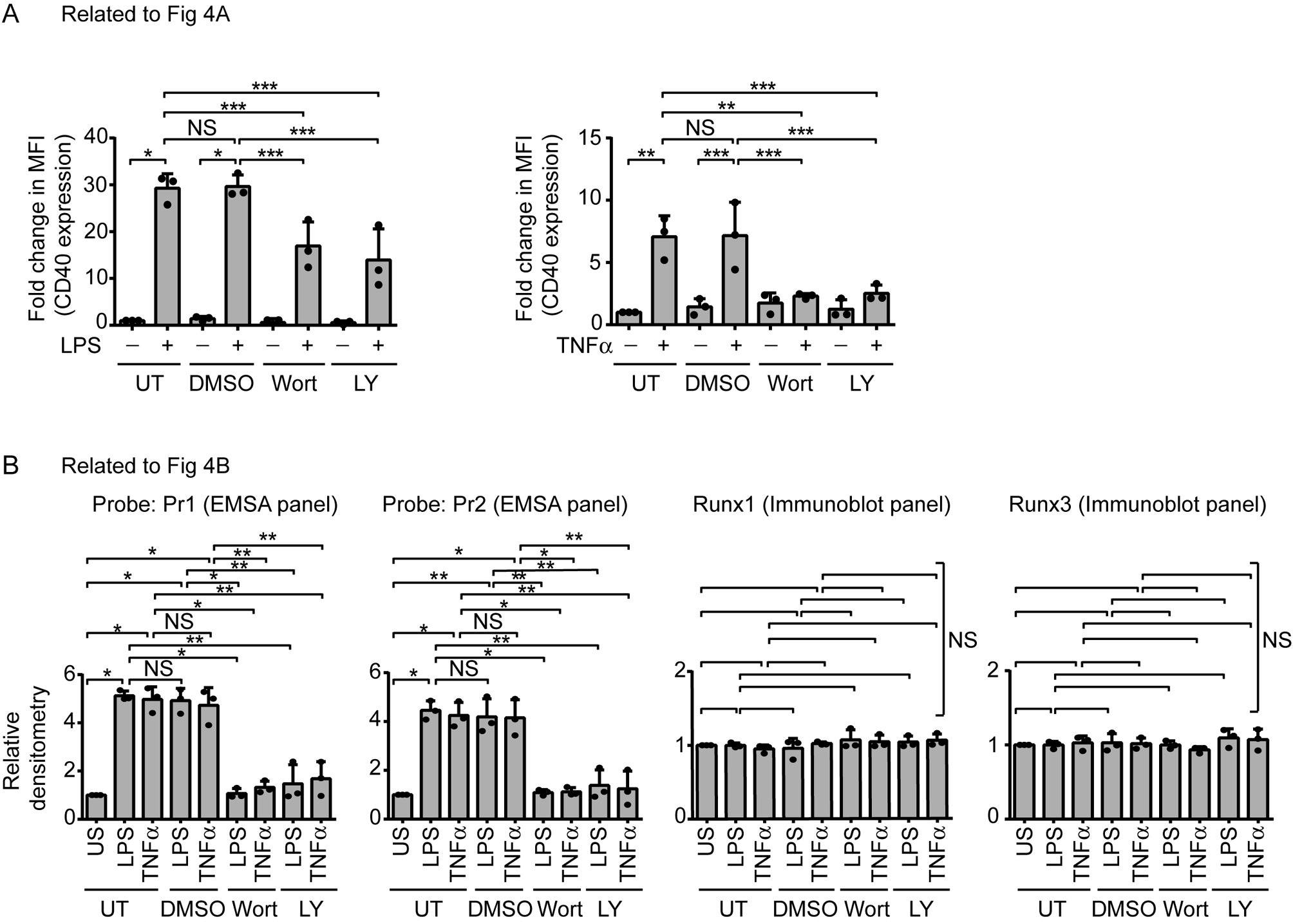

Supplement: S8 Fig — (A) Relates to Fig 4A. BMDCs were left untreated (UT) or treated with DMSO (0.1%; control treatment), Wort or LY for 1 h and then cultured for 24 h with (+) or without (-) LPS (left panel) or TNFα (right panel). The expression of CD40 on BMDCs assessed by flow cytometry has been shown in Fig 4A. The MFI data of CD40 expression (assessed as in S1 Fig) for Fig 4A pooled from three independent experiments are depicted in bar graphs, and presented as fold change relative to untreated BMDCs cultured without LPS (left panel) or TNFα (right panel). Error bars represent SD. Each symbol represents data of individual experiment. (B) Relates to Fig 4B. BMDCs were left untreated or treated for 1 h with DMSO, Wort or LY and then left unstimulated (US) or stimulated with LPS or TNFα for 0.5 h. The EMSA and immunoblot data have been shown in Fig 4B. Here, bar graphs depict pooled densitometry results (n = 3 independent experiments) for Fig 4B measuring the intensity of Runx1 and Runx3 binding to the mouse CD40 promoter-specific probes (Pr1 and Pr2; left two panels), and the expression of Runx1 and Runx3 in BMDC lysates (right two panels). Data are presented relative to untreated BMDCs that had been left unstimulated. Error bars represent SD. Each symbol represents data of individual experiment. *p < 0.001, **p < 0.01, ***p < 0.05; NS, not significant. (TIF) [file ppat.1009136.s009.tif]

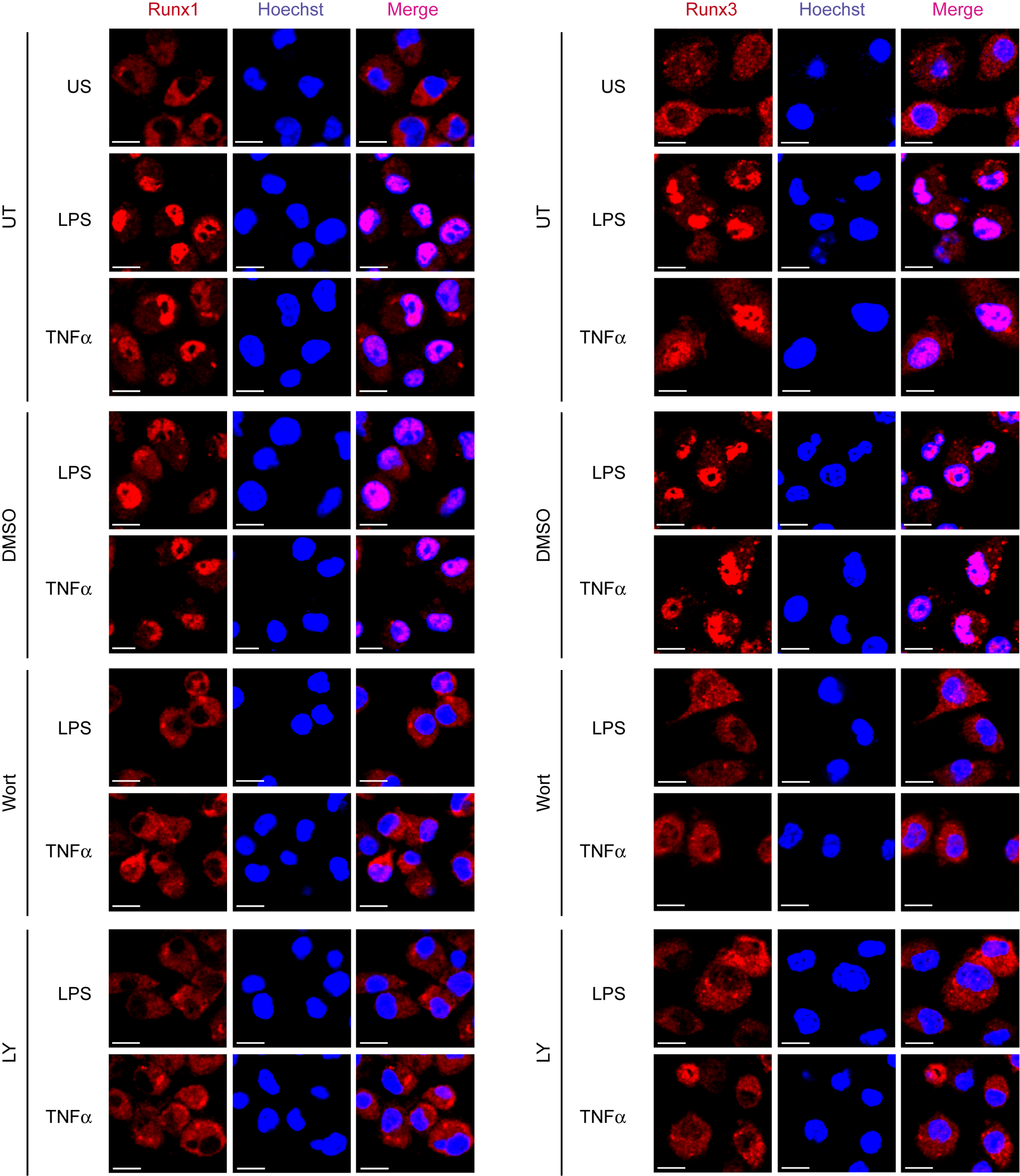

Supplement: S9 Fig — BMDCs were either left untreated (UT) or treated for 1 h with DMSO, Wort or LY and then stimulated with LPS or TNFα for 0.5 h or left unstimulated (US). The translocation of Runx1 and Runx3 (red) to the nuclei (blue) was analyzed via confocal microscopy. Pink color (merge) shows nuclear translocation of Runx1 or Runx3. Scale bar, 10 μm. Data are representative of two independent experiments. (TIF) [file ppat.1009136.s010.tif]

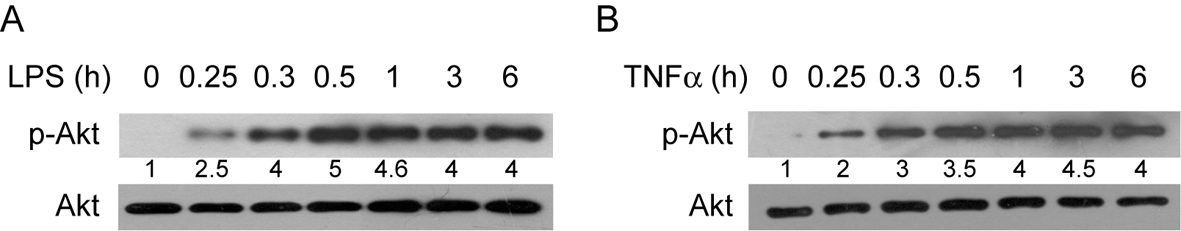

Supplement: S10 Fig — Immunoblot analysis of total and phosphorylated Akt in lysates of BMDCs treated with LPS (A) and TNFα (B) for indicated times. Numbers below lanes represent densitometry, normalized to total Akt and presented relative to untreated BMDCs (0 h). Data are representative of two independent experiments. (TIF) [file ppat.1009136.s011.tif]

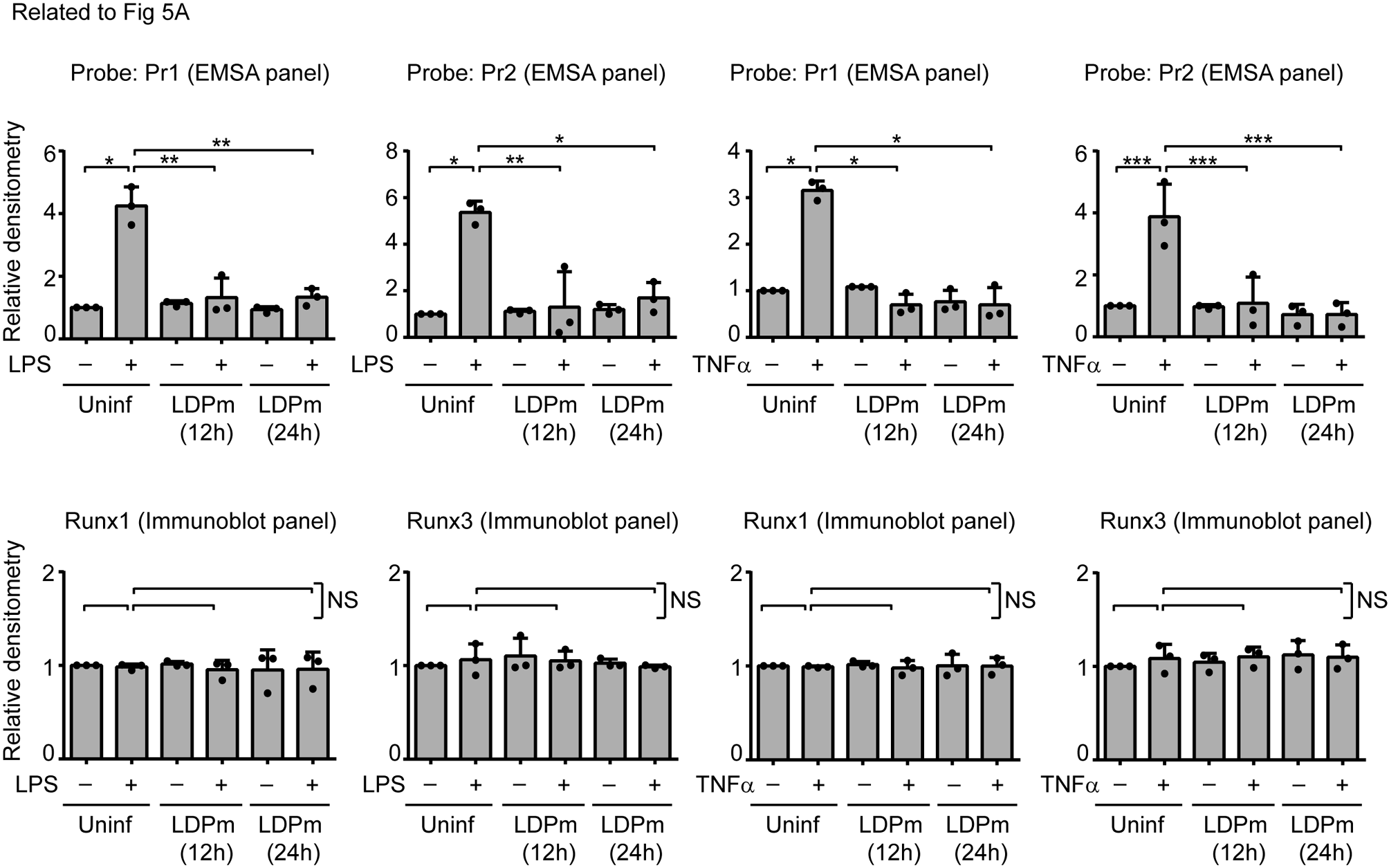

Supplement: S11 Fig — BMDCs were left uninfected (Uninf) or infected with LDPm for indicated times and then stimulated with (+) LPS or TNFα for 0.5 h or left unstimulated (-). The binding of nuclear Runx1 and Runx3 to the CD40 promoter (analyzed via EMSA using Pr1 and Pr2 probes) and the expression of Runx1 and Runx3 in BMDC lysates (determined by immunoblot analysis) have been shown in Fig 5A. Here, the bar graphs show corresponding densitometry results pooled from three independent experiments. Data are presented relative to uninfected BMDCs that had been left unstimulated. Error bars represent SD. Each symbol represents data of individual experiment. *p < 0.001, **p < 0.01, ***p < 0.05; NS, not significant. (TIF) [file ppat.1009136.s012.tif]

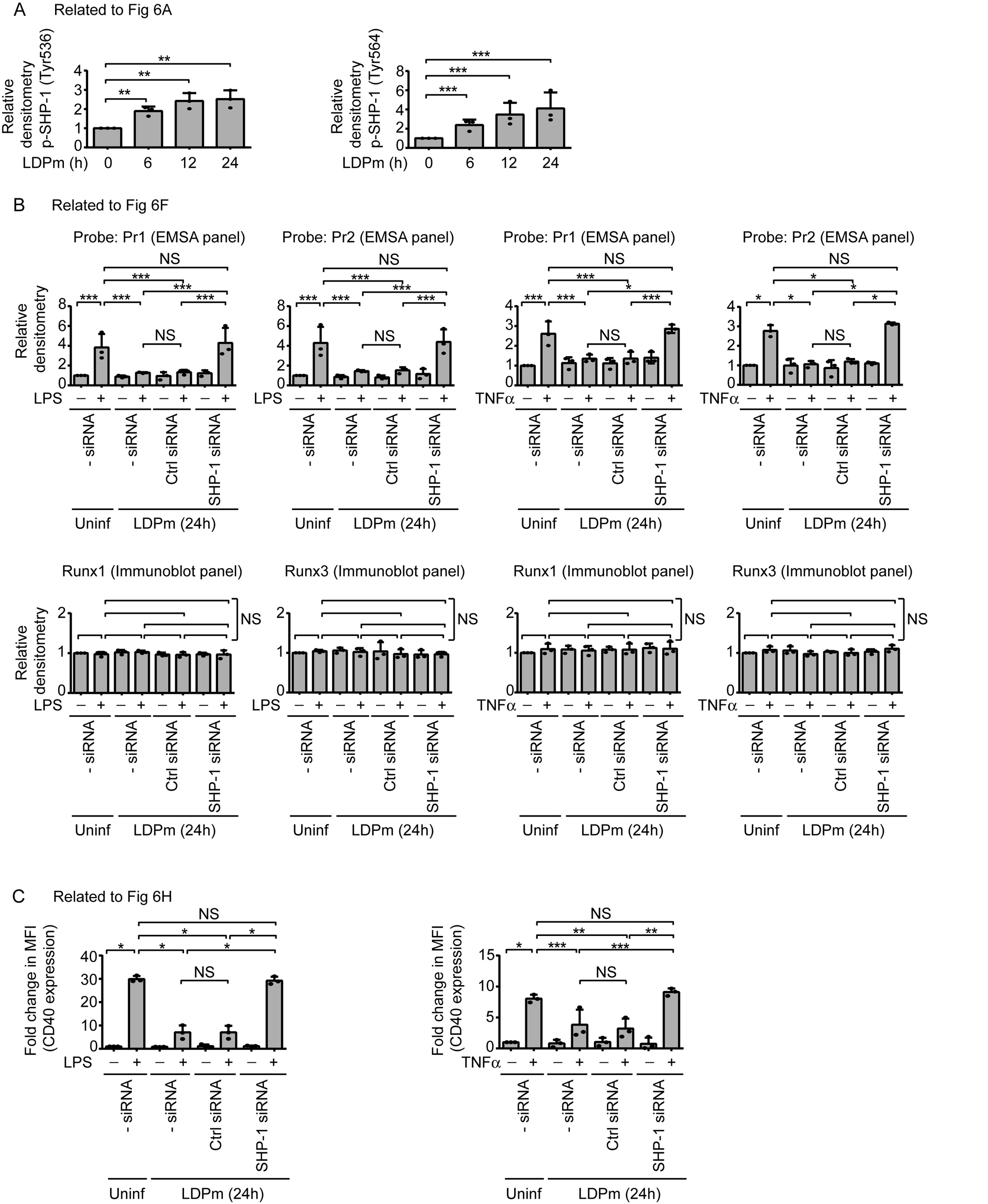

Supplement: S12 Fig — (A) Relates to Fig 6A. The bar graphs represent pooled densitometry results (n = 3 independent experiments) for immunoblot analysis (shown in Fig 6A) of SHP-1 phosphorylated at Tyr536 or Tyr564 in BMDCs infected with LDPm for indicated times. For additional details related to densitometry analysis, see Fig 6A. Data are presented relative to uninfected BMDC (0 h). (B) Relates to Fig 6F. BMDCs were transfected with indicated siRNAs, infected with LDPm for 24 h and then treated with or without LPS or TNFα for 0.5 h. The EMSA and immunoblot analysis data have been shown in Fig 6F. Here, bar graphs show corresponding densitometry results (pooled from three independent experiments) for Runx1 and Runx3 binding to the mouse CD40 promoter-specific probes (Pr1 and Pr2; upper panels), and the expression of Runx1 and Runx3 in BMDC lysates (lower panels). Densitometry analysis was carried out as described in Fig 6F. Data are presented relative to control BMDCs (BMDCs left untransfected and uninfected, and given no LPS or TNFα treatment). (C) Relates to Fig 6H. BMDCs were transfected with indicated siRNAs, then infected with LDPm for 24 h and cultured with (+) or without (-) LPS (left panel) or TNFα (right panel) for 24 h. The expression of CD40 on BMDCs (assessed by flow cytometry) has been shown in Fig 6H. Corresponding MFI of CD40 expression was calculated as in S1 Fig. The pooled data of MFI from three separate experiments are plotted as bar graphs. Data are presented as fold change relative to control BMDCs (BMDCs that were left untransfected, uninfected and cultured without LPS or TNFα treatment). Error bars represent SD. Each symbol represents data of individual experiment. *p < 0.001, **p < 0.01, ***p < 0.05; NS, not significant. (TIF) [file ppat.1009136.s013.tif]

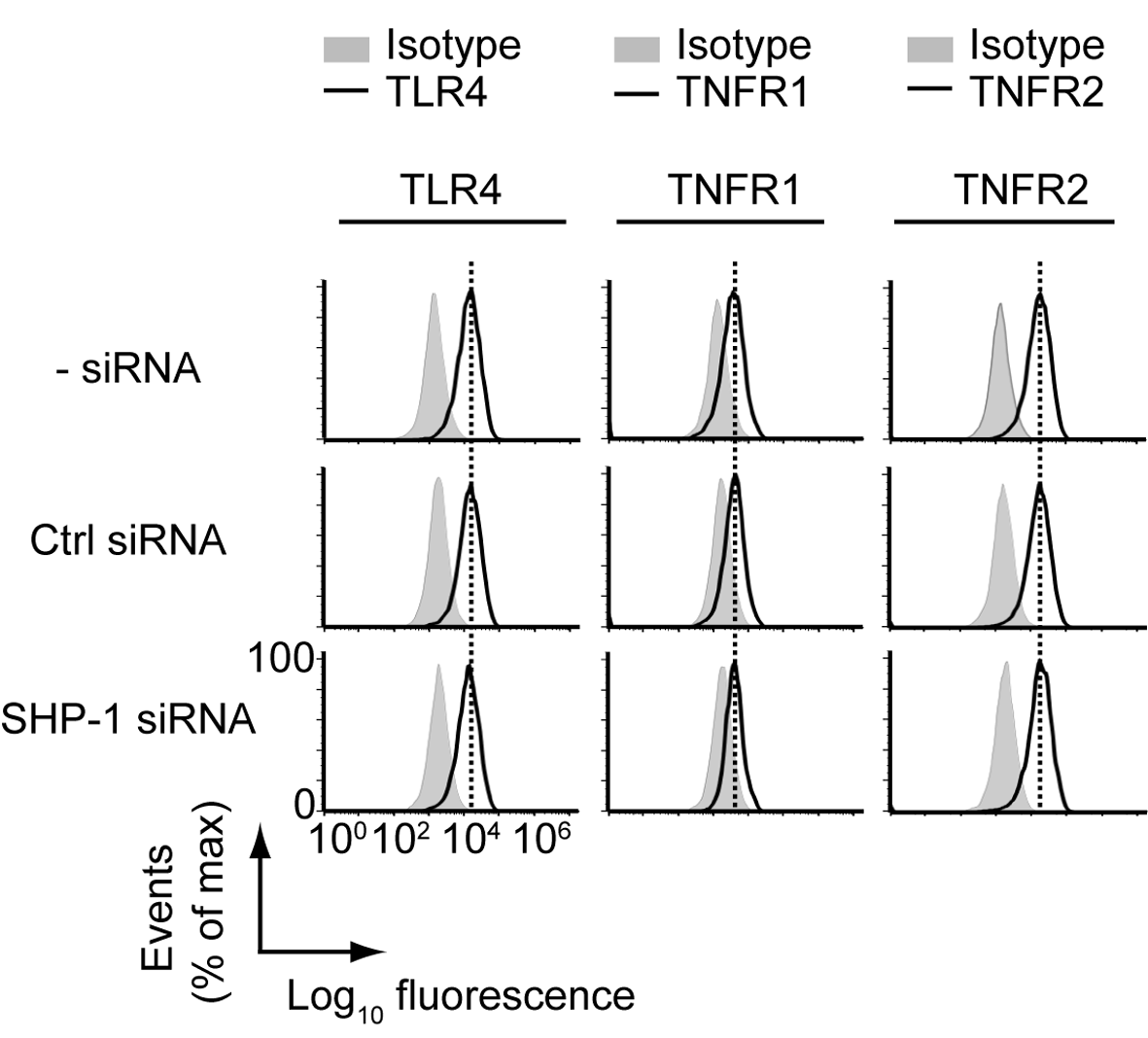

Supplement: S13 Fig — BMDCs were either left untransfected (- siRNA) or transfected with control (Ctrl) siRNA or SHP-1-specific siRNA. The expression of TLR4, TNFR1 and TNFR2 expression on BMDCs was analyzed by flow cytometry. Data are representative of two independent experiments. (TIF) [file ppat.1009136.s014.tif]

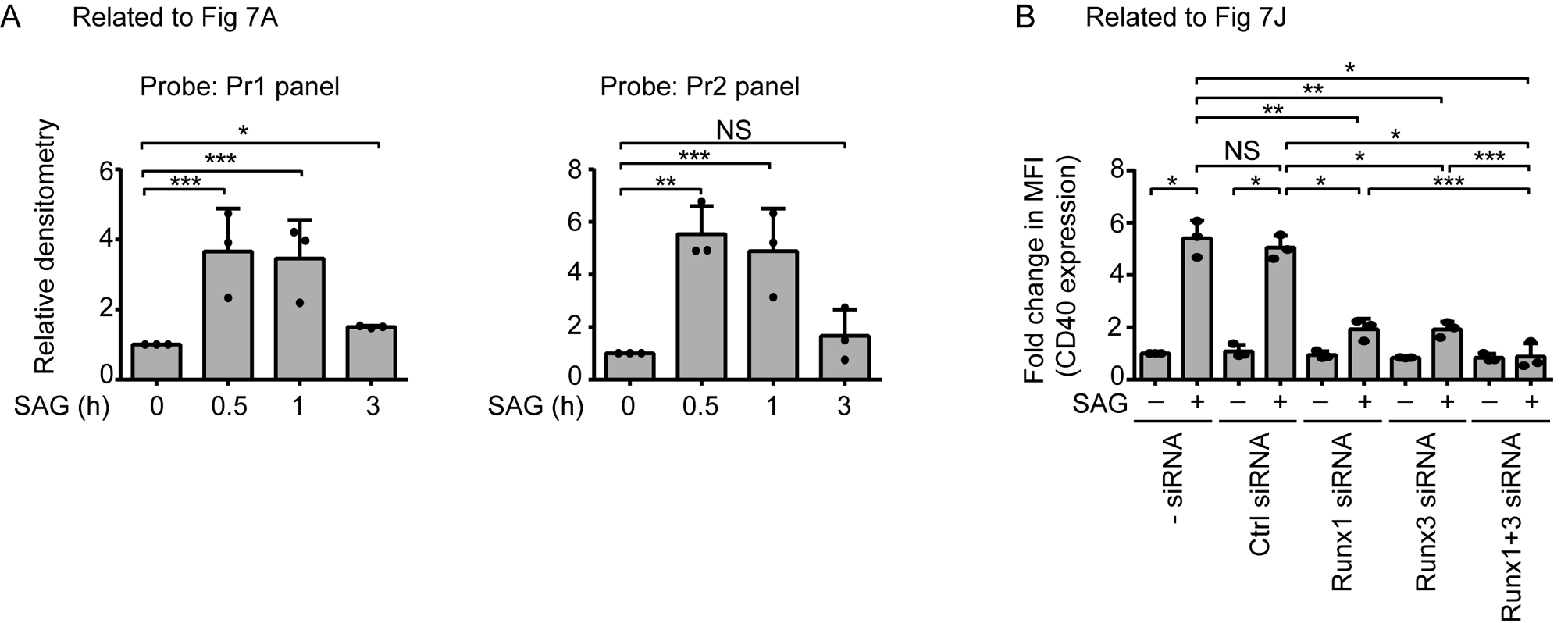

Supplement: S14 Fig — (A) Relates to Fig 7A. Bar graphs show pooled densitometry data (n = 3 independent experiments) for EMSA (shown in Fig 7A) of Runx1 and Runx3 binding to the mouse CD40 promoter (assessed using Pr1 and Pr2 probes) in BMDCs treated with SAG for indicated times. Densitometry analysis was performed as in Fig 7A, and presented relative to untreated BMDCs (0 h). (B) Relates to Fig 7J. The compiled data for MFI of CD40 expression from three independent experiments depicting the effect of Runx1 and/or Runx3 silencing on SAG-induced CD40 expression on BMDCs is shown in the bar diagram. The MFI values of CD40 expression were calculated as described in S1 Fig and presented as fold change relative to untransfected (- siRNA) BMDCs cultured without SAG. Error bars represent SD. Each symbol represents data of individual experiment. *p < 0.001, **p < 0.01, ***p < 0.05; NS, not significant. (TIF) [file ppat.1009136.s015.tif]

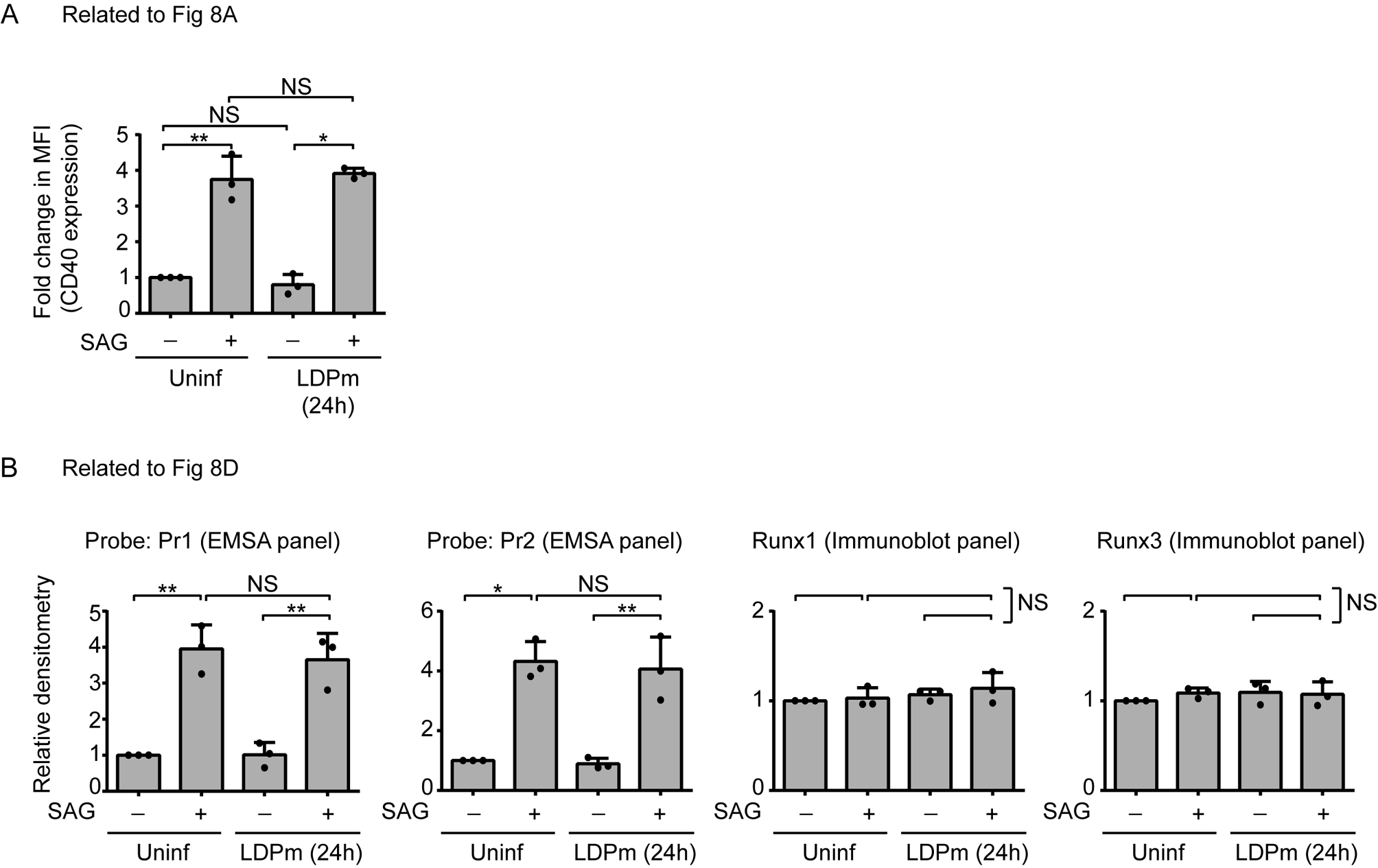

Supplement: S15 Fig — (A) Relates to Fig 8A. The bar diagram represents the data compiled from three separate experiments for MFI of CD40 expression by BMDCs left uninfected or infected with LDPm for 24 h and then cultured with or without SAG for 24 h. The MFI values were calculated as described in S1 Fig and presented as fold change relative to uninfected BMDCs cultured without SAG. (B) Relates to Fig 8D. Graphs show densitometry results pooled from three independent experiments for EMSA of Runx binding to the mouse CD40 promoter using Pr1 and Pr2 probes (left two panels) and immunoblot analysis of Runx1 and Runx3 expression (right two panels) in BMDCs that had been left uninfected or infected with LDPm for 24 h, then cultured with or without SAG for 0.5 h. Densitometry analysis was done as in Fig 8D, and presented relative to uninfected BMDCs cultured without SAG. Error bars represent SD. Each symbol represents data of individual experiment. *p < 0.001, **p < 0.01; NS, not significant. (TIF) [file ppat.1009136.s016.tif]

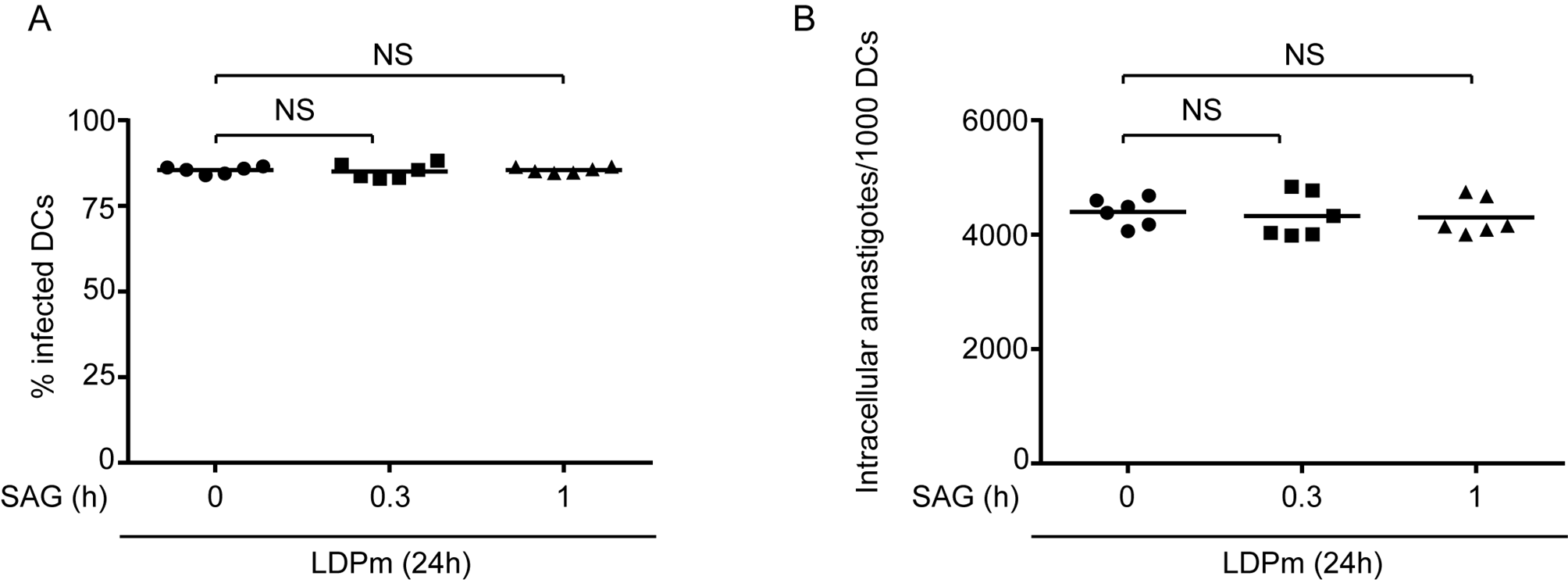

Supplement: S16 Fig — BMDCs were infected with LDPm for 24 h and then treated with SAG for 0.3 or 1 h, or left untreated (0 h). The percentage of infected BMDCs (A) and the number of intracellular amastigotes per 1000 BMDCs (B) were determined by Giemsa staining. Data are a compilation of two separate experiments (n = 3 in each experiment). The horizontal bars represent the mean. Each symbol represents data of individual replicate. In these experiments, LD strain AG83 was used. NS, not significant. (TIF) [file ppat.1009136.s017.tif]

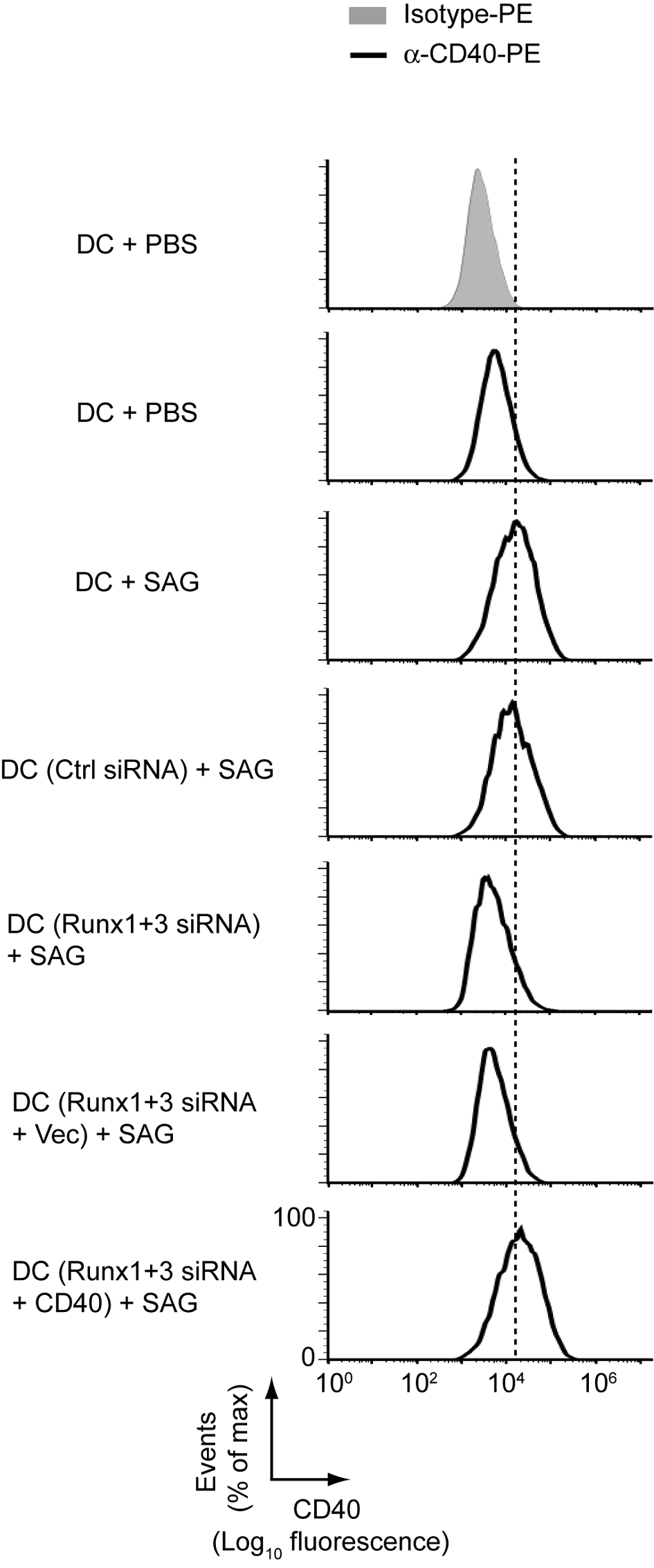

Supplement: S17 Fig — Relates to Figs 9 and 10. BALB/c BMDCs were treated with PBS or SAG for 24 h. In some experiments, prior to SAG treatment, BMDCs were transfected with control siRNA, or with Runx1 and Runx3 siRNAs alone or together with an empty vector (Vec) or CD40-expressing vector (CD40). The expression of CD40 on BMDCs was measured by flow cytometry. Data are representative of two independent experiments. (TIF) [file ppat.1009136.s018.tif]

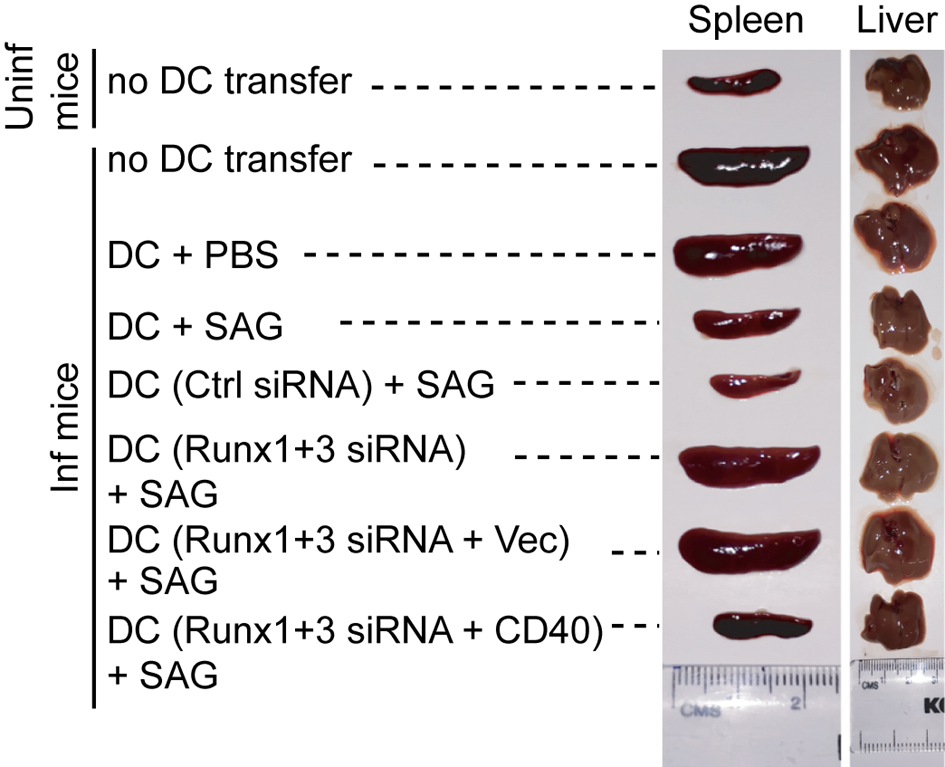

Supplement: S18 Fig — Relates to Fig 9B. Representative pictures of spleens and livers from LD-infected (Inf) mice and age-matched uninfected (Uninf) mice after adoptive transfer of DCs treated as in Fig 9. (TIF) [file ppat.1009136.s019.tif]

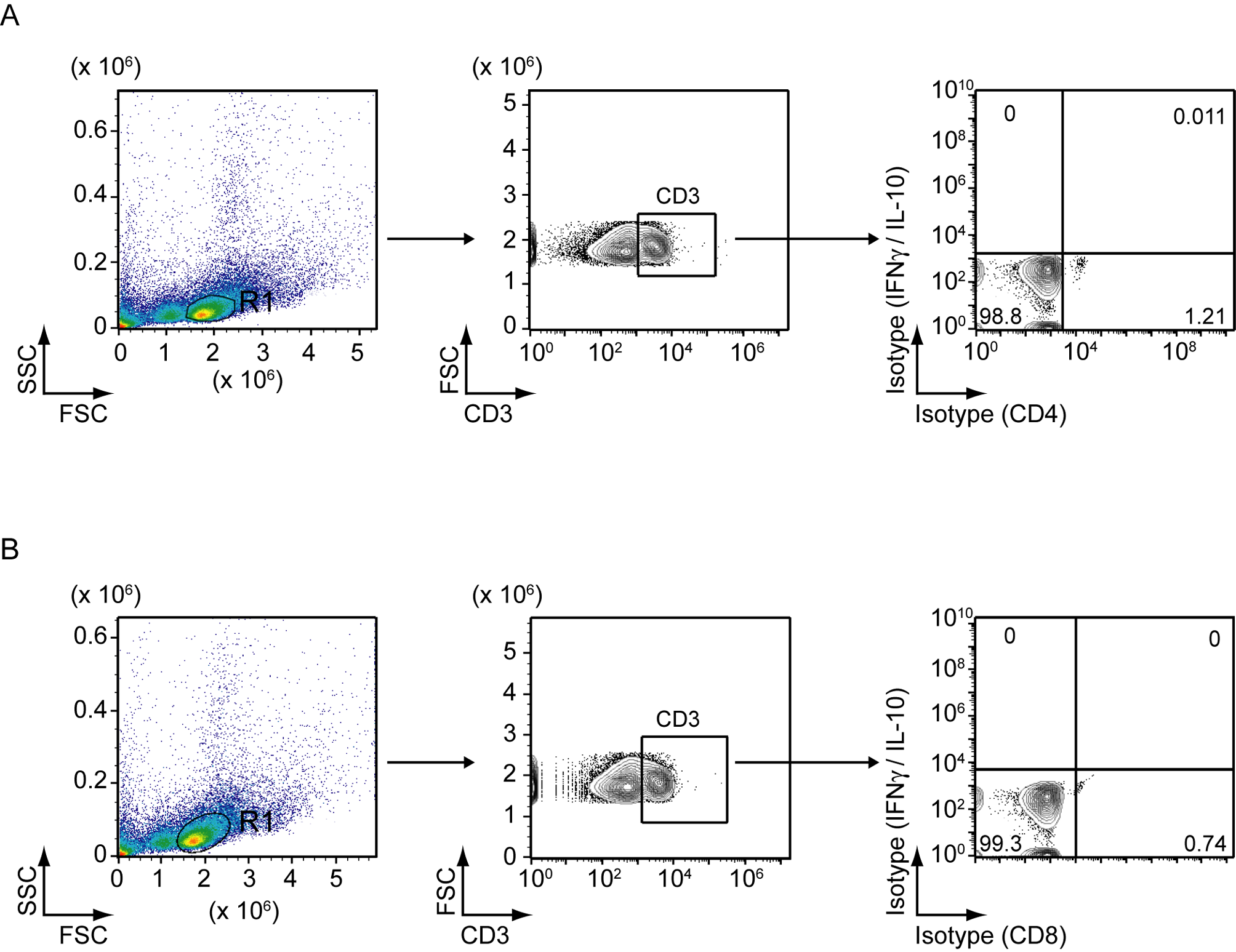

Supplement: S19 Fig — Relates to Fig 10. Total lymphocytes were first gated based on forward and side scatter, followed by CD3 expression. The gated CD3+ cells were then analyzed for CD4+, CD8+, IFNγ+ and/or IL-10+ population (Fig 10) based on background staining with isotype control antibodies [rat IgG-FITC for anti-CD4-FITC (A) or anti-CD8-FITC (B), and rat IgG-PE for anti-IFNγ-PE or anti-IL-10-PE]. (TIF) [file ppat.1009136.s020.tif]

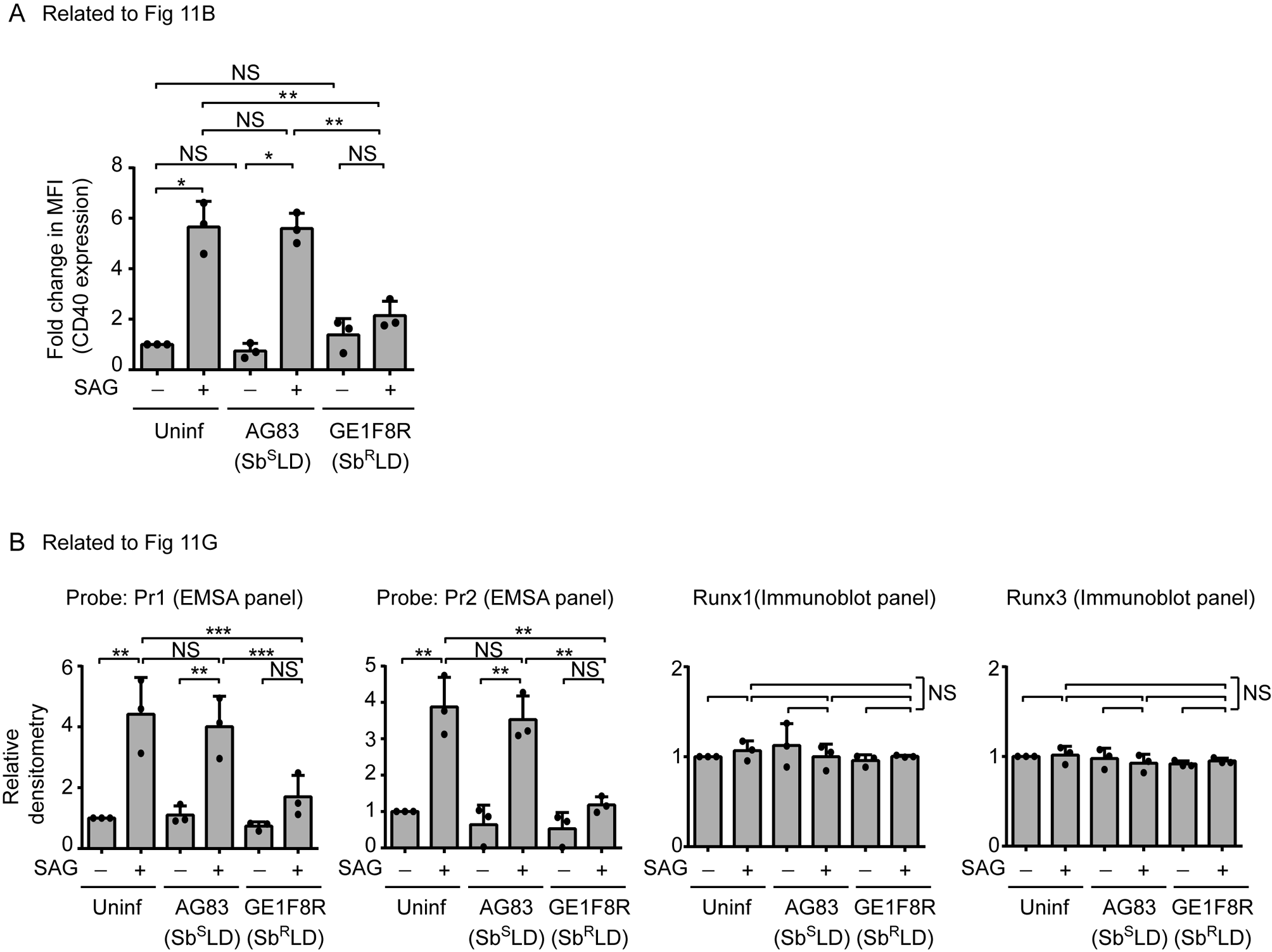

Supplement: S20 Fig — (A) Relates to Fig 11B. The bar diagram represents the data compiled from three separate experiments for MFI of CD40 expression by BMDCs left uninfected or infected with promastigotes of SbRLD strain GE1F8R or SbSLD strain AG83 for 24 h and then cultured with or without SAG for 24 h. The MFI values were calculated as described in S1 Fig and presented as fold change relative to uninfected BMDCs cultured without SAG. (B) Relates to Fig 11G. A compilation of densitometry results from three separate experiments for EMSA of Runx1 and Runx3 binding to the mouse CD40 promoter-specific Pr1 and Pr2 probes (left two panels), and immunoblot analysis of Runx1 and Runx3 expression (right two panels) in BMDCs left uninfected or infected with SbSLD strain AG83 or SbRLD strain GE1F8R for 24 h and then cultured (for 0.5 h) with or without SAG. Densitometry analysis was performed as in Fig 11G and presented relative to uninfected BMDCs cultured without SAG. Error bars represent SD. Each symbol represents data of individual experiment. *p < 0.001, **p < 0.01, ***p < 0.05; NS, not significant. (TIF) [file ppat.1009136.s021.tif]
