# Supplementary material for: Runx proteins mediate protective immunity against Leishmania donovani infection by promoting CD40 expression on dendritic cells
Source: PLoS Pathog. 2020 Dec 28;16(12):e1009136. doi: 10.1371/journal.ppat.1009136 (PMC7793297; doi:10.1371/journal.ppat.1009136)
Supplement: S1 Table — (DOCX) [file ppat.1009136.s022.docx]

Primer sequences for *in vivo* footprint analyses of the mouse *CD40* promoter

| DNA strand | Primers | Primer positions | Primer sequence (5'→3') |
| --- | --- | --- | --- |
| Coding strand | Antisense Primer 1 | -409 to -392 | aatacccctgggaatctc  (Biotinylated at 5' end) |
|  | Antisense Primer 2 | -424 to -404 | aatctctgcagaaccgaaagc |
|  | Antisense Primer 3 | -436 to -415 | aaccgaaagcgtctccagagag |

All position numbers are relative to the transcription start sites.

Probes used for EMSA

| Probes | Positions | Sequence (5'→3') |
| --- | --- | --- |
| **Mouse *CD40* promoter** |  |  |
| i) Pr1 Probe (carrying wild-type Runx site) | -501 to -465 | TGGCCCTTCAGCTGTGGTCTTTCCCGTTTTCTGACTT |
| ii) Mut-Pr1 probe (carrying mutant Runx site) | -501 to -465 | TGGCCCTTCAGC*GAATTC*CTTTCCCGTTTTCTGACTT |
| iii) Pr2 probe (carrying  wild-type Runx site) | -475 to -431 | TTTTCTGACTTTGCGGTGACACTGGGGACTTCCTTAGACCTCTCT |
| iv) Mut-Pr2 probe (carrying  mutant Runx site) | -475 to -431 | TTTTCTGACTT*GAATTC*GACACTGGGGACTTCCTTAGACCTCTCT |
|  |  |  |
| **Human *CD40* promoter** |  |  |
| i) Pr3 Probe (carrying wild-type Runx site) | -473 to -444 | CCTCCCCATACCCCAGCTGTGGCCTTCCCG |
| ii) Mut-Pr3 probe (carrying mutant Runx site) | -473 to -444 | CCTCCCCATACCCCAGC*GAATTC*CTTCCCG |
| iii) Pr4 probe (carrying  wild-type Runx site) | -443 to -417 | TTTTCTGCGTGGTGGTGTGGGGGGAAC |
| iv) Mut-Pr4 probe (carrying  mutant Runx site) | -443 to -417 | TTTTCTGCGT*GAATTC*GTGGGGGGAAC |

All position numbers are relative to the transcription start sites; binding sites for Runx transcription factors are underlined and mutated bases are italicized.
